# Supplementary figures and images for: Microbiota from young mice counteracts susceptibility to age-related gout through modulating butyric acid levels in aged mice
Source: eLife. 2025 Feb 5;13:RP98714. doi: 10.7554/eLife.98714 (PMC11798573; doi:10.7554/eLife.98714)

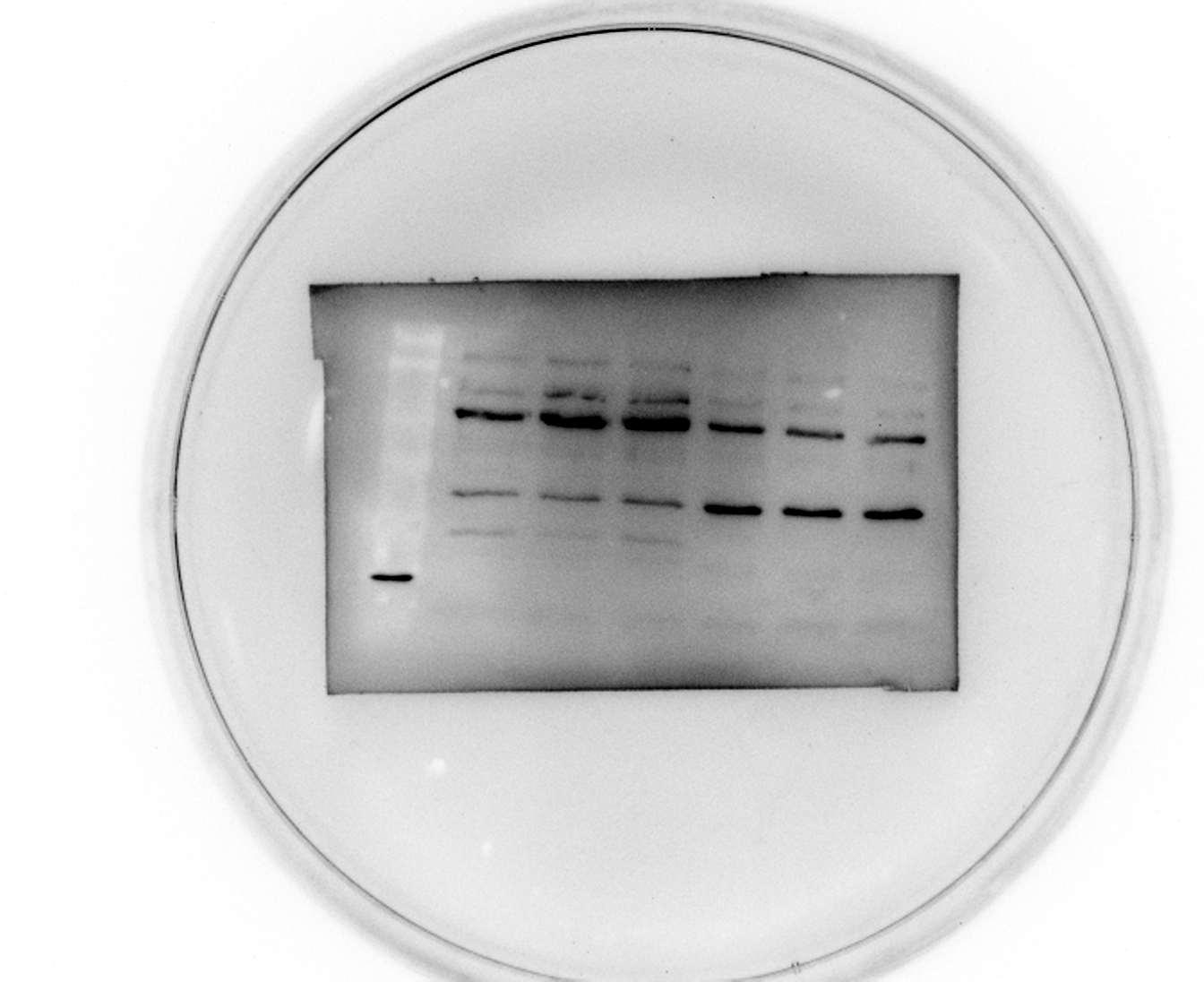

Supplement: Figure 3—source data 2. [file elife-98714-fig3-data2.zip › FIG3 source data-FOOT TISSUE/CASPASE-1/casepase of A-AY.Tif]

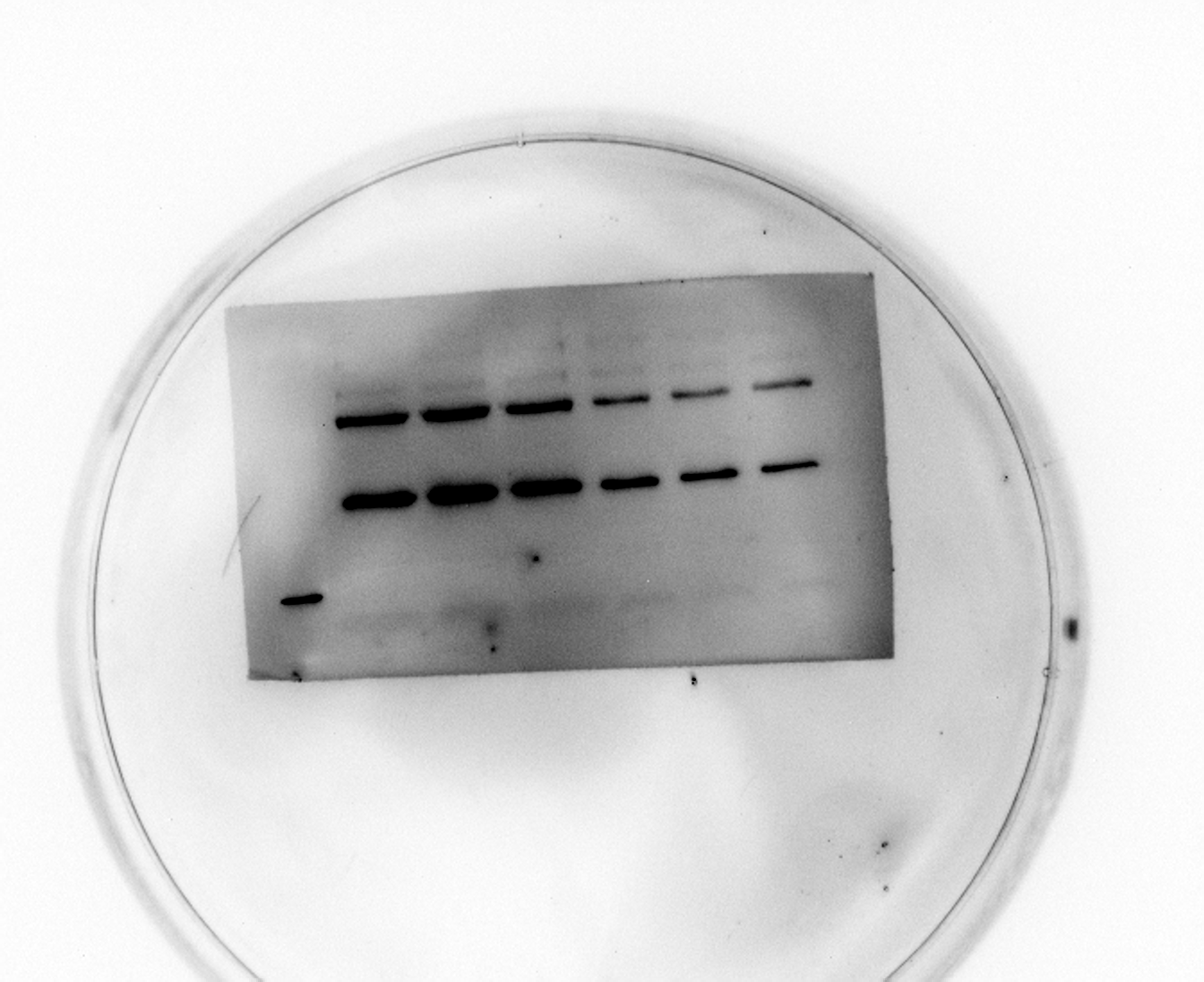

Supplement: Figure 3—source data 2. [file elife-98714-fig3-data2.zip › FIG3 source data-FOOT TISSUE/CASPASE-1/caspase of O-OY.Tif]

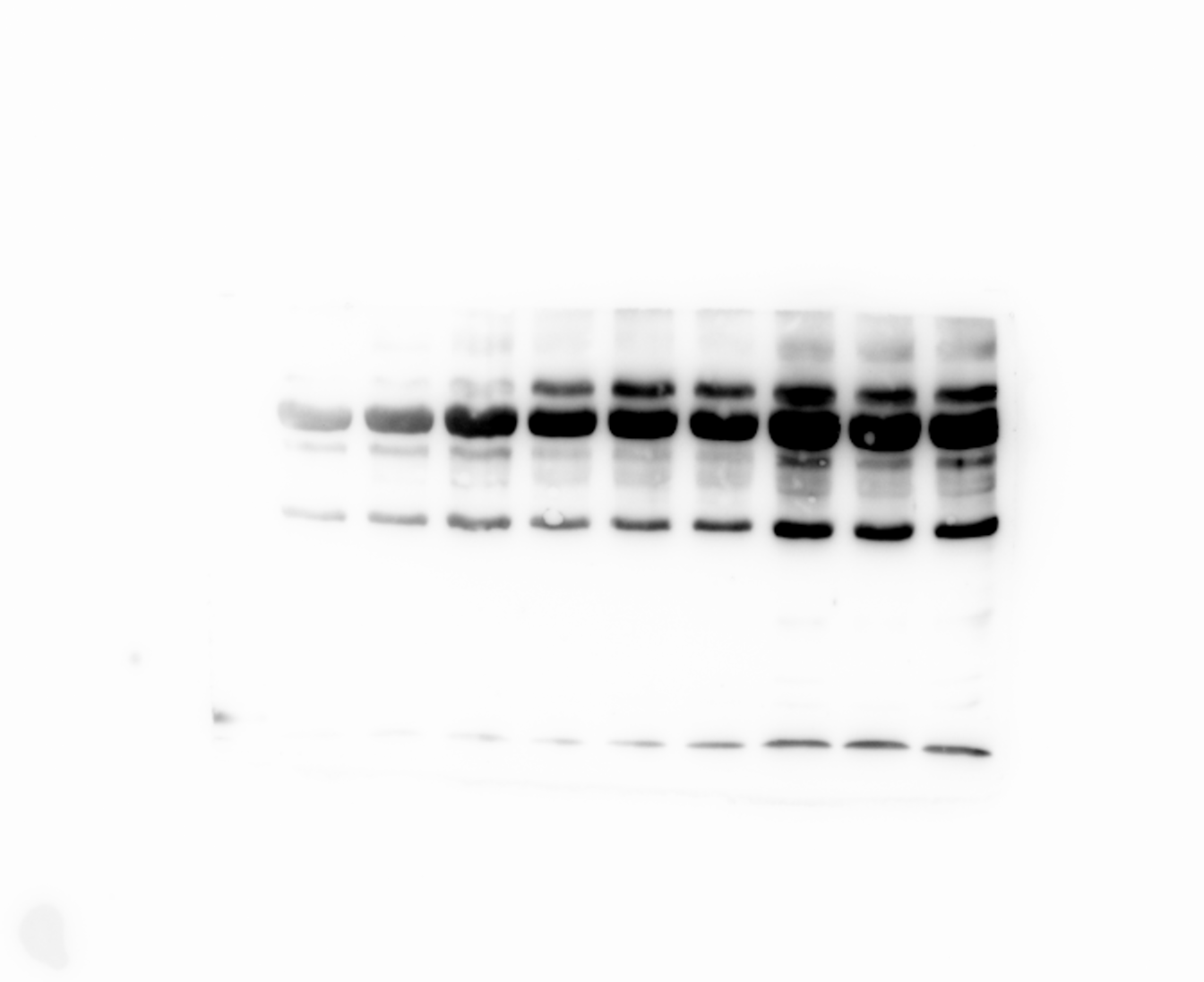

Supplement: Figure 3—source data 2. [file elife-98714-fig3-data2.zip › FIG3 source data-FOOT TISSUE/CASPASE-1/caspase of Y-Y.O-Y.A.Tif]

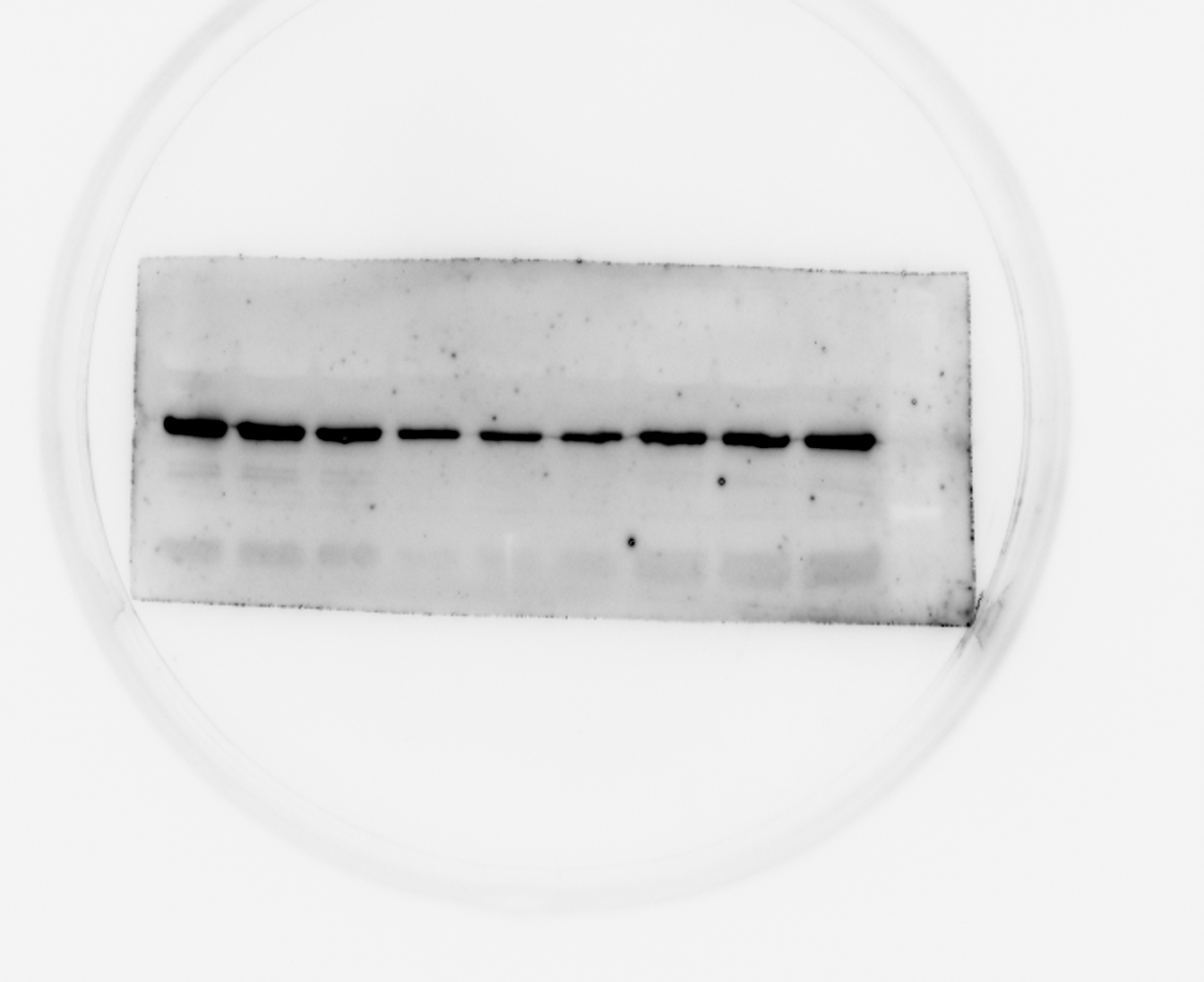

Supplement: Figure 3—source data 2. [file elife-98714-fig3-data2.zip › FIG3 source data-FOOT TISSUE/IL-1a┬/IL-1a┬ of Y-Y.O-Y.A.Tif]

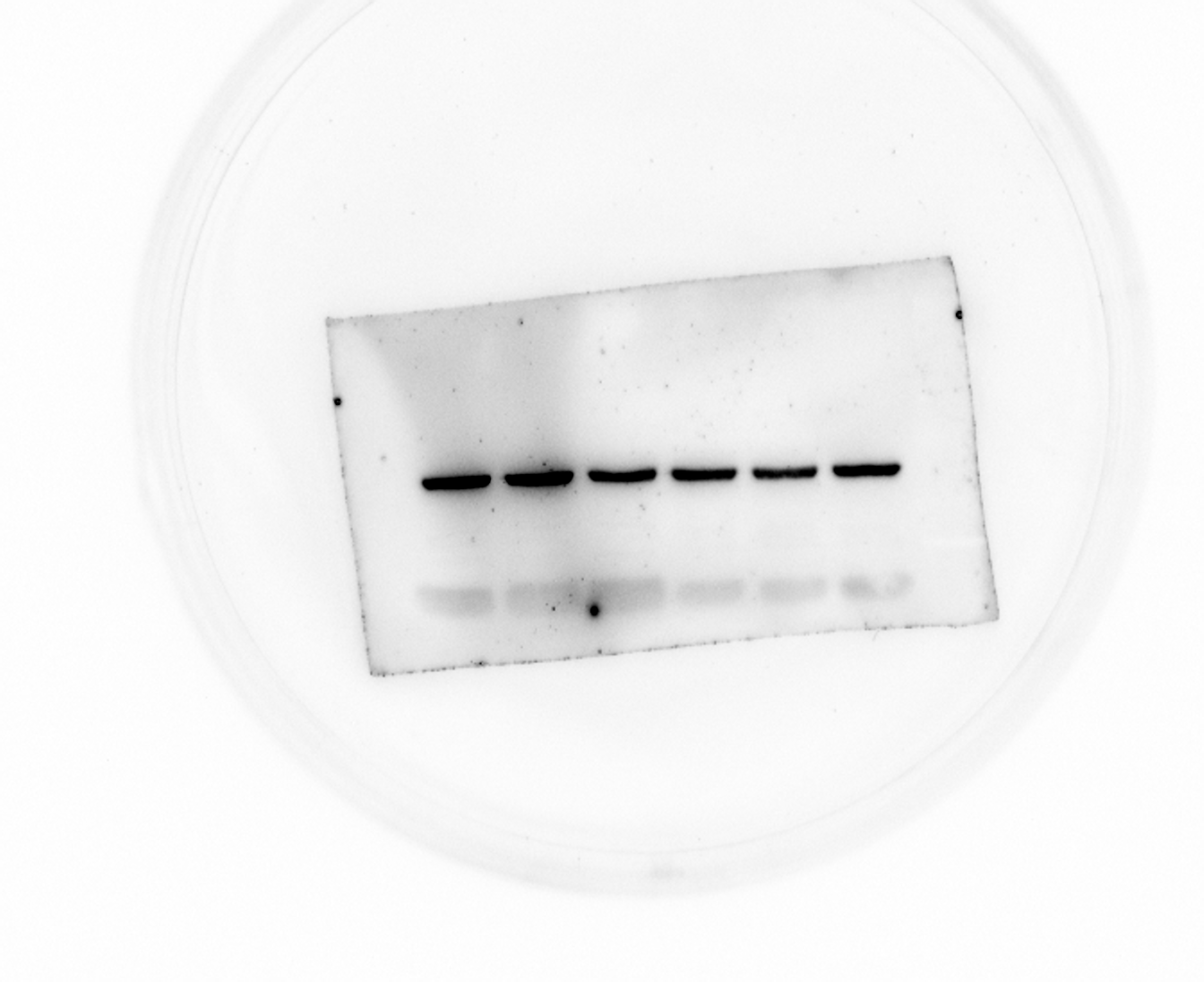

Supplement: Figure 3—source data 2. [file elife-98714-fig3-data2.zip › FIG3 source data-FOOT TISSUE/IL-1a┬/IL-1a┬ of A-AY.Tif]

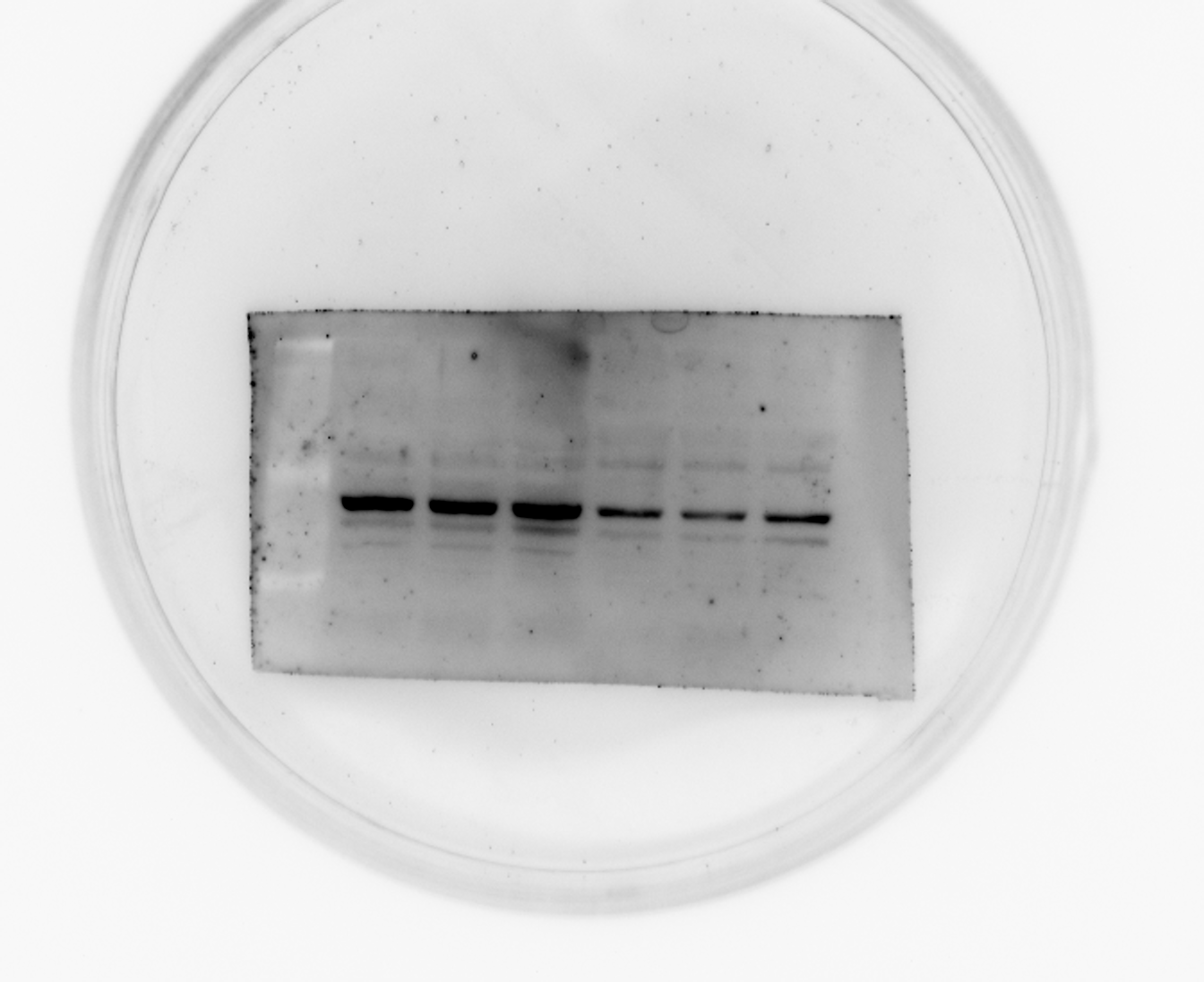

Supplement: Figure 3—source data 2. [file elife-98714-fig3-data2.zip › FIG3 source data-FOOT TISSUE/IL-1a┬/Il-1a┬ of O-OY.Tif]

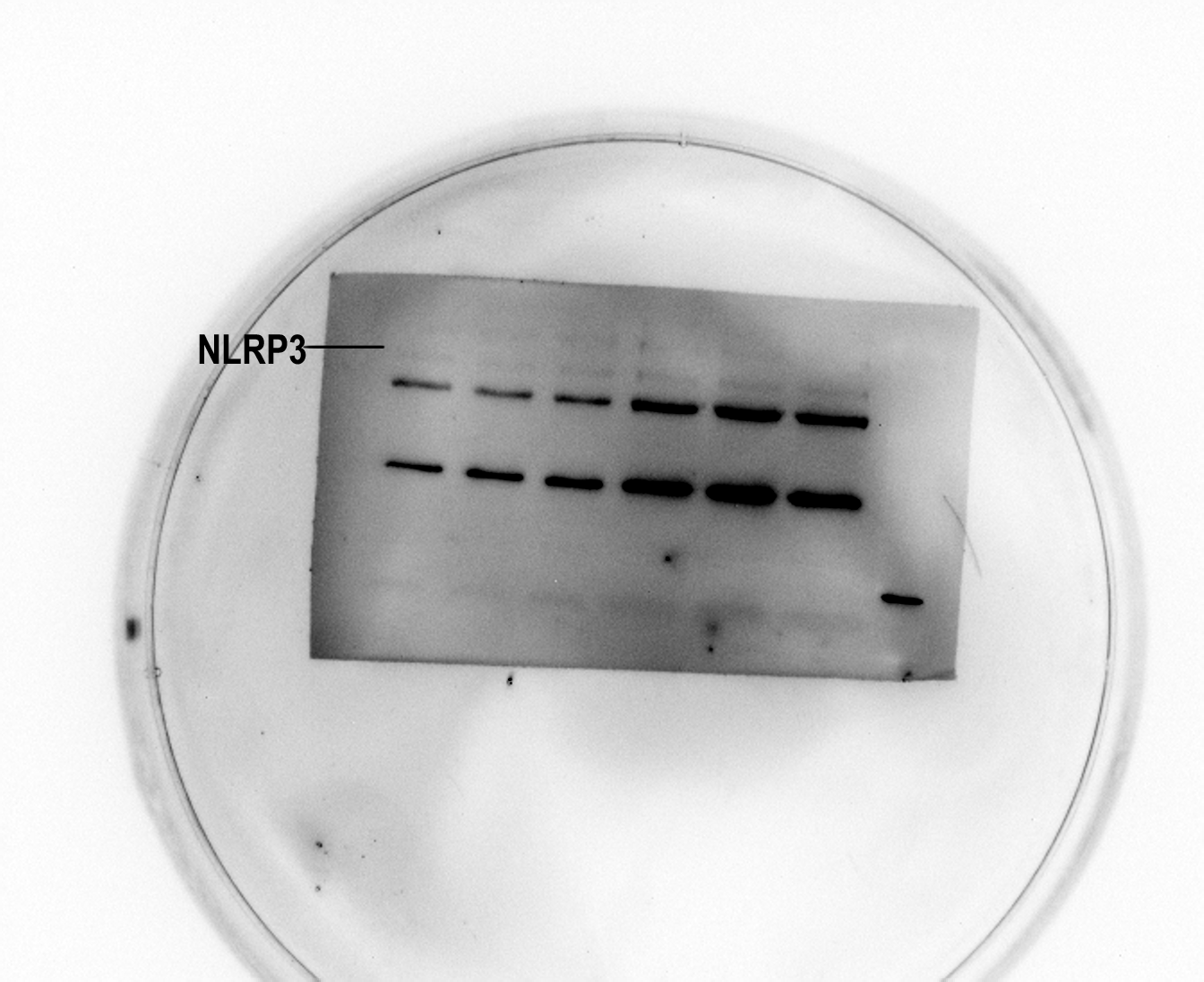

Supplement: Figure 3—source data 2. [file elife-98714-fig3-data2.zip › FIG3 source data-FOOT TISSUE/NLRP3/NLRP3 of A-AY.Tif]

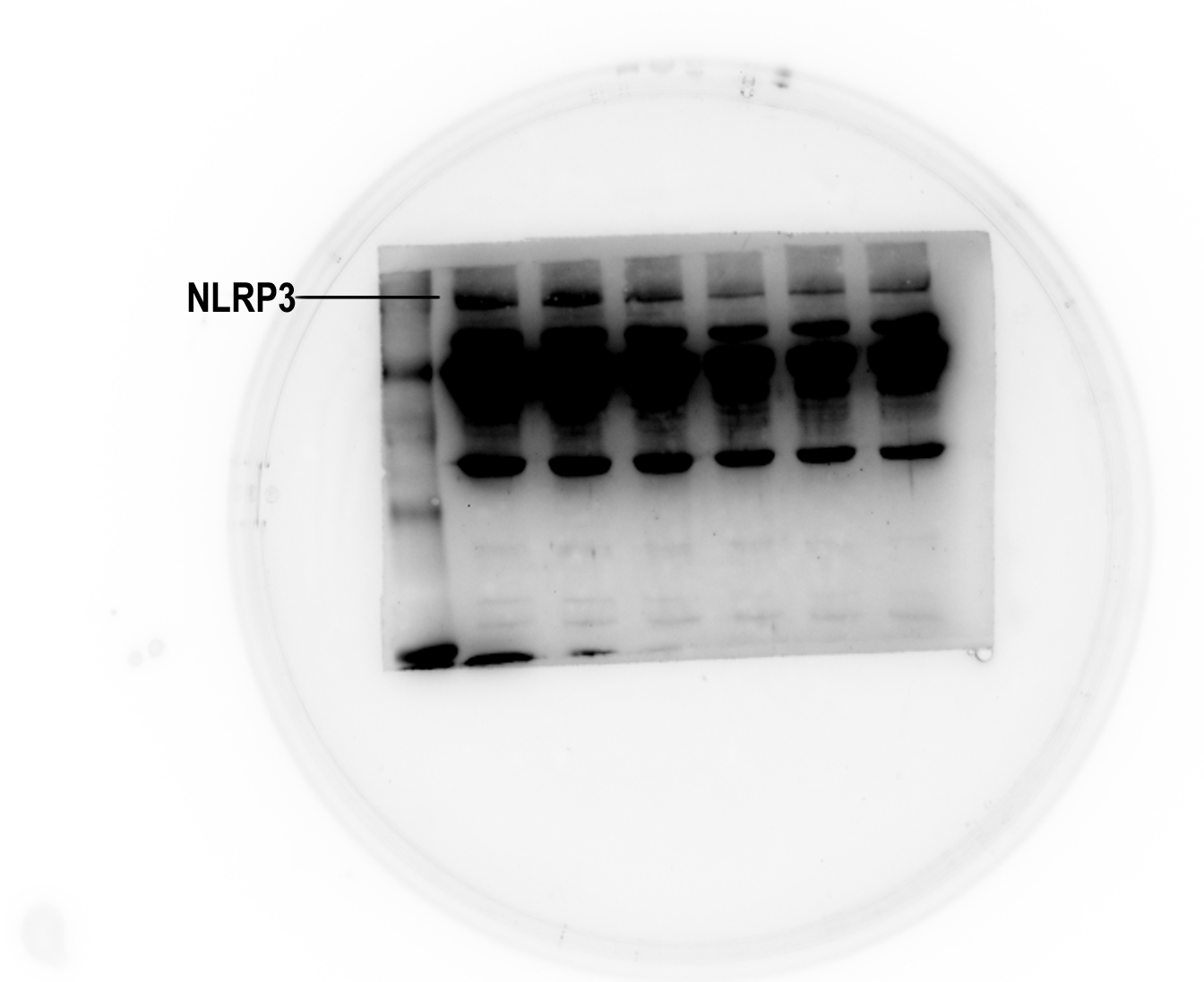

Supplement: Figure 3—source data 2. [file elife-98714-fig3-data2.zip › FIG3 source data-FOOT TISSUE/NLRP3/NLRP3 of O-OY.Tif]

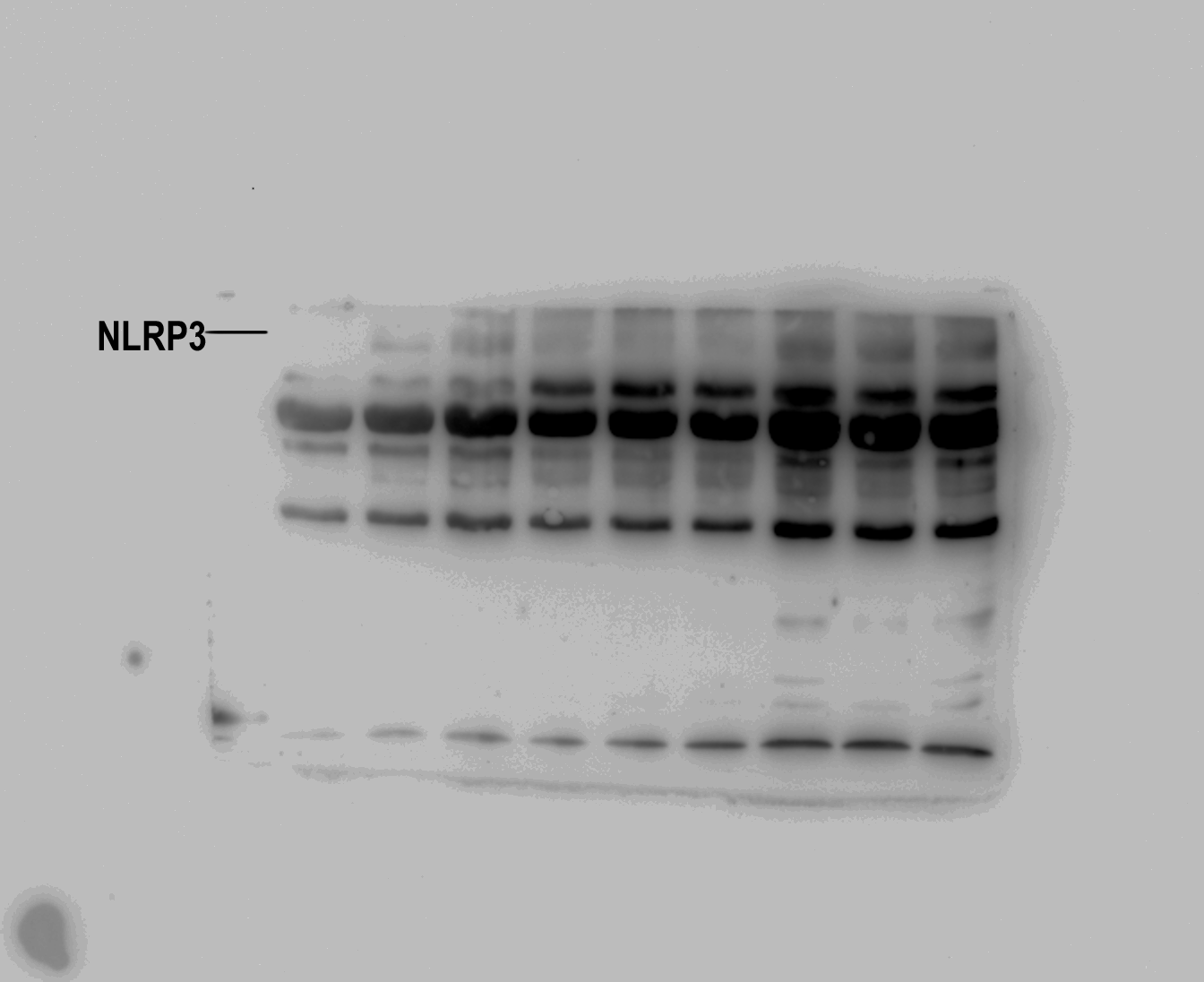

Supplement: Figure 3—source data 2. [file elife-98714-fig3-data2.zip › FIG3 source data-FOOT TISSUE/NLRP3/NLRP3 of Y-Y.O-Y.A.Tif]

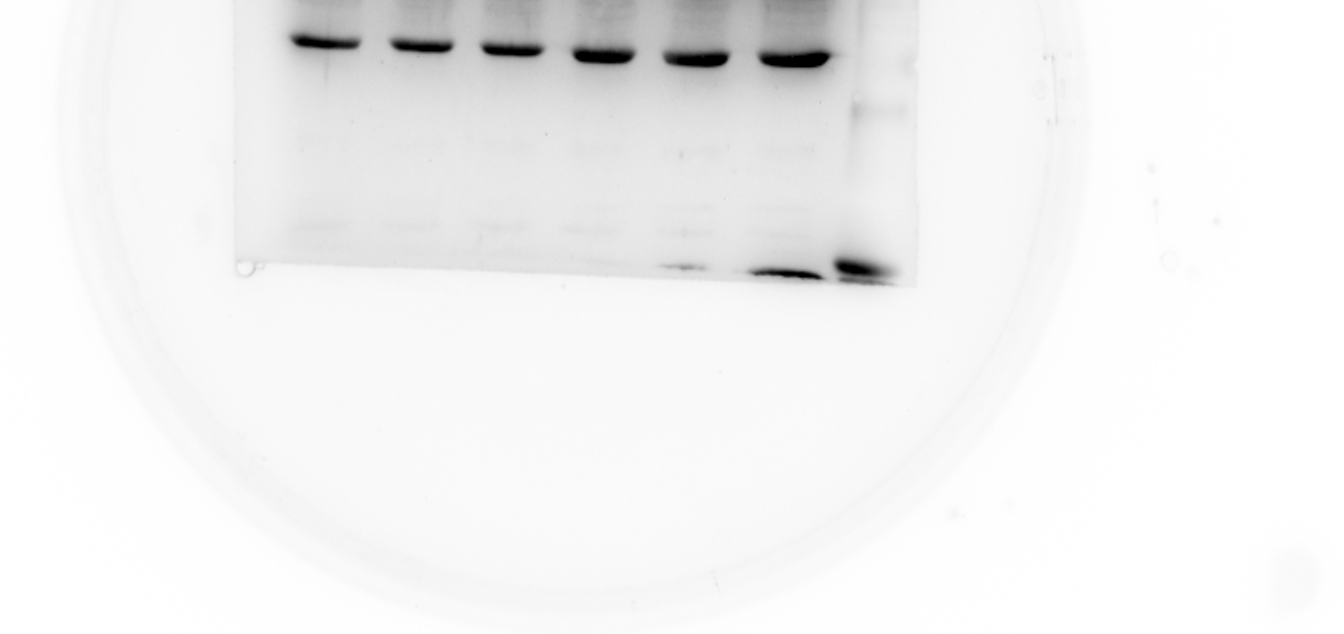

Supplement: Figure 3—source data 2. [file elife-98714-fig3-data2.zip › FIG3 source data-FOOT TISSUE/a┬-actin/a┬-actin of A-AY.Tif]

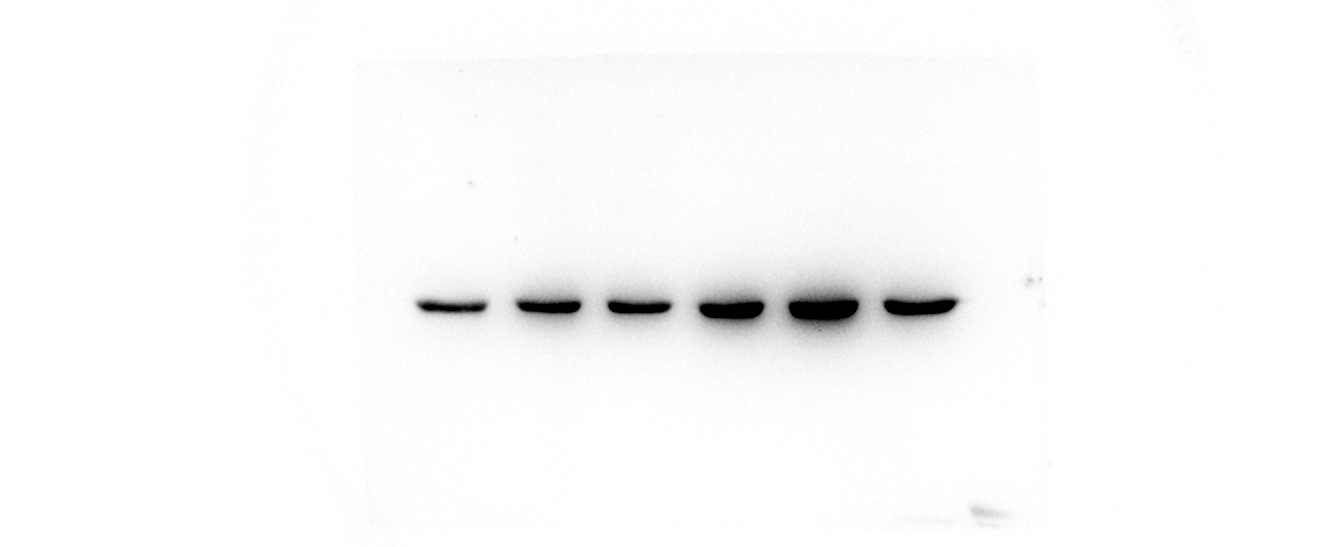

Supplement: Figure 3—source data 2. [file elife-98714-fig3-data2.zip › FIG3 source data-FOOT TISSUE/a┬-actin/a┬-actin of O-OY.Tif]

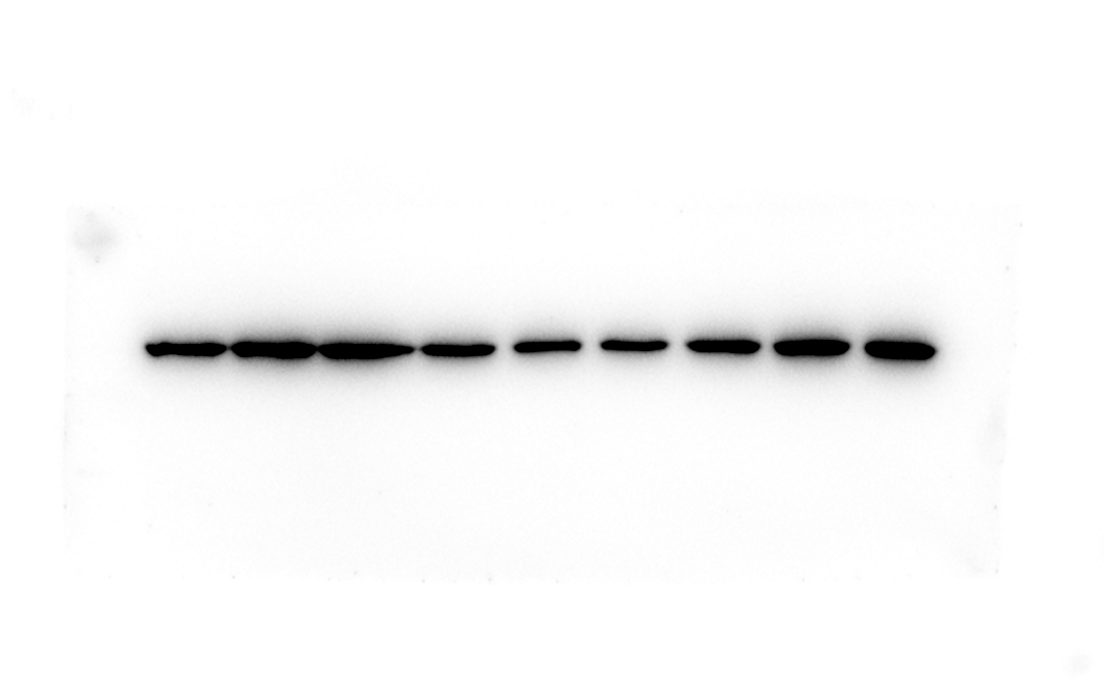

Supplement: Figure 3—source data 2. [file elife-98714-fig3-data2.zip › FIG3 source data-FOOT TISSUE/a┬-actin/a┬-actin of Y-Y.O-Y.A.Tif]

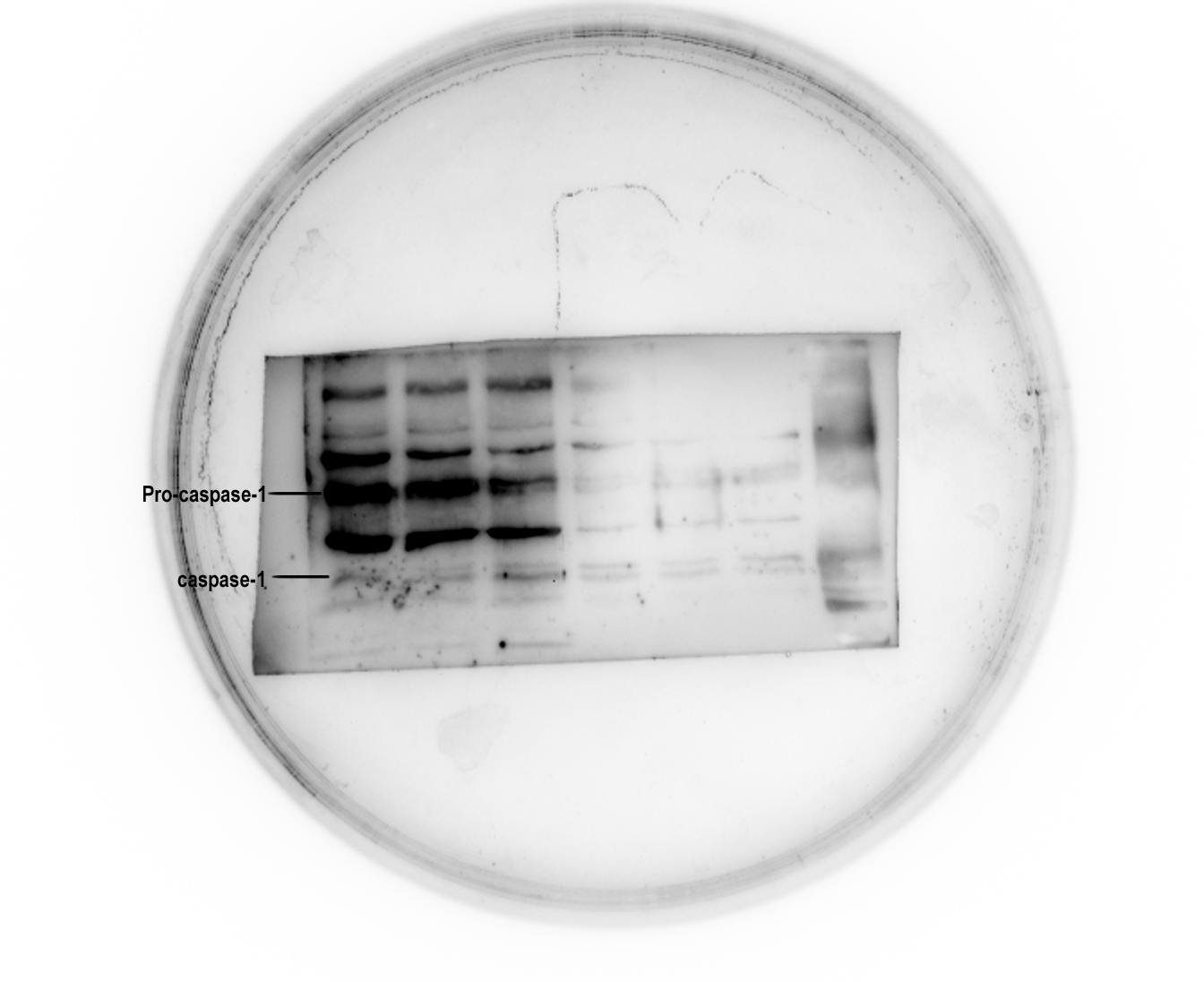

Supplement: Figure 3—figure supplement 1—source data 2. [file elife-98714-fig3-figsupp1-data2.zip › FIG3 figure supplements 1 source data-cell/CASPASE-1/caspase-1 of A-AY.Tif]

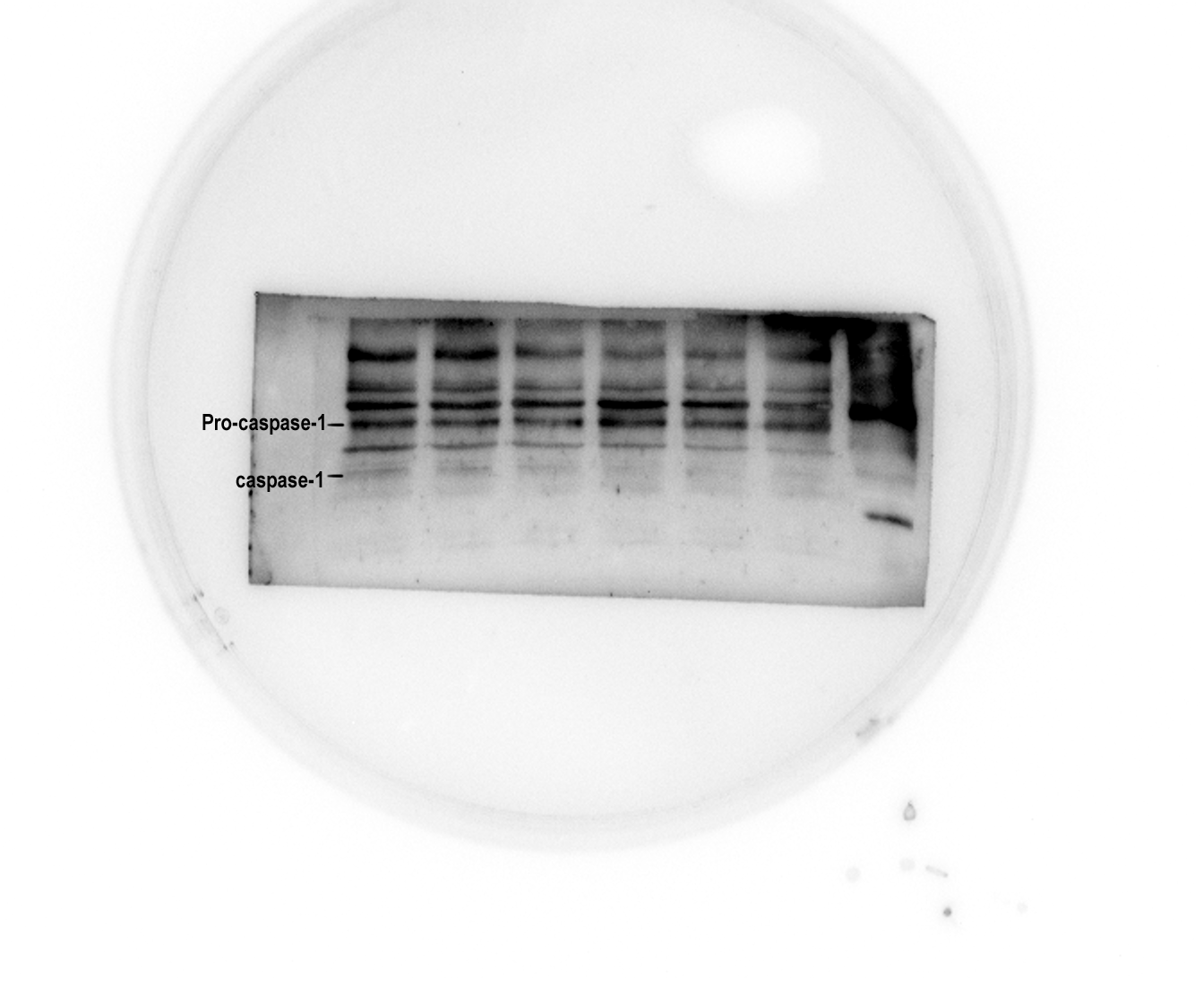

Supplement: Figure 3—figure supplement 1—source data 2. [file elife-98714-fig3-figsupp1-data2.zip › FIG3 figure supplements 1 source data-cell/CASPASE-1/caspase-1 of O-OY.Tif]

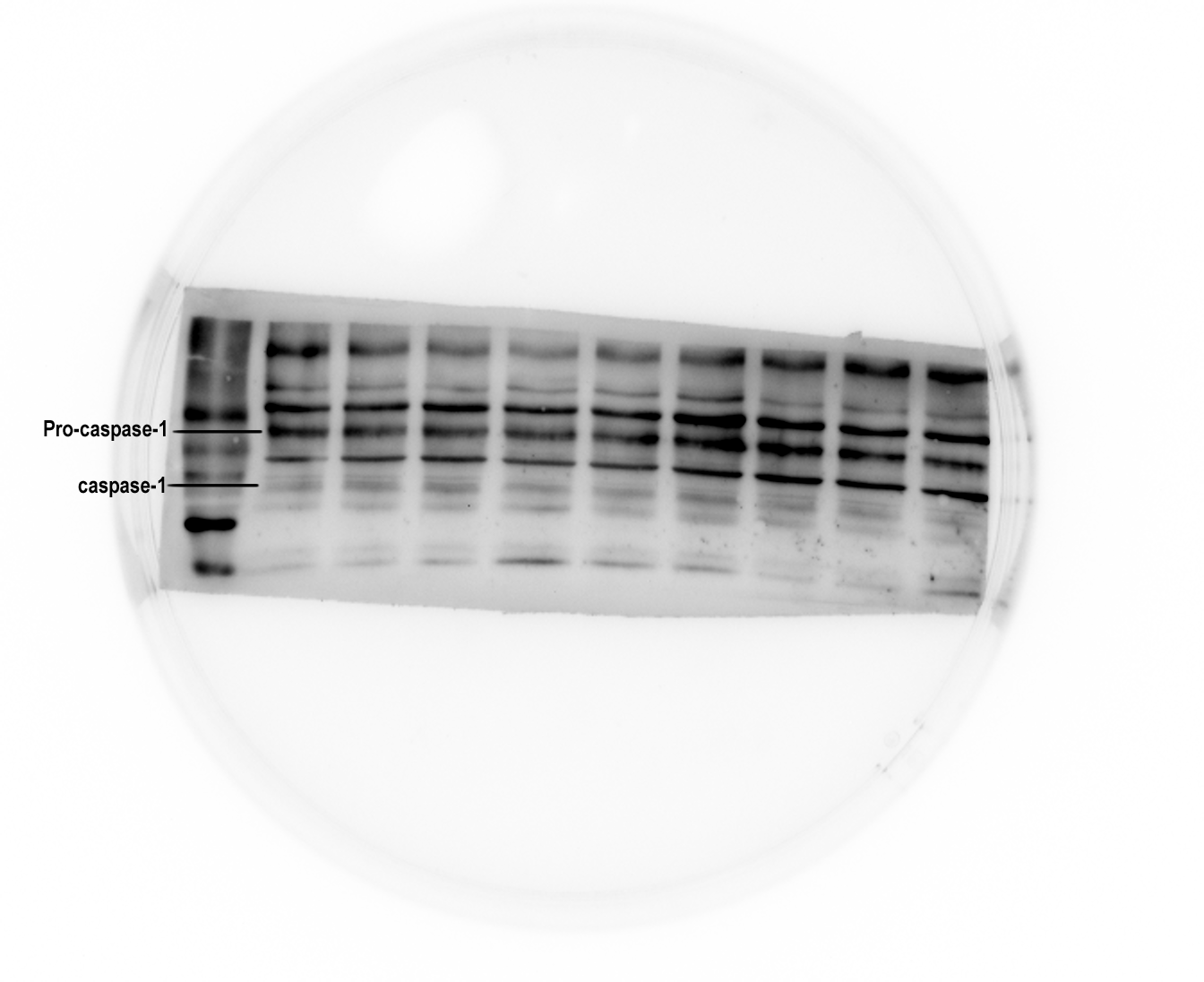

Supplement: Figure 3—figure supplement 1—source data 2. [file elife-98714-fig3-figsupp1-data2.zip › FIG3 figure supplements 1 source data-cell/CASPASE-1/caspase-1 of Y-Y.O-Y.A.Tif]

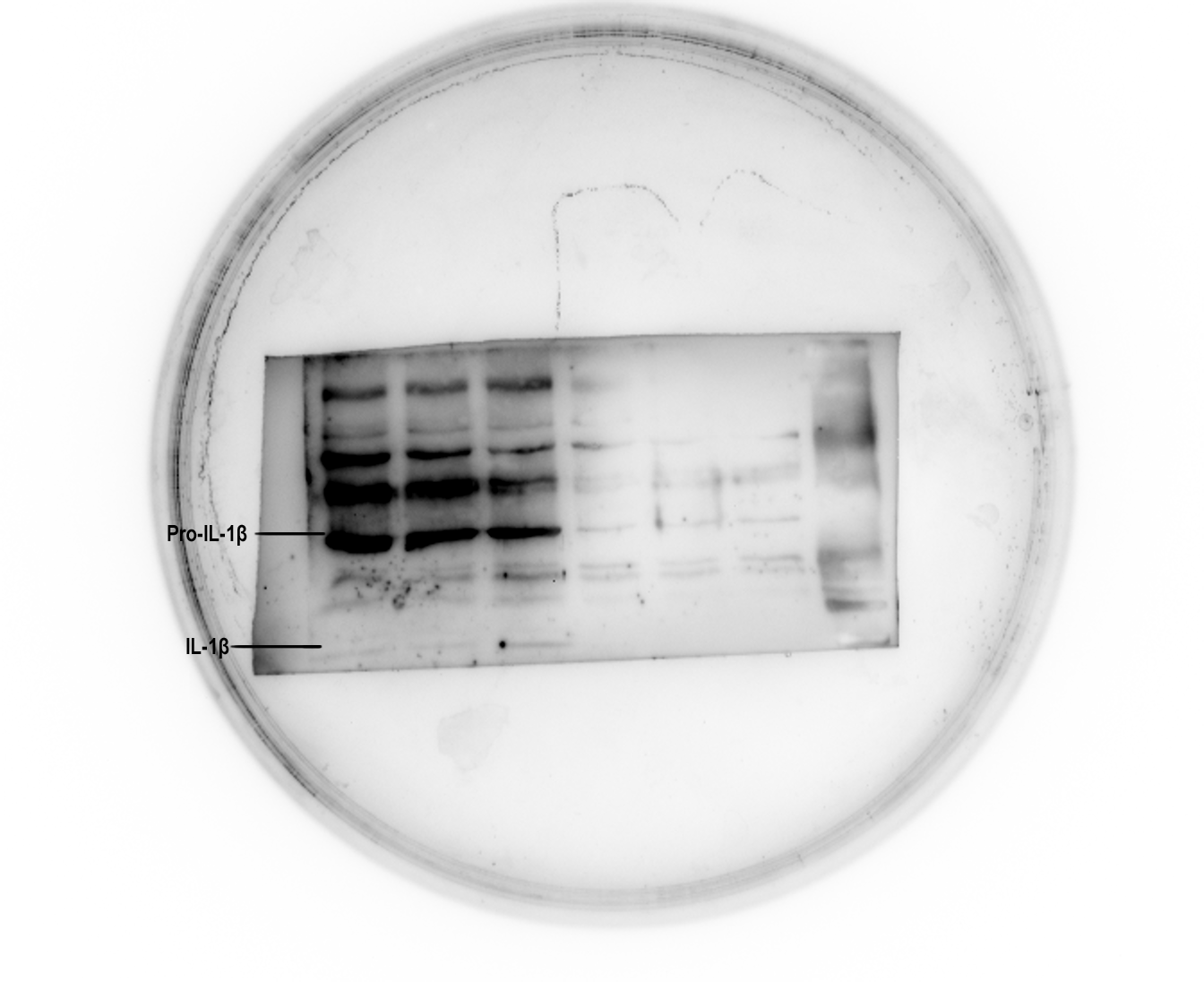

Supplement: Figure 3—figure supplement 1—source data 2. [file elife-98714-fig3-figsupp1-data2.zip › FIG3 figure supplements 1 source data-cell/IL-1a┬/IL-1a┬ of A-AY.Tif]

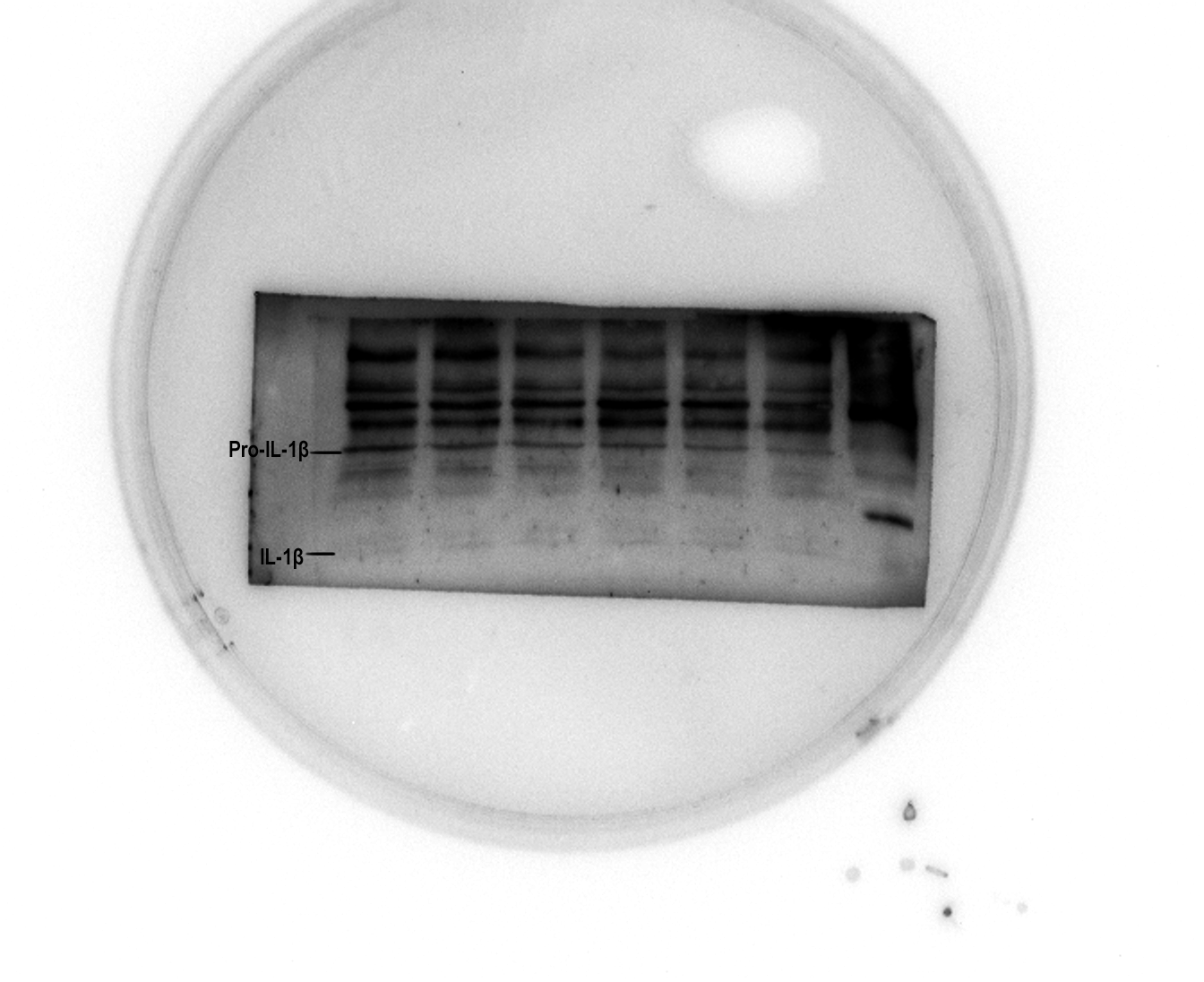

Supplement: Figure 3—figure supplement 1—source data 2. [file elife-98714-fig3-figsupp1-data2.zip › FIG3 figure supplements 1 source data-cell/IL-1a┬/IL-1a┬ of O-OY.Tif]

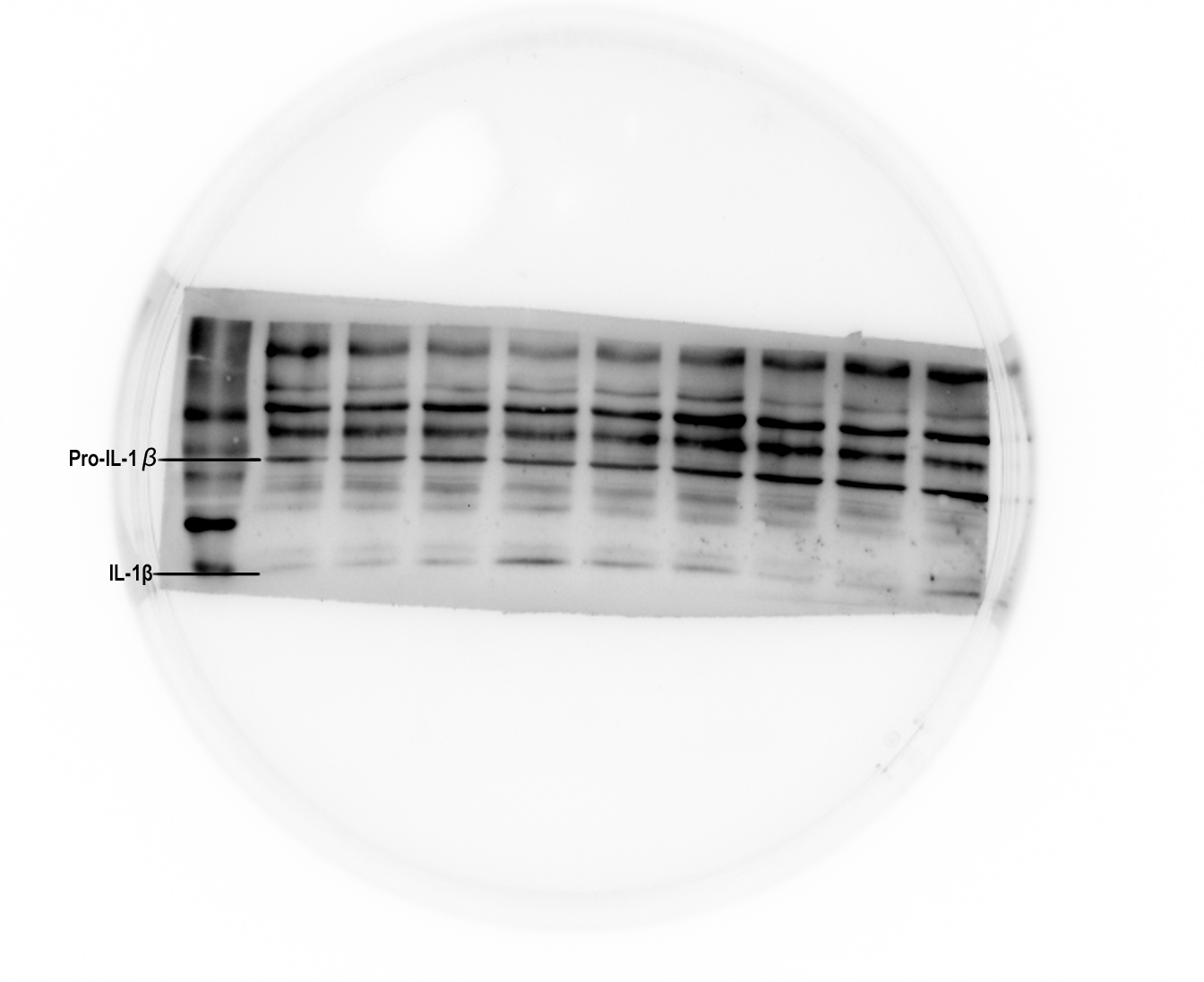

Supplement: Figure 3—figure supplement 1—source data 2. [file elife-98714-fig3-figsupp1-data2.zip › FIG3 figure supplements 1 source data-cell/IL-1a┬/IL-1a┬ of Y-Y.O-Y.A.Tif]

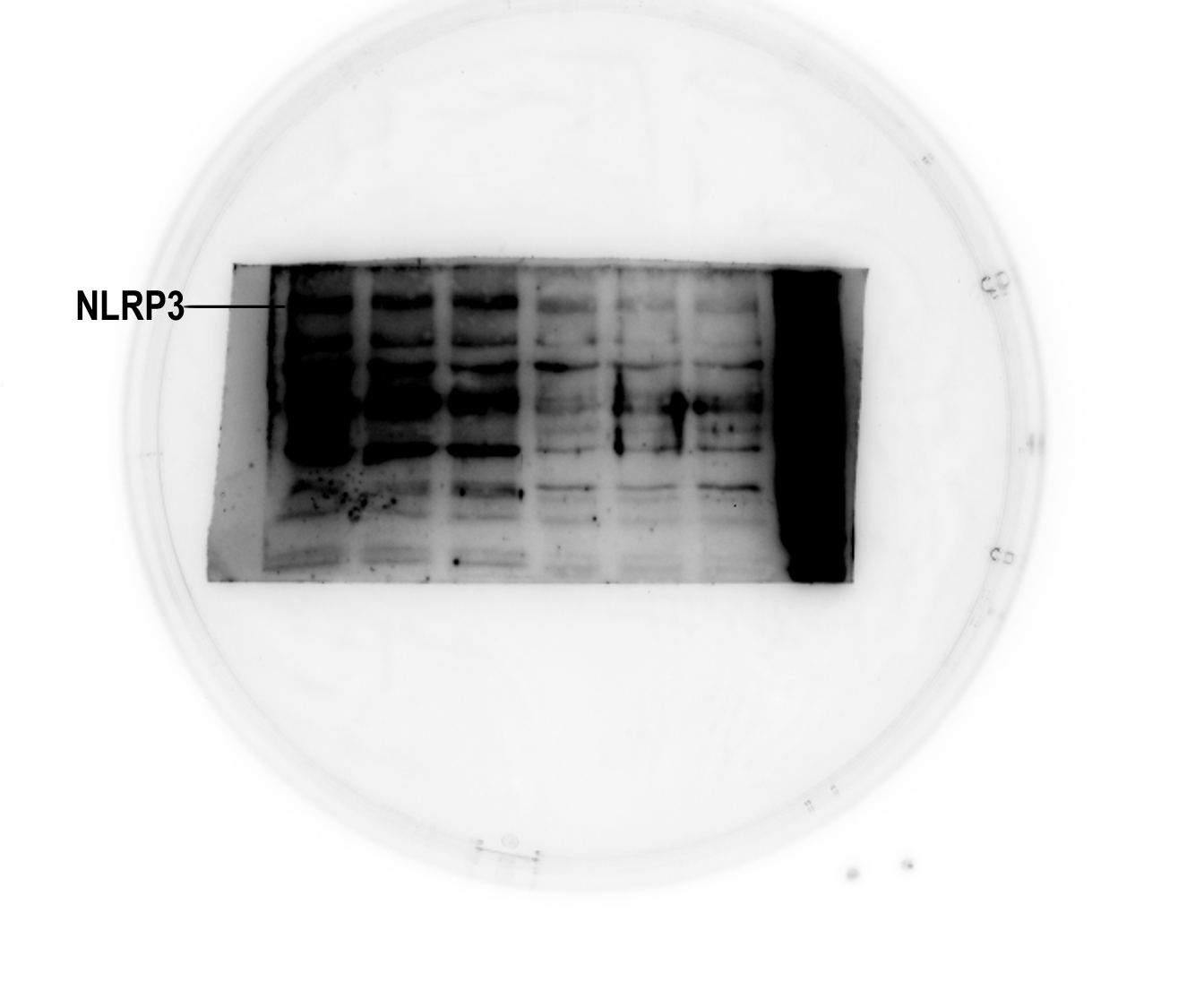

Supplement: Figure 3—figure supplement 1—source data 2. [file elife-98714-fig3-figsupp1-data2.zip › FIG3 figure supplements 1 source data-cell/NLRP3/NLRP3 of A-AY.Tif]

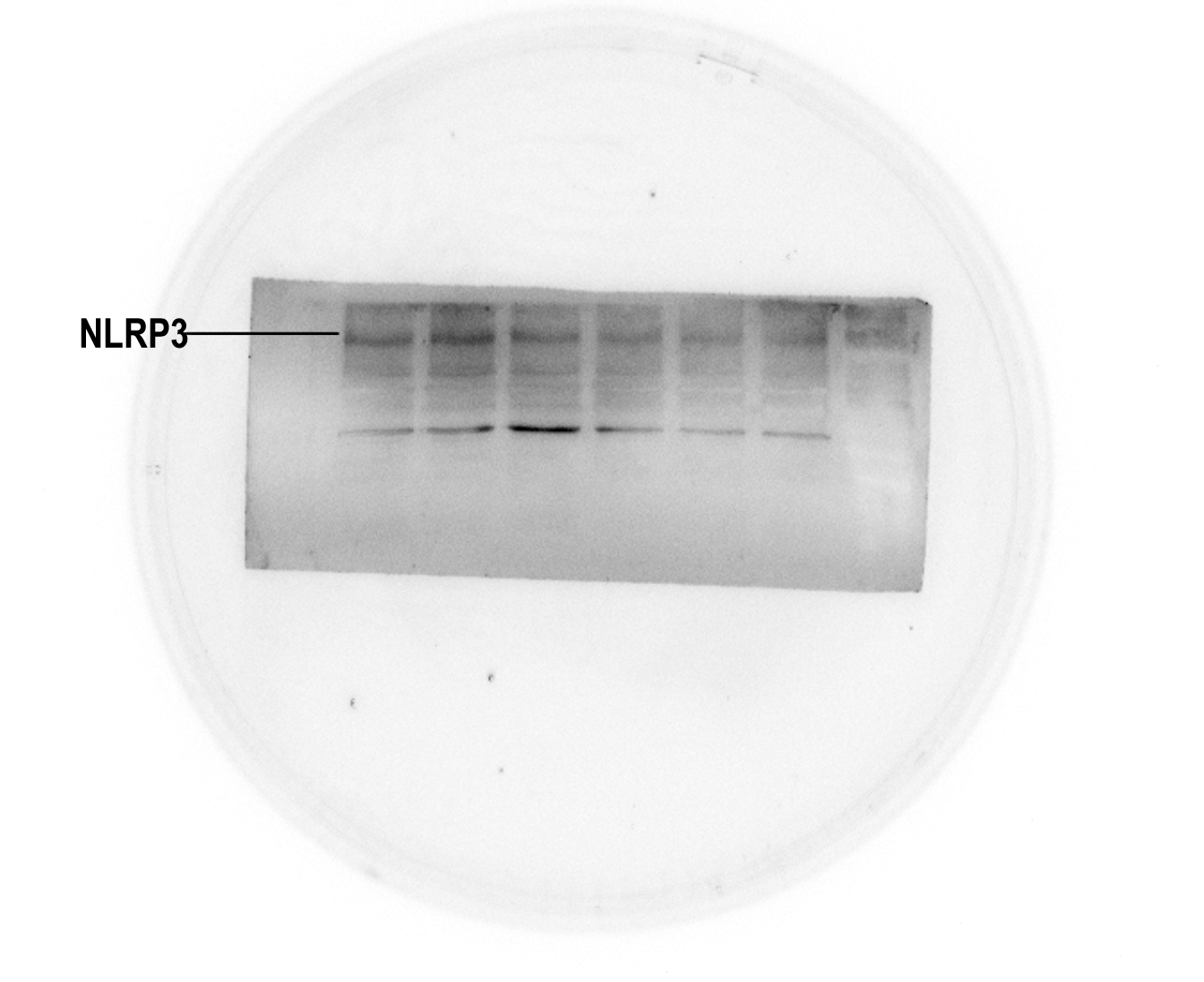

Supplement: Figure 3—figure supplement 1—source data 2. [file elife-98714-fig3-figsupp1-data2.zip › FIG3 figure supplements 1 source data-cell/NLRP3/NLRP3 of O-OY.Tif]

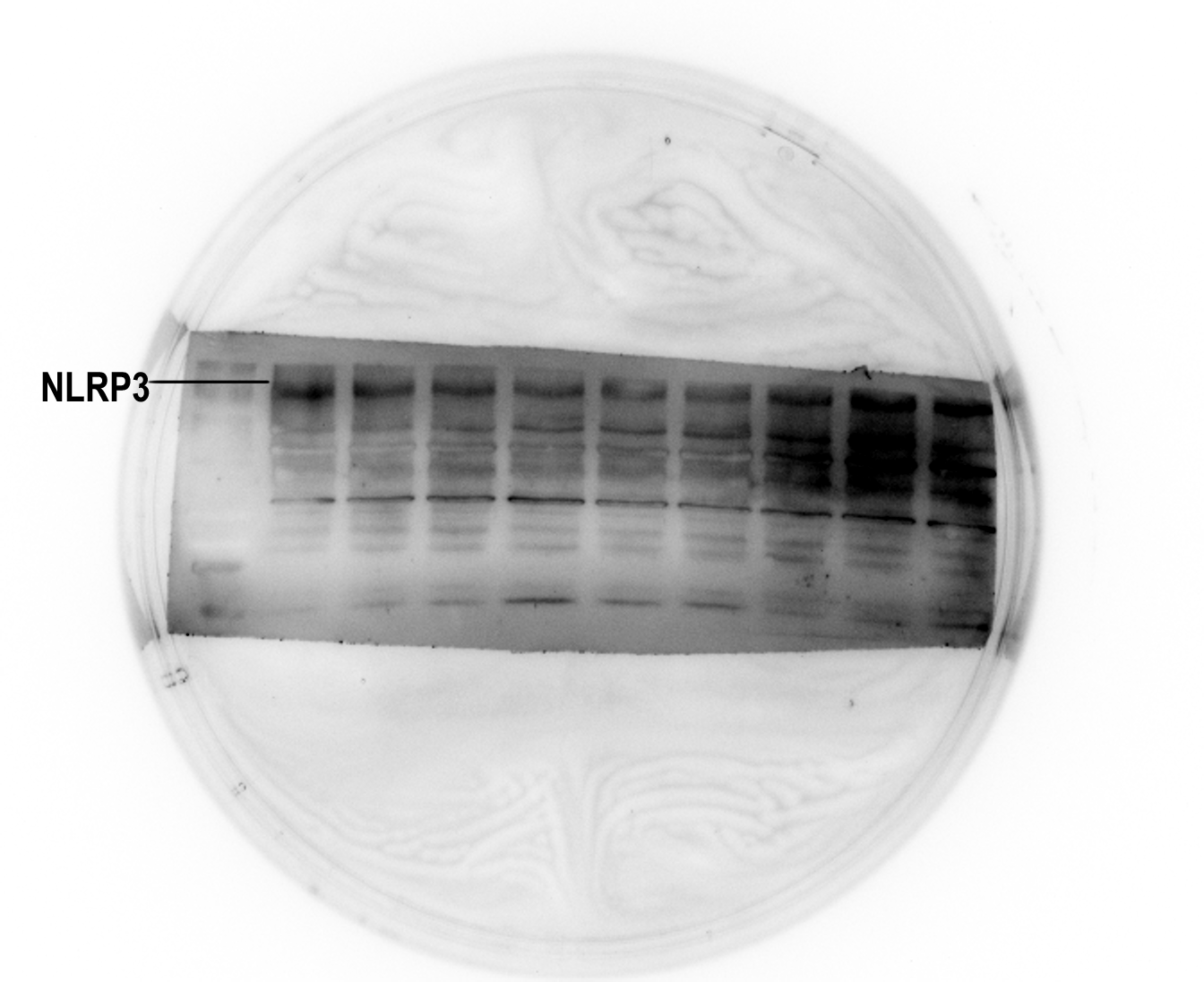

Supplement: Figure 3—figure supplement 1—source data 2. [file elife-98714-fig3-figsupp1-data2.zip › FIG3 figure supplements 1 source data-cell/NLRP3/NLRP3 of Y-Y.O-Y.A.Tif]

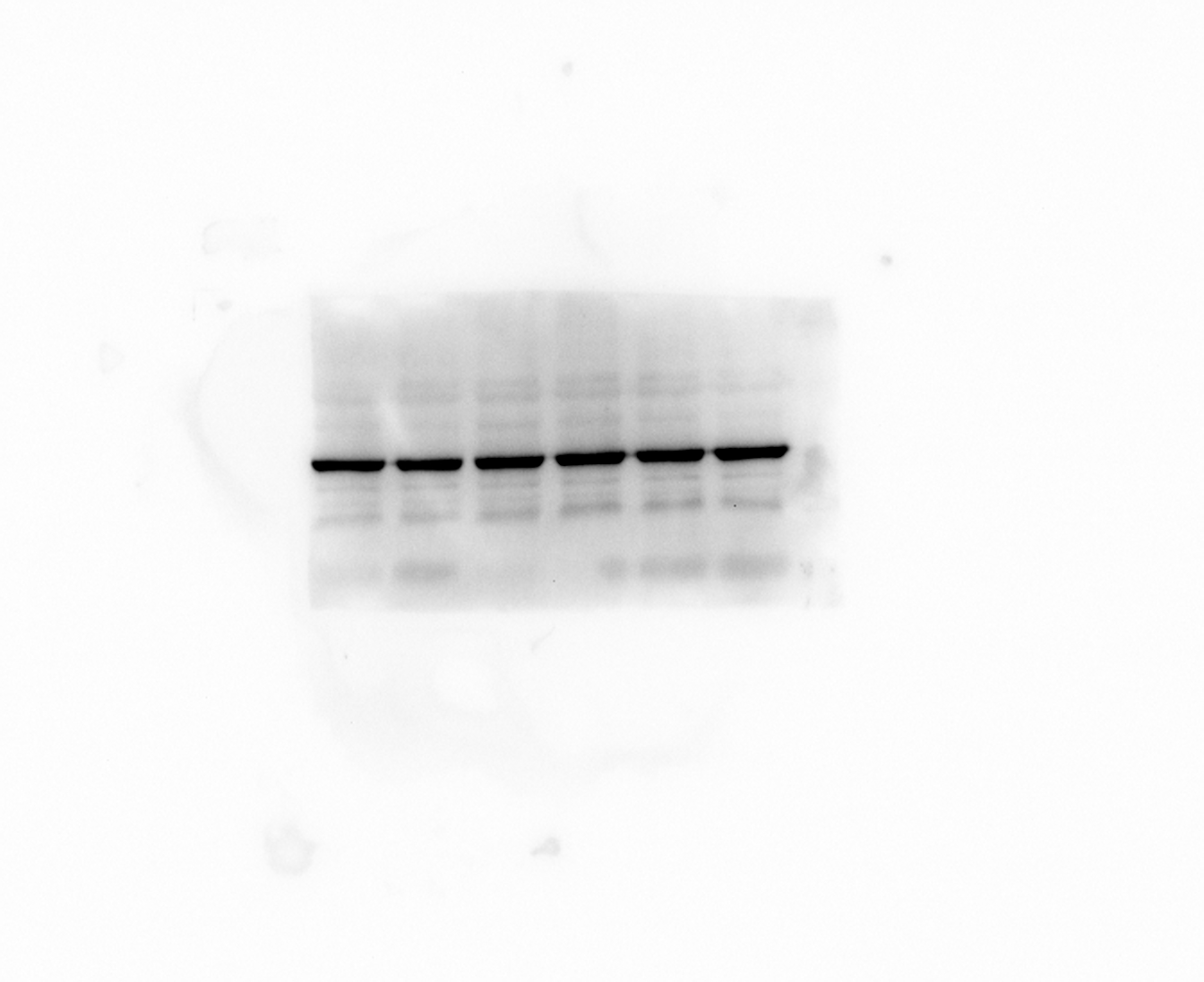

Supplement: Figure 3—figure supplement 1—source data 2. [file elife-98714-fig3-figsupp1-data2.zip › FIG3 figure supplements 1 source data-cell/a┬-actin/a┬-actin of A-AY.jpg]

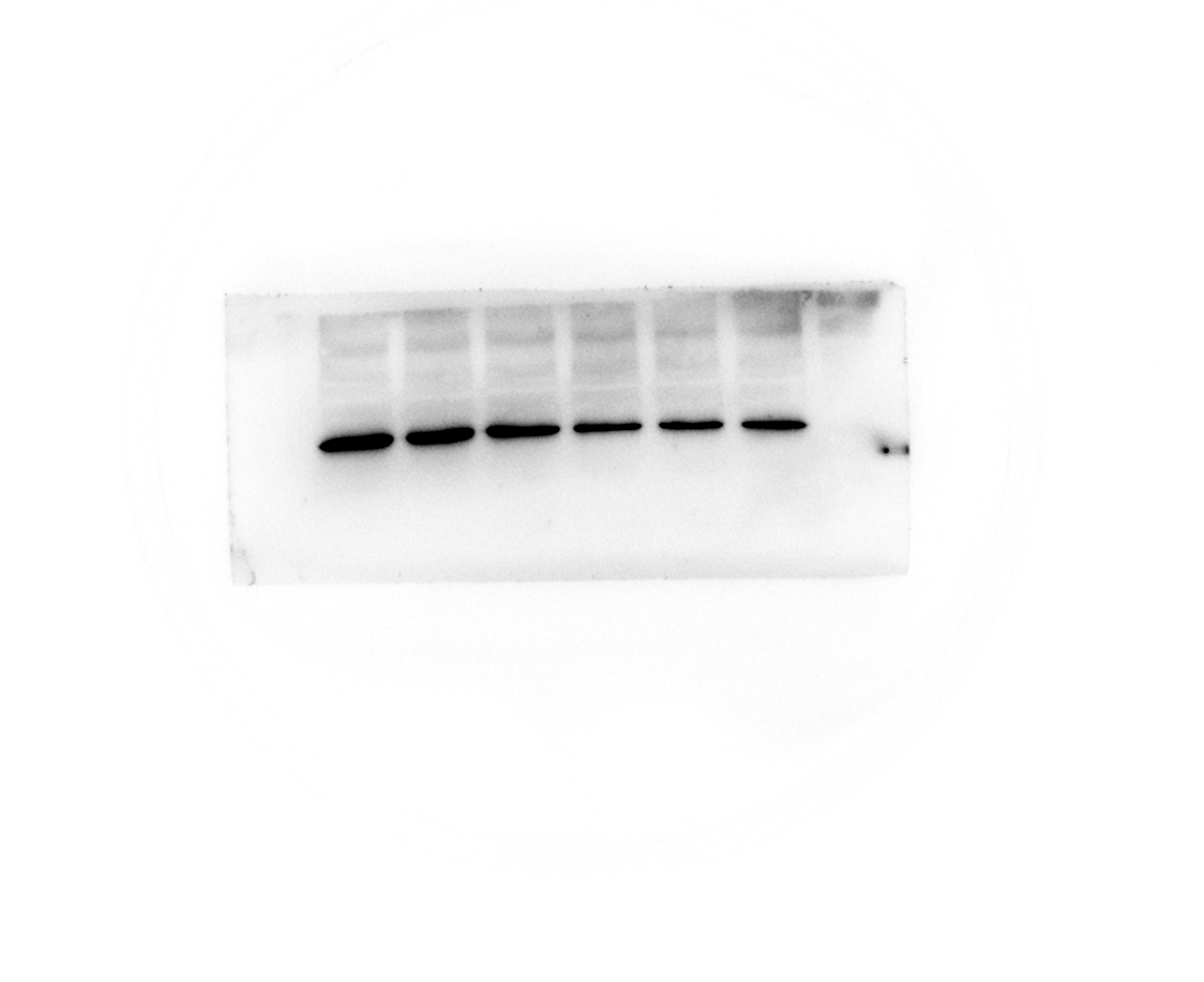

Supplement: Figure 3—figure supplement 1—source data 2. [file elife-98714-fig3-figsupp1-data2.zip › FIG3 figure supplements 1 source data-cell/a┬-actin/a┬-actin of O-OY.Tif]

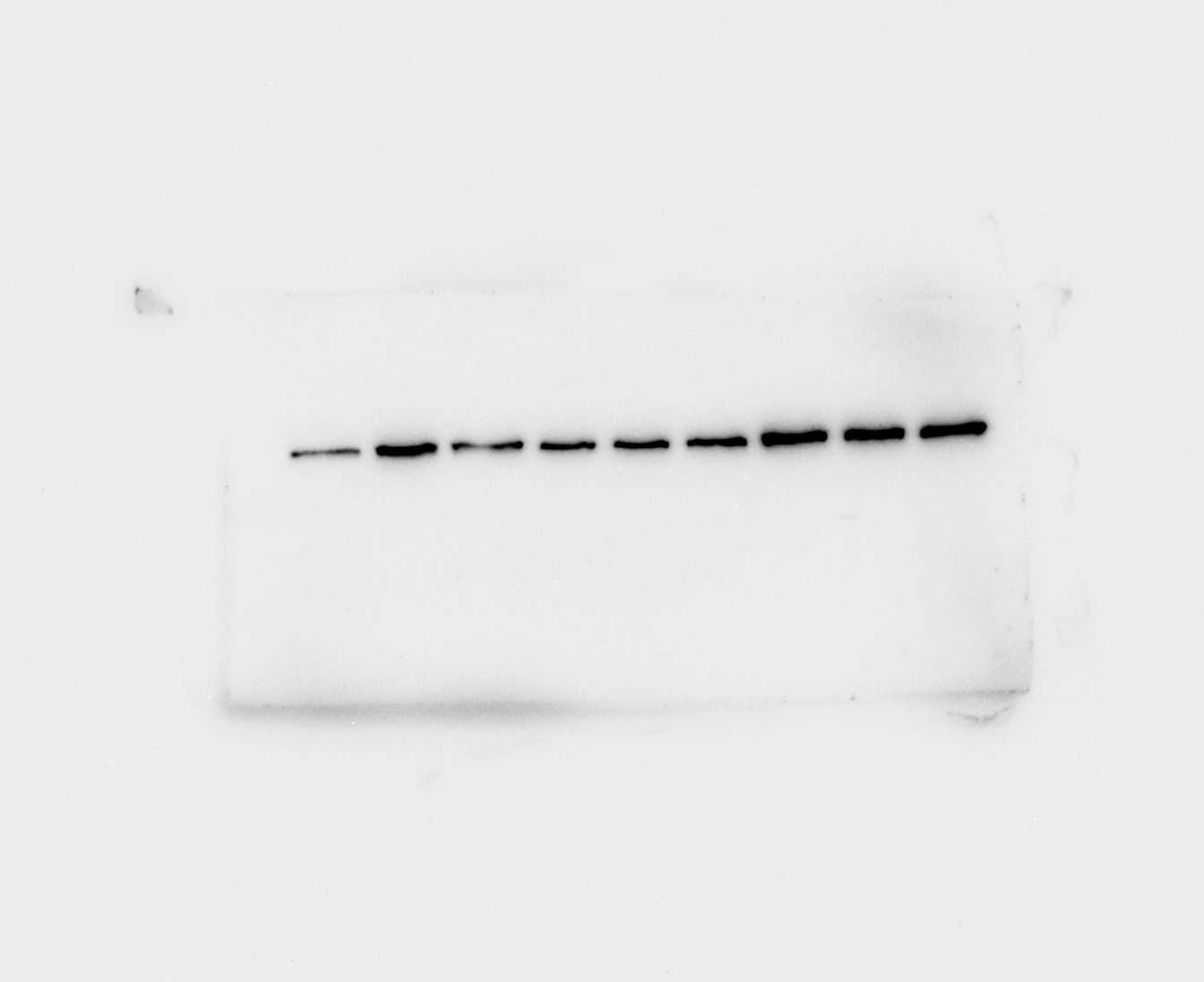

Supplement: Figure 3—figure supplement 1—source data 2. [file elife-98714-fig3-figsupp1-data2.zip › FIG3 figure supplements 1 source data-cell/a┬-actin/a┬-actin of Y-Y.O-Y.A.Tif]

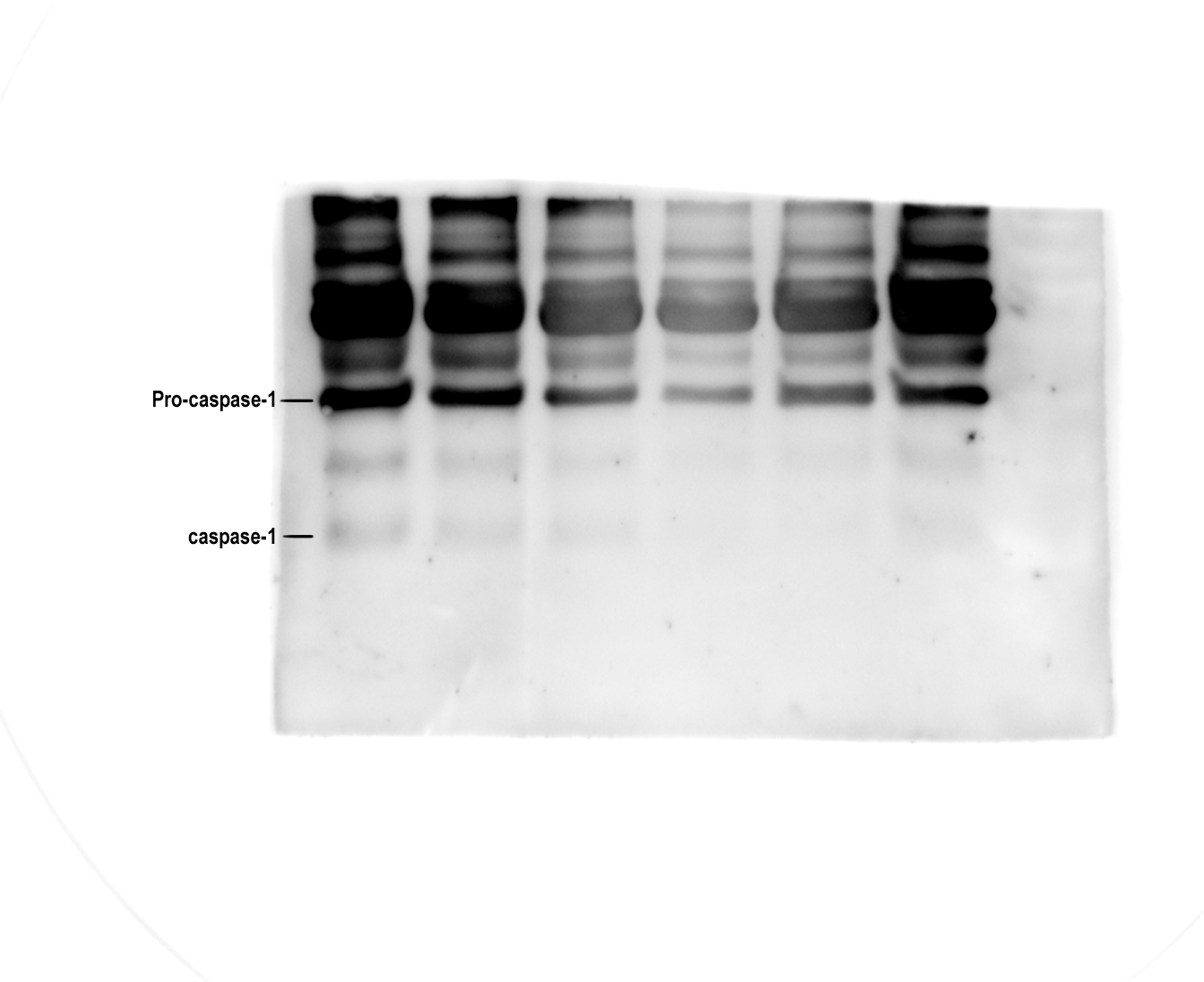

Supplement: Figure 7—source data 2. [file elife-98714-fig7-data2.zip › FIG7 source data-foot tissue/CASPASE-1/caspase-1.Tif]

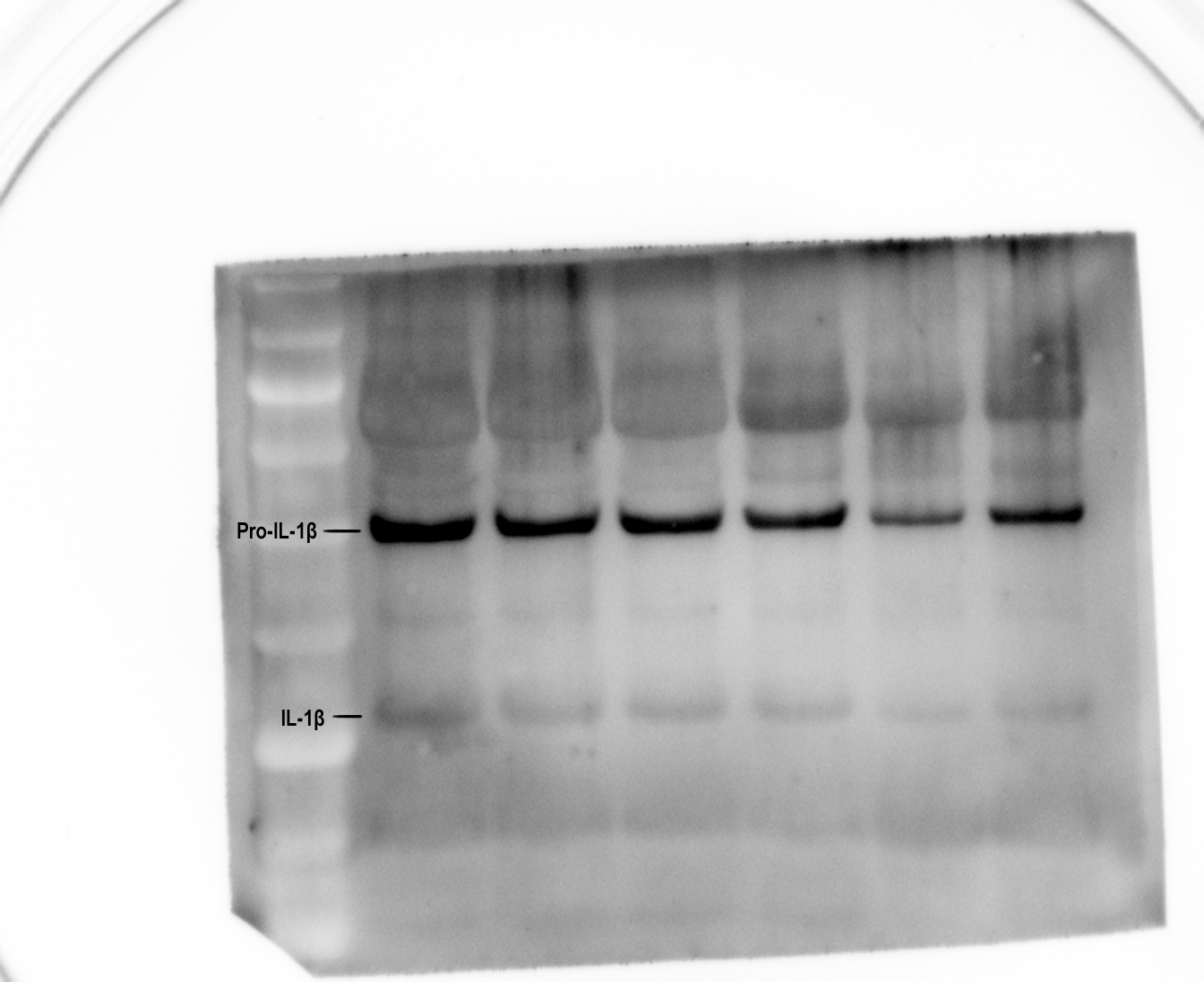

Supplement: Figure 7—source data 2. [file elife-98714-fig7-data2.zip › FIG7 source data-foot tissue/IL-1a┬/IL-1a┬.Tif]

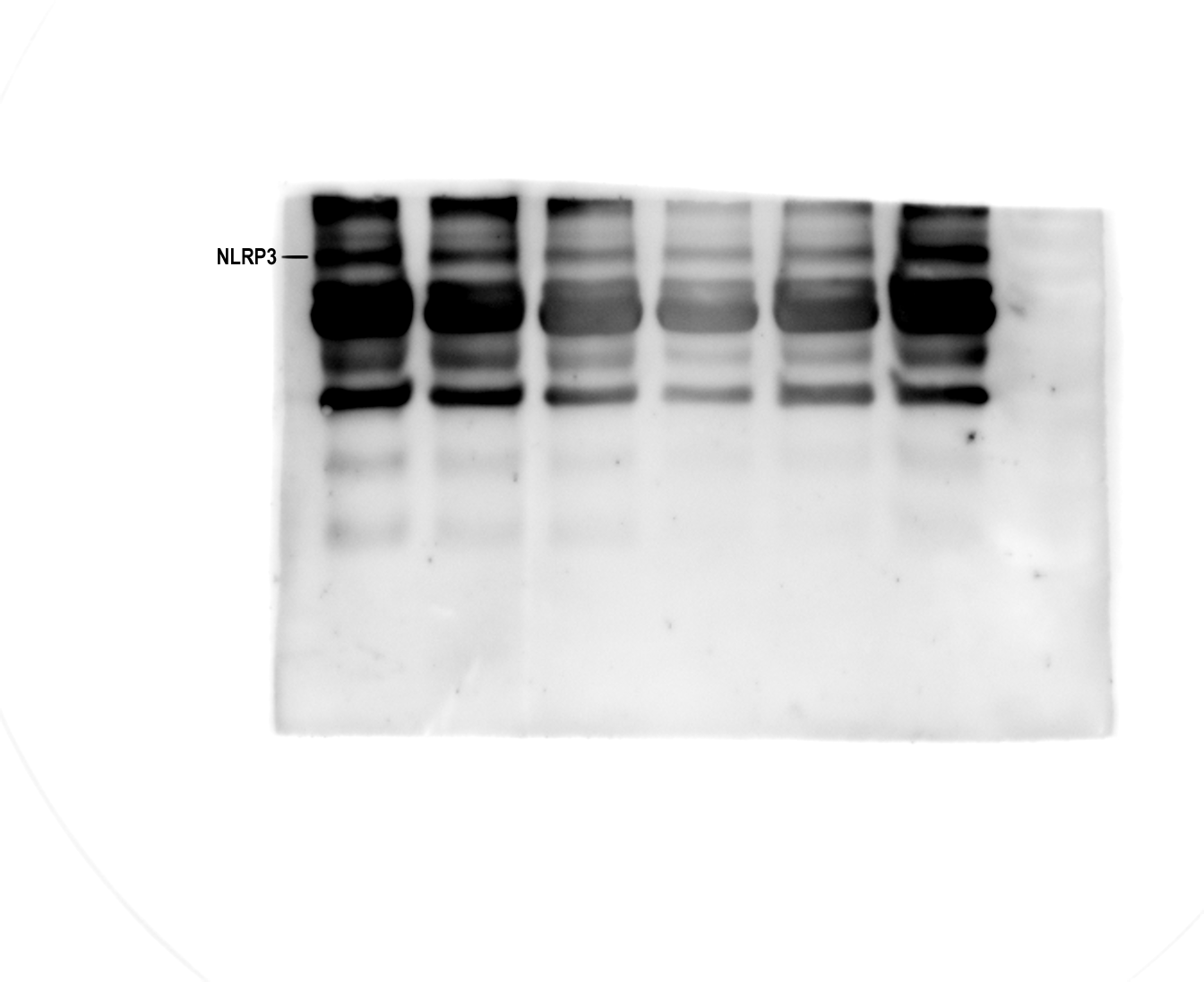

Supplement: Figure 7—source data 2. [file elife-98714-fig7-data2.zip › FIG7 source data-foot tissue/NLRP3/NLRP3.Tif]

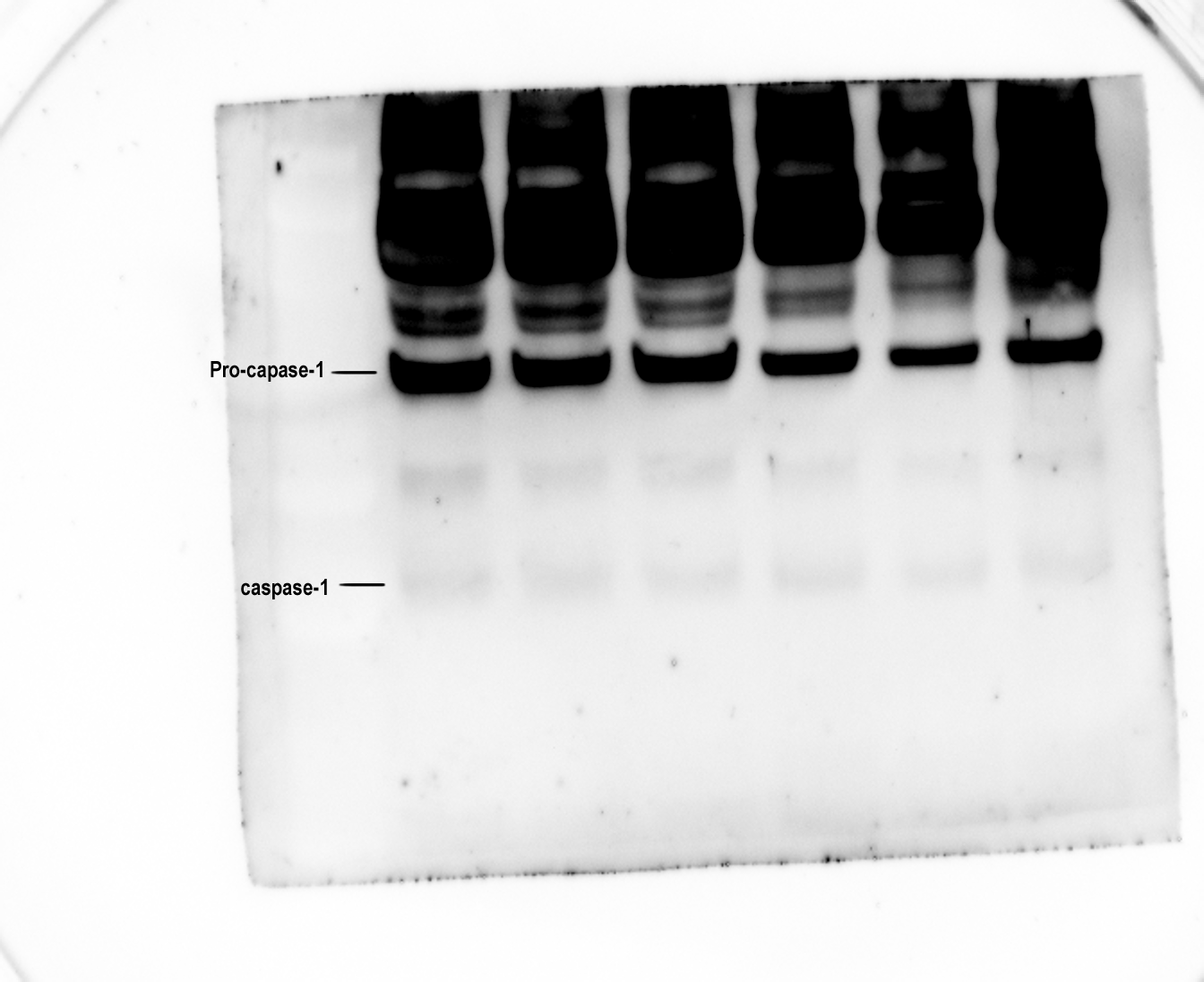

Supplement: Figure 7—figure supplement 1—source data 2. [file elife-98714-fig7-figsupp1-data2.zip › FIG7 figure supplements 1 source data-cell/CASPASE-1/caspase-1.Tif]

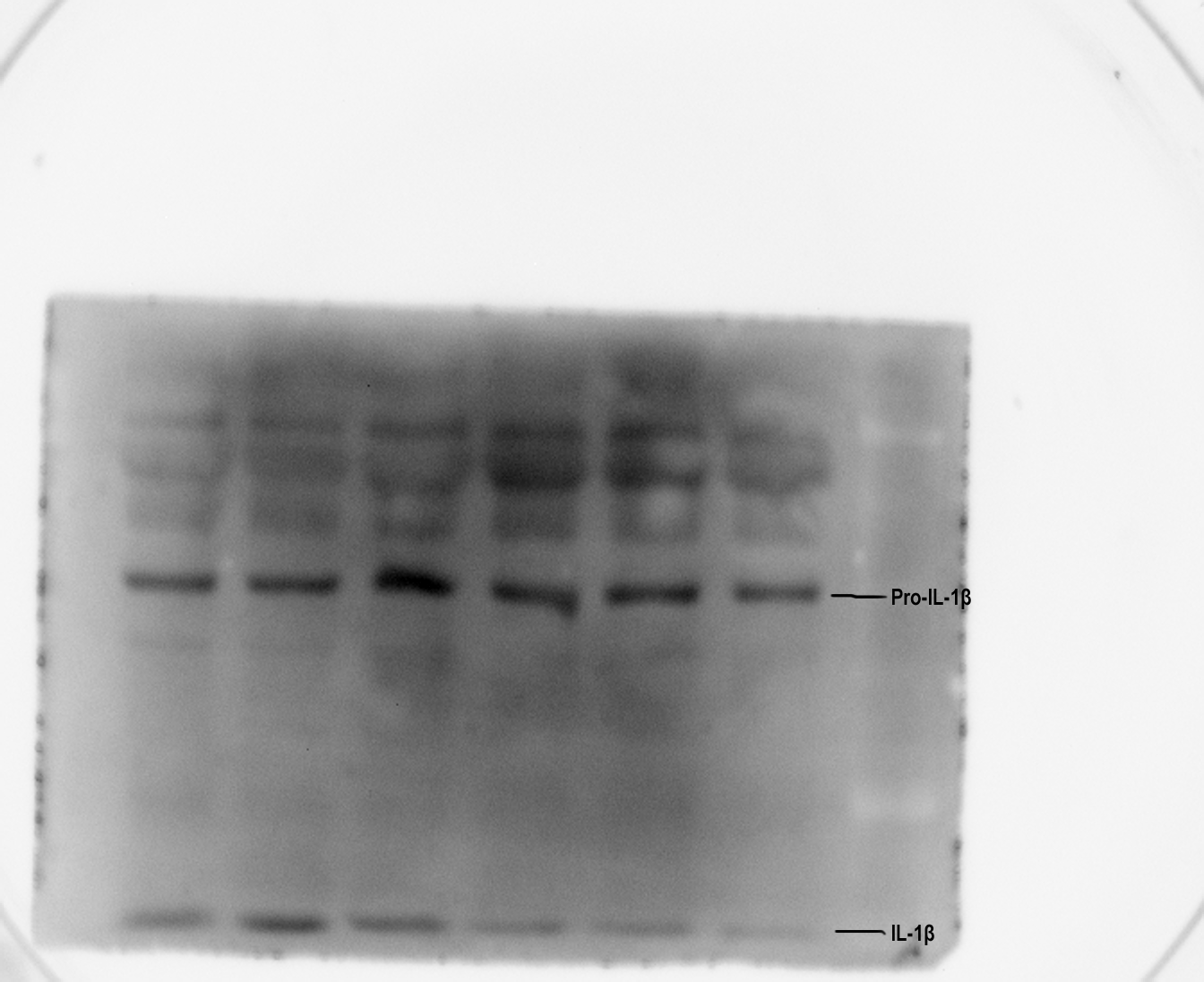

Supplement: Figure 7—figure supplement 1—source data 2. [file elife-98714-fig7-figsupp1-data2.zip › FIG7 figure supplements 1 source data-cell/IL-1a┬/IL-1a┬.Tif]

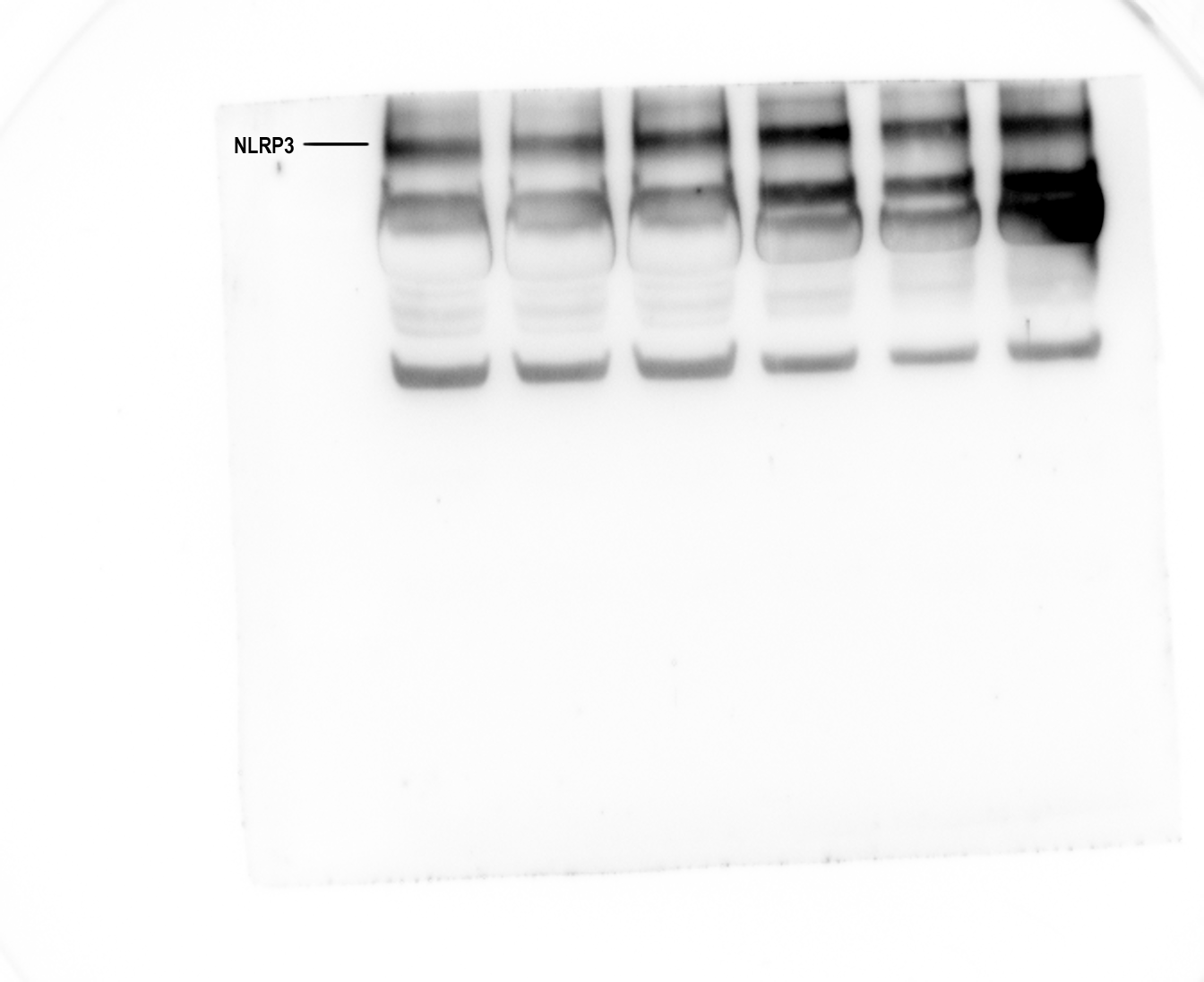

Supplement: Figure 7—figure supplement 1—source data 2. [file elife-98714-fig7-figsupp1-data2.zip › FIG7 figure supplements 1 source data-cell/NLRP3/NLRP3.Tif]

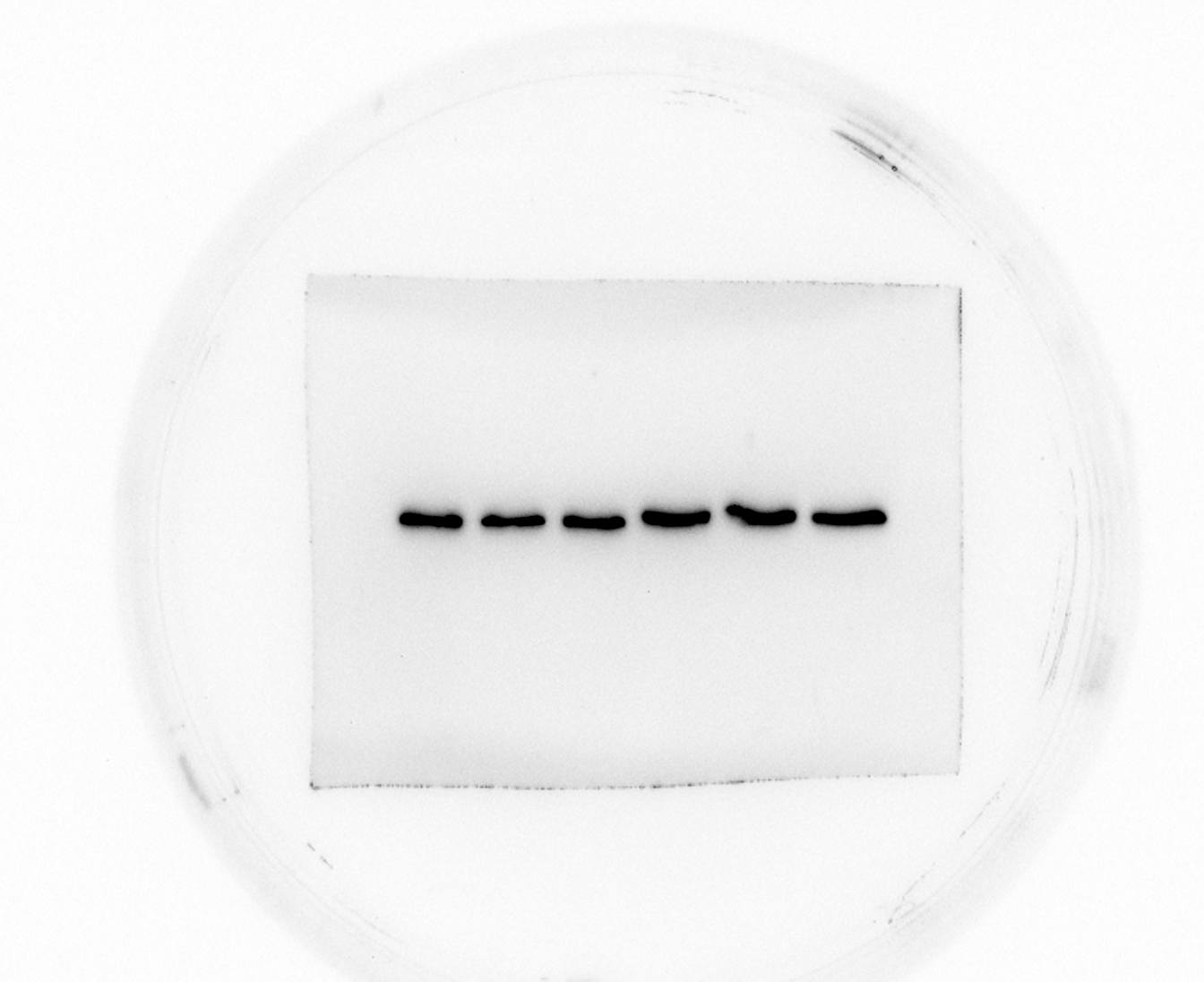

Supplement: Figure 7—figure supplement 1—source data 2. [file elife-98714-fig7-figsupp1-data2.zip › FIG7 figure supplements 1 source data-cell/a┬-actin/a┬-actin rerun.Tif]
