# Supplementary material for: Microbiota from young mice counteracts susceptibility to age-related gout through modulating butyric acid levels in aged mice
Source: eLife. 2025 Feb 5;13:RP98714. doi: 10.7554/eLife.98714 (PMC11798573; doi:10.7554/eLife.98714)
Supplement: Figure 3—figure supplement 1—source data 1. [file elife-98714-fig3-figsupp1-data1.pdf]

FIG3 figure supplements 1 source data

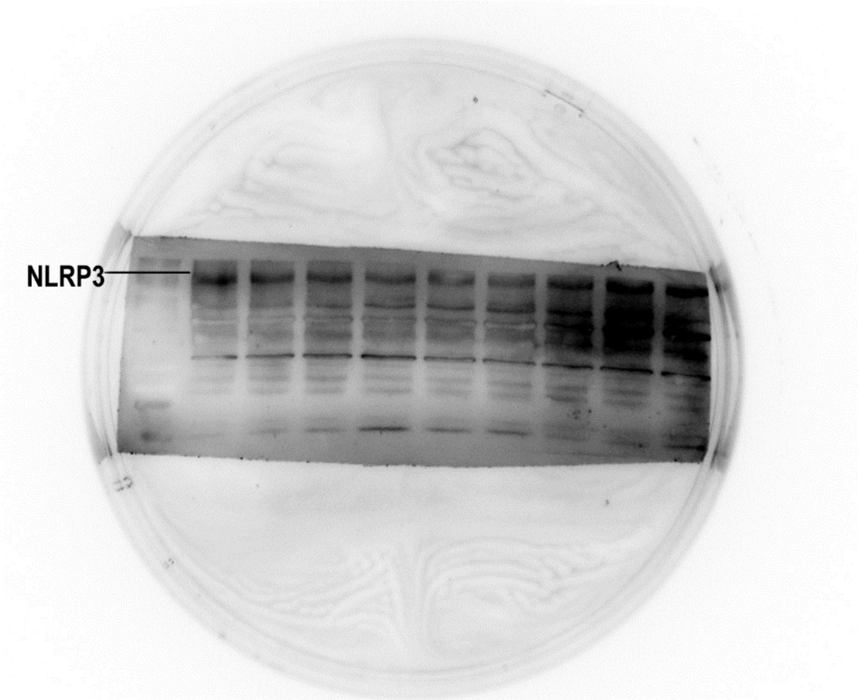

Representative western blot images (Young+PBS, Young+Old and Young+Aged) of cell NLRP3 protein (n=3).

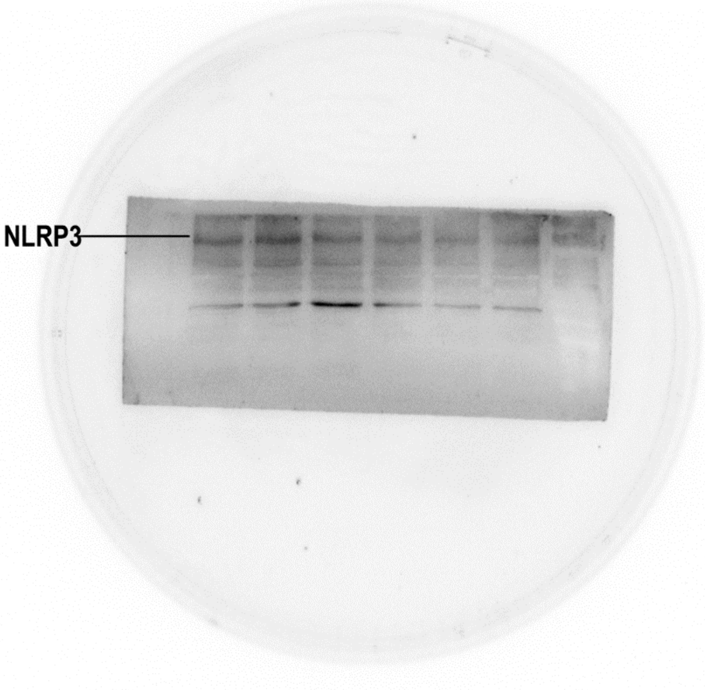

Representative western blot images (Old+PBS and Old+Young) of cell NLRP3 protein (n=3).

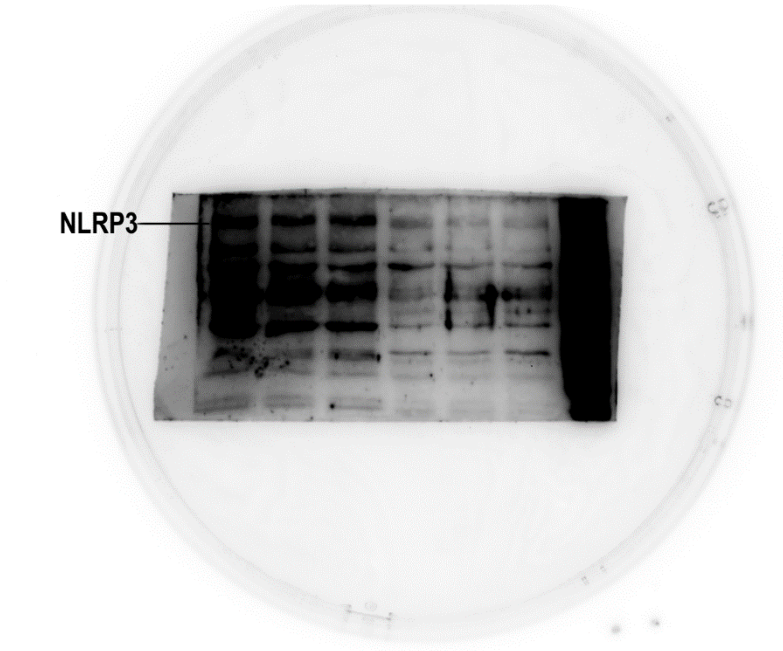

Representative western blot images (Aged+PBS and Aged+Young) of cell NLRP3 protein（n=3）

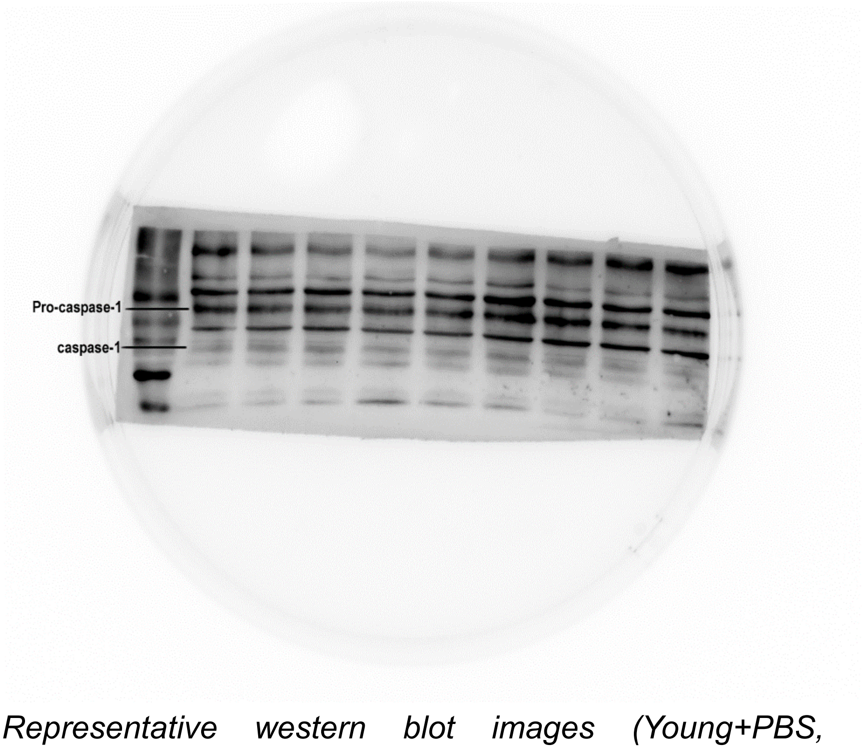

Representative western blot images (Young+PBS, Young+Old and Young+Aged) of cell CASPASE protein (n=3).

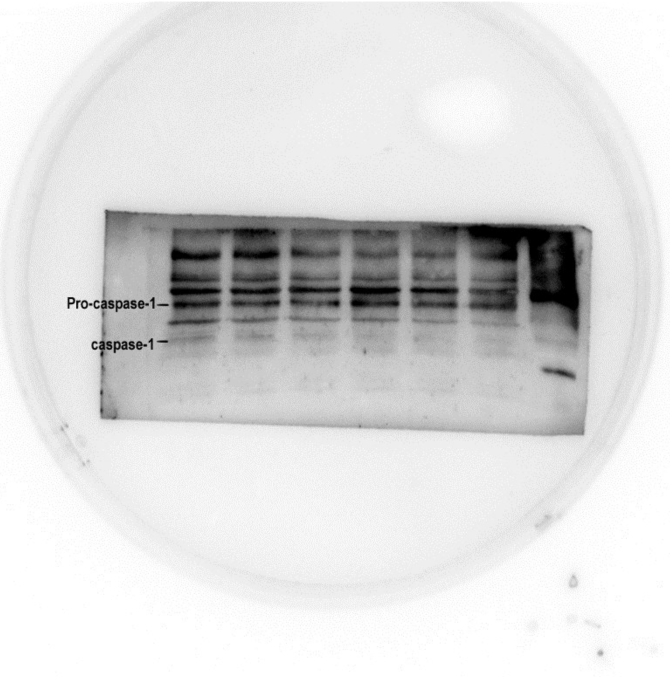

Representative western blot images (Old+PBS and Old+Young) of cell CASPASE protein (n=3).

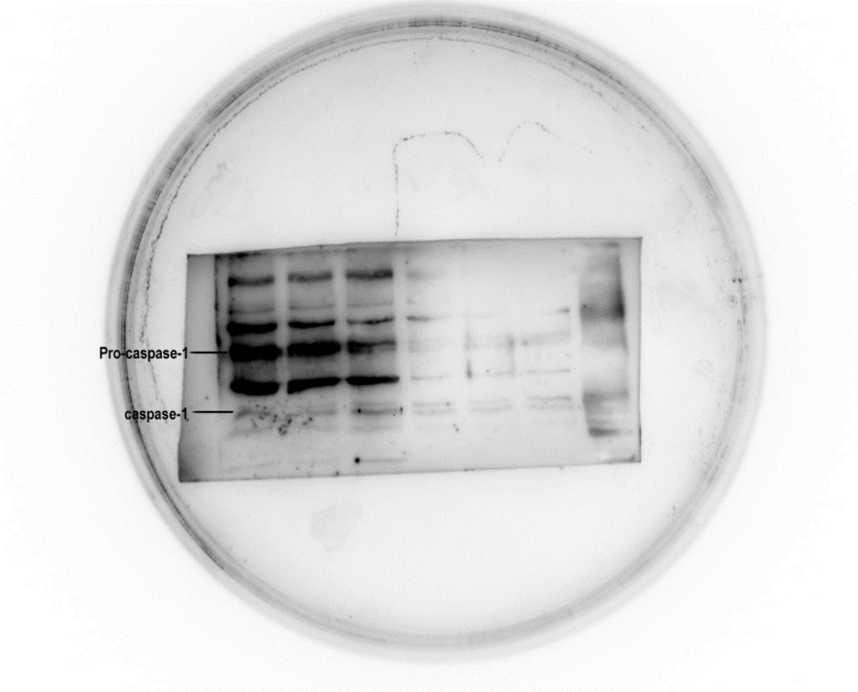

Representative western blot images (Aged+PBS and Aged+Young) of cell CASPASE protein（n=3）

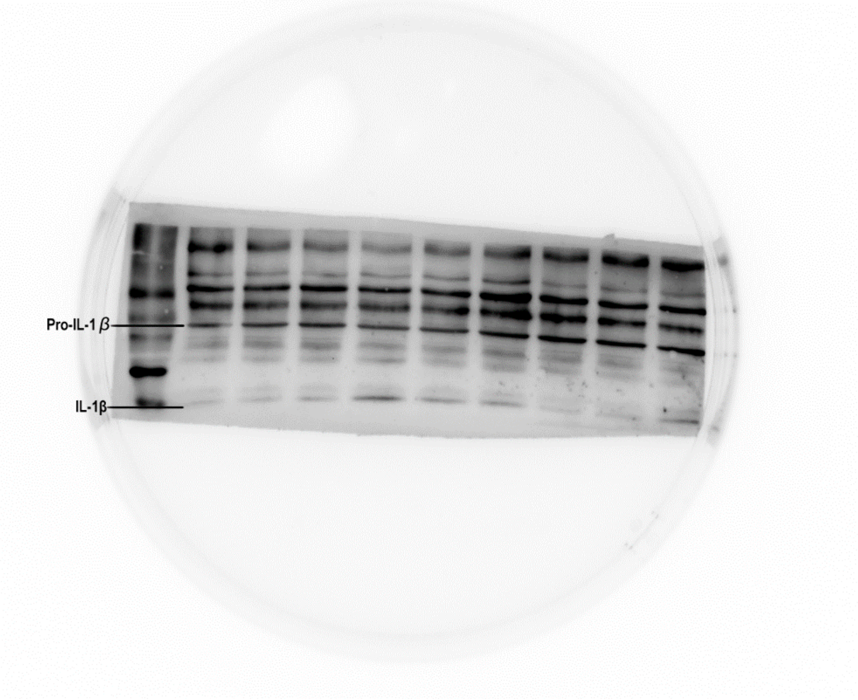

Representative western blot images (Young+PBS, Young+Old and Young+Aged) of cell IL-1β protein (n=3).

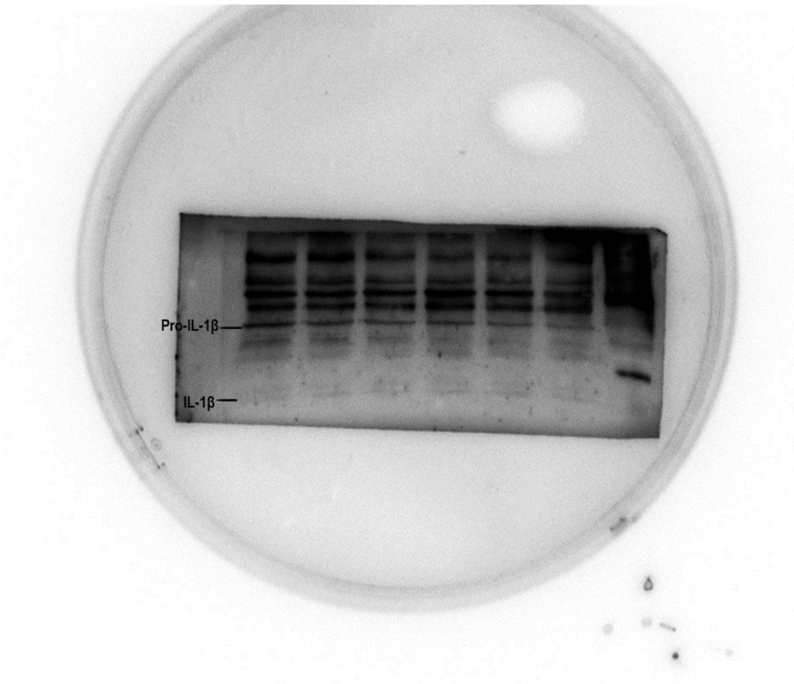

Representative western blot images (Old+PBS and Old+Young) of cell IL-1β protein (n=3).

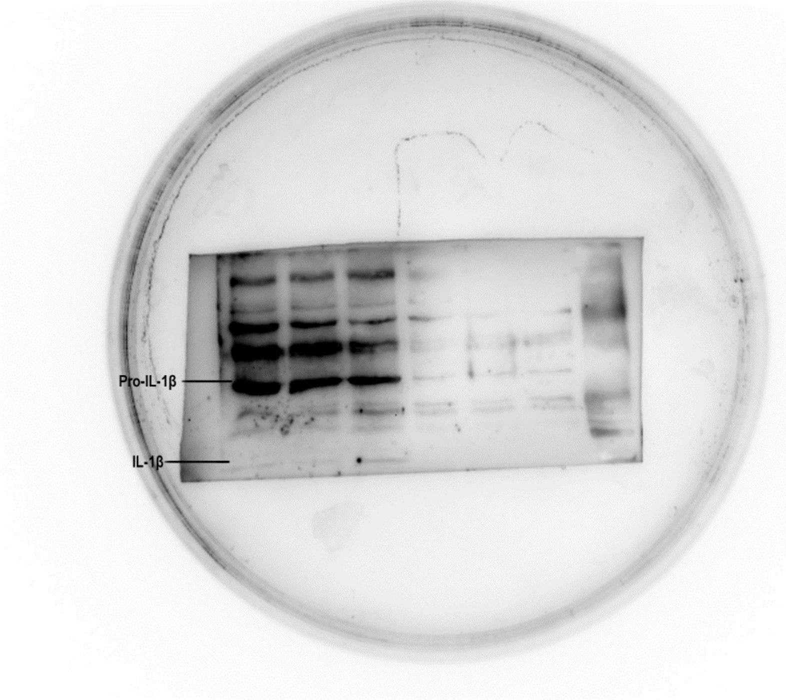

Representative western blot images (Aged+PBS and Aged+Young) of cell IL-1β protein（n=3）

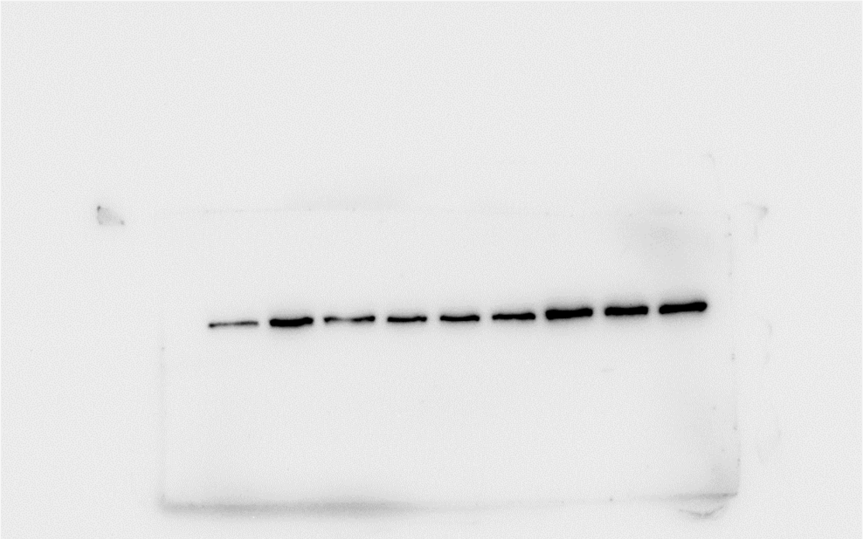

Representative western blot images (Young+PBS, Young+Old and Young+Aged) of foot tissue β-actin protein (n=3).

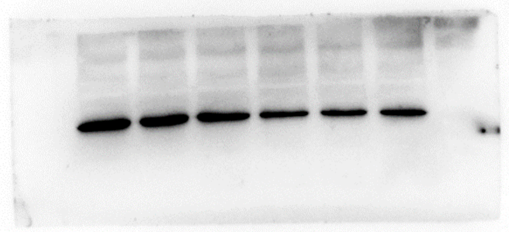

Representative western blot images (Old+PBS and Old+Young) of foot tissue β-actin protein (n=3).

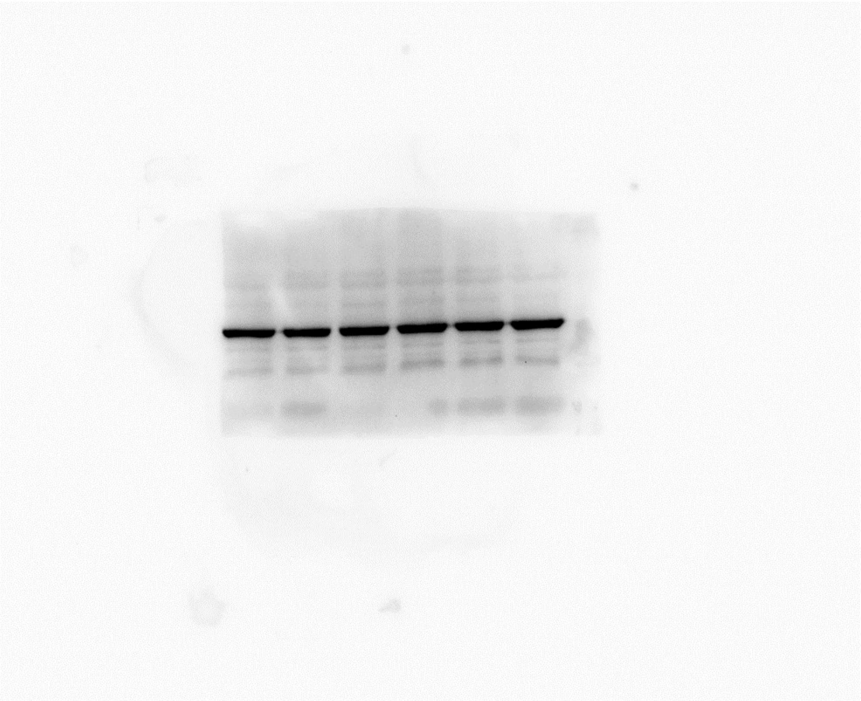

Representative western blot images (Aged+PBS and Aged+Young) of foot tissue β-actin protein （n=3）
