# Supplementary material for: Microbiota from young mice counteracts susceptibility to age-related gout through modulating butyric acid levels in aged mice
Source: eLife. 2025 Feb 5;13:RP98714. doi: 10.7554/eLife.98714 (PMC11798573; doi:10.7554/eLife.98714)
Supplement: Figure 7—source data 1. [file elife-98714-fig7-data1.pdf]

FIG7 source data

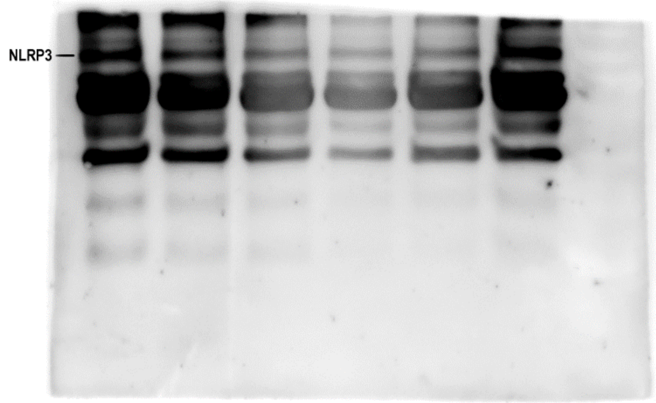

Representative western blot images (Control and Butyrate) of foot tissue NLRP3 protein (n=3).

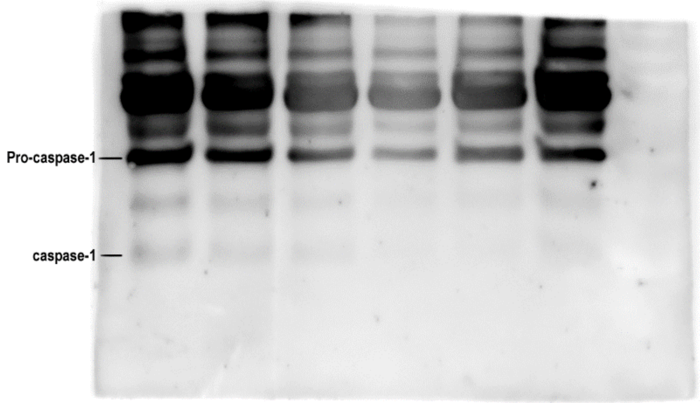

Representative western blot images (Control and Butyrate) of foot tissue CASPASE protein (n=3).

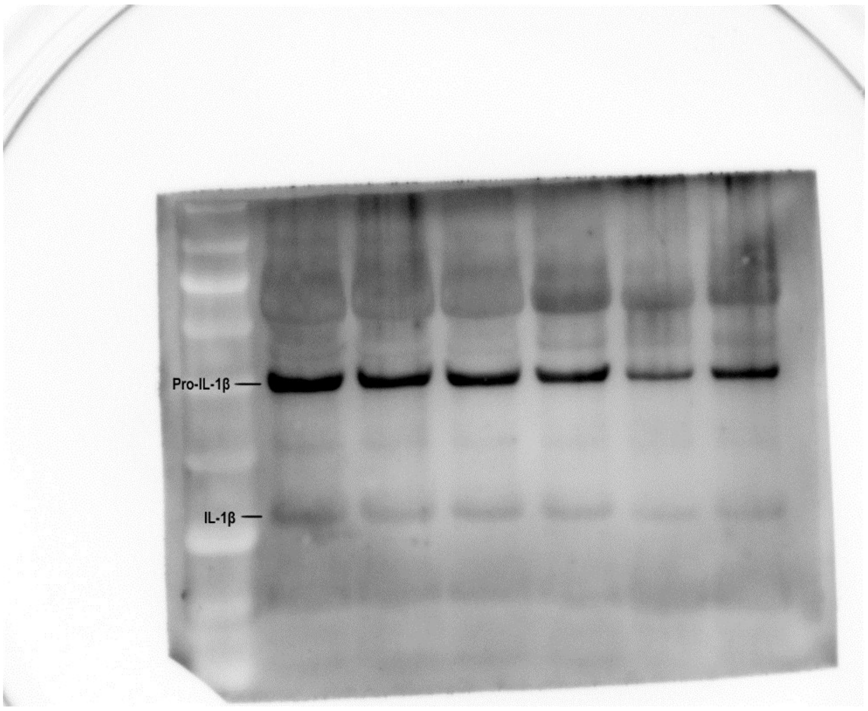

Representative western blot images (Control and Butyrate) of foot tissue IL-1β protein (n=3).

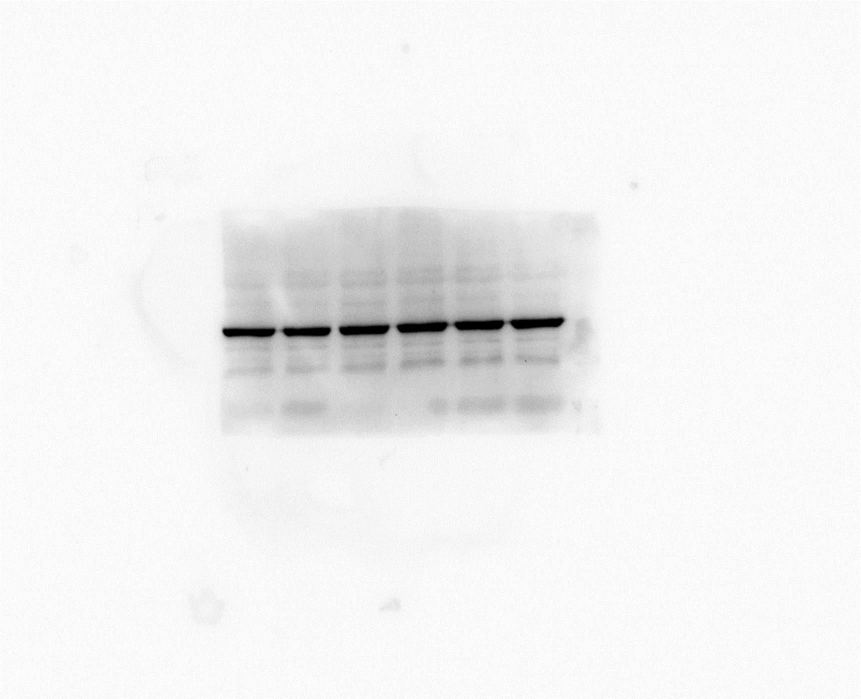
Representative western blot images (Control and Butyrate) of foot tissue β-actin protein (n=3).
