# Supplementary material for: Microbiota from young mice counteracts susceptibility to age-related gout through modulating butyric acid levels in aged mice
Source: eLife. 2025 Feb 5;13:RP98714. doi: 10.7554/eLife.98714 (PMC11798573; doi:10.7554/eLife.98714)
Supplement: Supplementary file 1. [file elife-98714-supp1.docx]

| Oligonucleotides |  |  |
| --- | --- | --- |
| Gene | Primer | Sequence(5′ to 3′) |
| *Havcr1*（KIM-1） | sense | TGGTTGCCTTCCGTGTCTCT |
|  | antisense | TCAGCTCGGGAATGCACAA |
| *Slc22a12* (URAT1) | sense | GGCTCATCACAAAGACCCCA |
|  | antisense | CAGCATGTTCTGGGTGGTGA |
| *Slc22a6* (OAT1) | sense | GCAGCCTATGCACCCAACTA |
|  | antisense | ACAGCATAGGCGATACCAGC |
| *Slc22a8* (OAT3) | sense | GGGCTCGAGTGGGAAGTATG |
|  | antisense | CTGGTAAGGGCCGATTGAGG |
| *Slc2a9* (GLUT9) | sense | GAAGGGTGTTCCTGGCTACC |
|  | antisense | TGAACACGGACCCAAACCAG |
| *Abcg2* (ABCG2) | sense | TTGGACTCAAGCACAGCGAATG |
|  | antisense | TCCGCAGGGTTGTTGTAGGG |
| *Tjp1* (ZO-1) | sense | TTTCAGAGTGGGGAAACCTCC |
|  | antisense | CACTCTTCCTTAGCTGCTGAAC |
| *F11r* (JAMA) | sense | TTGGCGTCTGGTTTGCCTAT |
|  | antisense | CACTTCGAGTACTGGGCTGG |
| *Ocln* (Occludin) | sense | ATGCTCTCTCAGCCAGCGTA |
|  | antisense | CCACACAGGCAAATATGGCG |
| *GAPDH* | sense | AGGTCGGTGTGAACGGATTTG |
|  | antisense | GGGGTCGTTGATGGCAACA |
