# Supplementary material for: Microbiota from young mice counteracts susceptibility to age-related gout through modulating butyric acid levels in aged mice
Source: eLife. 2025 Feb 5;13:RP98714. doi: 10.7554/eLife.98714 (PMC11798573; doi:10.7554/eLife.98714)
Supplement: Supplementary file 2. [file elife-98714-supp2.docx]

Table 2 Enhancement of Butanoate metabolism contributes to the prevention of elderly gout.

| Table 2a Young+PBS VS Old+PBS | | | | | | | | |
| --- | --- | --- | --- | --- | --- | --- | --- | --- |
| Taxa | avg(Young+PBS) | sd(Young+PBS) | avg(Old+PBS) | sd(Old+PBS) | p.value | q.values | interval lower | interval upper |
| Genetic Information Processing;Translation;Transfer_RNA_biogenesis | 0.025388 | 0.000319 | 0.026282 | 0.000752 | 0.032527 | 0.095267 | -0.00169 | -1E-04 |
| Metabolism;Amino acid metabolism;Amino_acid_related_enzymes | 0.018844 | 0.000148 | 0.019137 | 0.000258 | 0.042118 | 0.102535 | -0.00057 | -1.3E-05 |
| Genetic Information Processing;Translation;Ribosome | 0.015723 | 0.000269 | 0.016458 | 0.000594 | 0.028278 | 0.090633 | -0.00137 | -0.0001 |
| Genetic Information Processing;Translation;Aminoacyl-tRNA_biosynthesis | 0.015499 | 0.000262 | 0.0164 | 0.000581 | 0.0106 | 0.060087 | -0.00152 | -0.00029 |
| Cellular Processes;Transport and catabolism;Exosome | 0.015496 | 0.000151 | 0.014721 | 0.000475 | 0.008904 | 0.057414 | 0.000277 | 0.001273 |
| Genetic Information Processing;Replication and repair;DNA_replication_proteins | 0.014807 | 0.000271 | 0.015186 | 0.000287 | 0.040656 | 0.101038 | -0.00074 | -2E-05 |
| Genetic Information Processing;Replication and repair;Chromosome_and_associated_proteins | 0.0131 | 0.000238 | 0.013673 | 0.000459 | 0.028126 | 0.090633 | -0.00107 | -8E-05 |
| Metabolism;Amino acid metabolism;Alanine,_aspartate_and_glutamate_metabolism | 0.011744 | 0.000114 | 0.011268 | 0.000326 | 0.014186 | 0.066361 | 0.000134 | 0.000819 |
| Metabolism;Amino acid metabolism;Cysteine_and_methionine_metabolism | 0.009602 | 7.4E-05 | 0.009046 | 0.000372 | 0.013822 | 0.065951 | 0.000166 | 0.000946 |
| Genetic Information Processing;Replication and repair;Nucleotide_excision_repair | 0.008149 | 0.000239 | 0.008575 | 0.000252 | 0.013177 | 0.065517 | -0.00074 | -0.00011 |
| Genetic Information Processing;Replication and repair;DNA_replication | 0.007972 | 0.000183 | 0.008423 | 0.000317 | 0.016667 | 0.072413 | -0.0008 | -0.00011 |
| Genetic Information Processing;Translation;Messenger_RNA_Biogenesis | 0.007849 | 5.8E-05 | 0.008027 | 0.000156 | 0.037877 | 0.10003 | -0.00034 | -1.4E-05 |
| Metabolism;Amino acid metabolism;Glycine,_serine_and_threonine_metabolism | 0.007893 | 0.000123 | 0.007344 | 0.000264 | 0.00238 | 0.042225 | 0.000268 | 0.00083 |
| Metabolism;Carbohydrate metabolism;Pentose_phosphate_pathway | 0.007552 | 0.000179 | 0.007998 | 0.000372 | 0.032208 | 0.095267 | -0.00084 | -5E-05 |
| Metabolism;Carbohydrate metabolism;Butanoate_metabolism | 0.007496 | 0.000432 | 0.006856 | 0.000234 | 0.013454 | 0.065517 | 0.000174 | 0.001105 |
| Metabolism;Carbohydrate metabolism;Glyoxylate_and_dicarboxylate_metabolism | 0.007261 | 0.000272 | 0.006593 | 0.00058 | 0.037471 | 0.10003 | 5.11E-05 | 0.001284 |
| Metabolism;Amino acid metabolism;Lysine_biosynthesis | 0.006277 | 0.000198 | 0.006577 | 0.000222 | 0.033542 | 0.095267 | -0.00057 | -2.9E-05 |
| Metabolism;Amino acid metabolism;Arginine_biosynthesis | 0.005337 | 0.000206 | 0.004818 | 0.000269 | 0.004248 | 0.042225 | 0.000208 | 0.00083 |
| Metabolism;Lipid metabolism;Lipid_biosynthesis_proteins | 0.004748 | 0.000253 | 0.004195 | 0.000254 | 0.003621 | 0.042225 | 0.000227 | 0.00088 |
| Metabolism;Metabolism of terpenoids and polyketides;Terpenoid_backbone_biosynthesis | 0.004268 | 0.000118 | 0.004511 | 0.000132 | 0.007384 | 0.057414 | -0.0004 | -8.1E-05 |
| Metabolism;Lipid metabolism;Glycerophospholipid_metabolism | 0.004116 | 0.00012 | 0.00449 | 0.00018 | 0.002372 | 0.042225 | -0.00058 | -0.00017 |
| Metabolism;Energy metabolism;Nitrogen_metabolism | 0.004843 | 0.000271 | 0.004057 | 0.000366 | 0.002089 | 0.042225 | 0.000368 | 0.001206 |
| Metabolism;Metabolism of cofactors and vitamins;One_carbon_pool_by_folate | 0.004295 | 4.61E-05 | 0.003931 | 0.000257 | 0.017267 | 0.073561 | 9.46E-05 | 0.000634 |
| Genetic Information Processing;Replication and repair;Base_excision_repair | 0.003915 | 6.37E-05 | 0.004159 | 0.000206 | 0.033021 | 0.095267 | -0.00046 | -2.7E-05 |
| Metabolism;Metabolism of cofactors and vitamins;Pantothenate_and_CoA_biosynthesis | 0.004248 | 0.000113 | 0.003948 | 0.000163 | 0.00495 | 0.045425 | 0.000117 | 0.000483 |
| Metabolism;Glycan biosynthesis and metabolism;Glycosyltransferases | 0.003709 | 0.000154 | 0.003904 | 0.000119 | 0.035217 | 0.098192 | -0.00037 | -1.7E-05 |
| Metabolism;Amino acid metabolism;Valine,_leucine_and_isoleucine_biosynthesis | 0.003878 | 0.000305 | 0.00317 | 0.000297 | 0.002248 | 0.042225 | 0.000321 | 0.001096 |
| Genetic Information Processing;Transcription;RNA_polymerase | 0.003305 | 7E-05 | 0.003533 | 0.000182 | 0.026747 | 0.090633 | -0.00042 | -3.6E-05 |
| Metabolism;Carbohydrate metabolism;Pentose_and_glucuronate_interconversions | 0.003491 | 7.67E-05 | 0.002991 | 0.000471 | 0.048036 | 0.108091 | 6.23E-06 | 0.000993 |
| Metabolism;Glycan biosynthesis and metabolism;Other_glycan_degradation | 0.002999 | 0.000399 | 0.002122 | 0.000652 | 0.022059 | 0.080739 | 0.000162 | 0.001592 |
| Metabolism;Energy metabolism;Photosynthesis_proteins | 0.00274 | 0.000139 | 0.00297 | 8.72E-05 | 0.008279 | 0.057414 | -0.00038 | -7.7E-05 |
| Metabolism;Energy metabolism;Photosynthesis | 0.002731 | 0.000139 | 0.002958 | 8.61E-05 | 0.008761 | 0.057414 | -0.00038 | -7.4E-05 |
| Metabolism;Amino acid metabolism;Valine,_leucine_and_isoleucine_degradation | 0.00267 | 0.000206 | 0.002337 | 0.00011 | 0.008698 | 0.057414 | 0.000112 | 0.000556 |
| Cellular Processes;Cell motility;Cytoskeleton_proteins | 0.002762 | 6.51E-05 | 0.002926 | 6.67E-05 | 0.001549 | 0.042225 | -0.00025 | -7.9E-05 |
| Cellular Processes;Cellular community - prokaryotes;Biofilm_formation-Escherichia_coli | 0.002944 | 0.00017 | 0.002446 | 0.000315 | 0.009901 | 0.060087 | 0.000158 | 0.000837 |
| Metabolism;Metabolism of other amino acids;Glutathione_metabolism | 0.002308 | 0.000124 | 0.002655 | 0.000202 | 0.006697 | 0.057064 | -0.00057 | -0.00013 |
| Metabolism;Amino acid metabolism;Histidine_metabolism | 0.002726 | 0.000184 | 0.002115 | 0.000282 | 0.00178 | 0.042225 | 0.000299 | 0.000924 |
| Metabolism;Xenobiotics biodegradation and metabolism;Drug_metabolism-other_enzymes | 0.002595 | 4.74E-05 | 0.002414 | 0.000157 | 0.035973 | 0.098192 | 1.66E-05 | 0.000346 |
| Metabolism;Metabolism of other amino acids;Cyanoamino_acid_metabolism | 0.002675 | 7.72E-05 | 0.002356 | 0.000246 | 0.023199 | 0.082607 | 6.12E-05 | 0.000577 |
| Unclassified;Cellular processes and signaling;Cell_growth | 0.002913 | 0.000282 | 0.002442 | 0.000367 | 0.033195 | 0.095267 | 4.65E-05 | 0.000897 |
| Cellular Processes;Cell growth and death;Necroptosis | 0.002668 | 0.000129 | 0.002208 | 0.000181 | 0.00066 | 0.042225 | 0.000255 | 0.000665 |
| Genetic Information Processing;Folding, sorting and degradation;Sulfur_relay_system | 0.002033 | 6.24E-05 | 0.002222 | 0.000115 | 0.008311 | 0.057414 | -0.00031 | -6.4E-05 |
| Metabolism;Biosynthesis of other secondary metabolites;Monobactam_biosynthesis | 0.002121 | 3.47E-05 | 0.001984 | 8.96E-05 | 0.011592 | 0.061459 | 4.23E-05 | 0.000231 |
| Metabolism;Carbohydrate metabolism;C5-Branched_dibasic_acid_metabolism | 0.002032 | 0.000102 | 0.001717 | 0.000241 | 0.022336 | 0.080739 | 6.07E-05 | 0.00057 |
| Cellular Processes;Cellular community - prokaryotes;Biofilm_formation-Vibrio_cholerae | 0.001887 | 0.0001 | 0.001714 | 0.00012 | 0.022317 | 0.080739 | 3.04E-05 | 0.000315 |
| Metabolism;Amino acid metabolism;Phenylalanine_metabolism | 0.001845 | 0.000192 | 0.00157 | 9.34E-05 | 0.015359 | 0.070468 | 7.01E-05 | 0.000479 |
| Human Diseases;Endocrine and metabolic diseases;Insulin_resistance | 0.001786 | 0.000103 | 0.001495 | 0.000116 | 0.001045 | 0.042225 | 0.000149 | 0.000433 |
| Metabolism;Metabolism of terpenoids and polyketides;Prenyltransferases | 0.001408 | 5.91E-05 | 0.001516 | 8.12E-05 | 0.026094 | 0.090224 | -0.0002 | -1.6E-05 |
| Metabolism;Metabolism of other amino acids;D-Glutamine_and_D-glutamate_metabolism | 0.001416 | 4.8E-05 | 0.001524 | 5.98E-05 | 0.006665 | 0.057064 | -0.00018 | -3.8E-05 |
| Environmental Information Processing;Signal transduction;HIF-1_signaling_pathway | 0.001241 | 3.17E-05 | 0.001343 | 9.34E-05 | 0.043249 | 0.103127 | -0.0002 | -4.3E-06 |
| Metabolism;Glycan biosynthesis and metabolism;Glycosphingolipid_biosynthesis-globo_and_isoglobo_series | 0.001423 | 0.000143 | 0.001106 | 0.000281 | 0.041764 | 0.102535 | 1.54E-05 | 0.000618 |
| Metabolism;Biosynthesis of other secondary metabolites;Phenylpropanoid_biosynthesis | 0.001572 | 6.81E-05 | 0.001159 | 0.000289 | 0.016311 | 0.072413 | 0.00011 | 0.000715 |
| Organismal Systems;Nervous system;GABAergic_synapse | 0.001346 | 3.61E-05 | 0.001224 | 7.85E-05 | 0.010521 | 0.060087 | 3.86E-05 | 0.000205 |
| Metabolism;Metabolism of cofactors and vitamins;Vitamin_B6_metabolism | 0.00127 | 5.5E-05 | 0.001084 | 0.000119 | 0.010047 | 0.060087 | 6E-05 | 0.000312 |
| Organismal Systems;Nervous system;Glutamatergic_synapse | 0.001287 | 3.41E-05 | 0.001179 | 7.72E-05 | 0.016694 | 0.072413 | 2.65E-05 | 0.00019 |
| Metabolism;Enzyme families;Protein_phosphatase_and_associated_proteins | 0.001301 | 7.07E-05 | 0.001114 | 0.00012 | 0.010832 | 0.060087 | 5.64E-05 | 0.000319 |
| Unclassified;Metabolism;Carbohydrate_metabolism | 0.001315 | 0.000147 | 0.001019 | 0.000118 | 0.00351 | 0.042225 | 0.000123 | 0.000468 |
| Organismal Systems;Endocrine system;Insulin_signaling_pathway | 0.001273 | 8.54E-05 | 0.00102 | 0.000129 | 0.003262 | 0.042225 | 0.00011 | 0.000397 |
| Organismal Systems;Endocrine system;PPAR_signaling_pathway | 0.001209 | 6.31E-05 | 0.000931 | 0.000165 | 0.007484 | 0.057414 | 0.000104 | 0.000452 |
| Metabolism;Metabolism of other amino acids;beta-Alanine_metabolism | 0.001119 | 0.000151 | 0.000946 | 6.77E-05 | 0.038154 | 0.10003 | 1.25E-05 | 0.000333 |
| Unclassified;Viral protein family;Viral_proteins | 0.00087 | 4.86E-05 | 0.000995 | 5.84E-05 | 0.002547 | 0.042225 | -0.00019 | -5.6E-05 |
| Metabolism;Glycan biosynthesis and metabolism;Glycosaminoglycan_degradation | 0.000925 | 0.000151 | 0.000591 | 0.000218 | 0.013149 | 0.065517 | 8.89E-05 | 0.000579 |
| Metabolism;Biosynthesis of other secondary metabolites;Novobiocin_biosynthesis | 0.001016 | 5.43E-05 | 0.00082 | 0.000101 | 0.003339 | 0.042225 | 8.73E-05 | 0.000305 |
| Metabolism;Biosynthesis of other secondary metabolites;Tropane,_piperidine_and_pyridine_alkaloid_biosynthesis | 0.001043 | 6.63E-05 | 0.000805 | 7.65E-05 | 0.000202 | 0.042225 | 0.000145 | 0.00033 |
| Cellular Processes;Transport and catabolism;Lysosome | 0.00084 | 0.0002 | 0.000516 | 0.000241 | 0.030551 | 0.093446 | 3.74E-05 | 0.00061 |
| Metabolism;Carbohydrate metabolism;Ascorbate_and_aldarate_metabolism | 0.000764 | 6.21E-05 | 0.000622 | 8.97E-05 | 0.011082 | 0.060087 | 4.14E-05 | 0.000243 |
| Metabolism;Biosynthesis of other secondary metabolites;Acarbose_and_validamycin_biosynthesis | 0.000751 | 2.85E-05 | 0.000653 | 9.16E-05 | 0.045896 | 0.10735 | 2.48E-06 | 0.000194 |
| Metabolism;Lipid metabolism;Synthesis_and_degradation_of_ketone_bodies | 0.000626 | 3.57E-05 | 0.000706 | 7.54E-05 | 0.049832 | 0.108091 | -0.00016 | -7.7E-08 |
| Environmental Information Processing;Signal transduction;AMPK_signaling_pathway | 0.000694 | 4.69E-05 | 0.000623 | 3.94E-05 | 0.018262 | 0.076435 | 1.49E-05 | 0.000127 |
| Unclassified;Metabolism;Glycan_biosynthesis_and_metabolism | 0.000642 | 4.08E-05 | 0.000512 | 6.35E-05 | 0.002595 | 0.042225 | 5.91E-05 | 0.0002 |
| Environmental Information Processing;Signal transduction;Phosphatidylinositol_signaling_system | 0.000471 | 3.16E-05 | 0.000509 | 1.46E-05 | 0.030116 | 0.09331 | -7.2E-05 | -4.9E-06 |
| Cellular Processes;Cell growth and death;Ferroptosis | 0.000572 | 3.2E-05 | 0.00033 | 0.000121 | 0.003812 | 0.042225 | 0.000114 | 0.000368 |
| Genetic Information Processing;Folding, sorting and degradation;Proteasome | 0.000566 | 5.42E-05 | 0.000444 | 0.000112 | 0.047251 | 0.108059 | 1.93E-06 | 0.000241 |
| Organismal Systems;Endocrine system;Adipocytokine_signaling_pathway | 0.000557 | 4.04E-05 | 0.000303 | 0.00013 | 0.0038 | 0.042225 | 0.000118 | 0.00039 |
| Metabolism;Biosynthesis of other secondary metabolites;Isoquinoline_alkaloid_biosynthesis | 0.000477 | 1.95E-05 | 0.000386 | 6.85E-05 | 0.020656 | 0.078274 | 1.99E-05 | 0.000163 |
| Metabolism;Lipid metabolism;Secondary_bile_acid_biosynthesis | 0.000335 | 2.13E-05 | 0.000417 | 6.43E-05 | 0.025372 | 0.089018 | -0.00015 | -1.4E-05 |
| Metabolism;Biosynthesis of other secondary metabolites;Glucosinolate_biosynthesis | 0.000341 | 3.28E-05 | 0.000265 | 2.64E-05 | 0.001454 | 0.042225 | 3.73E-05 | 0.000114 |
| Metabolism;Glycan biosynthesis and metabolism;Glycosylphosphatidylinositol_(GPI)-anchored_proteins | 0.000296 | 4.79E-05 | 0.000188 | 5.55E-05 | 0.004804 | 0.045425 | 4.17E-05 | 0.000175 |
| Metabolism;Glycan biosynthesis and metabolism;N-Glycan_biosynthesis | 0.000331 | 9.81E-06 | 0.000253 | 4.94E-05 | 0.010931 | 0.060087 | 2.64E-05 | 0.00013 |
| Metabolism;Metabolism of terpenoids and polyketides;Biosynthesis_of_siderophore_group_nonribosomal_peptides | 0.000249 | 6.71E-05 | 0.000164 | 3.97E-05 | 0.028492 | 0.090633 | 1.14E-05 | 0.000158 |
| Human Diseases;Cancers;Renal_cell_carcinoma | 0.000173 | 2.1E-05 | 0.000284 | 5.25E-05 | 0.002352 | 0.042225 | -0.00017 | -5.5E-05 |
| Metabolism;Glycan biosynthesis and metabolism;Glycosaminoglycan_binding_proteins | 0.000171 | 1.89E-05 | 0.000106 | 2.46E-05 | 0.000544 | 0.042225 | 3.65E-05 | 9.35E-05 |
| Organismal Systems;Digestive system;Protein_digestion_and_absorption | 0.000241 | 5.39E-05 | 0.000103 | 0.000101 | 0.019433 | 0.078274 | 2.92E-05 | 0.000247 |
| Metabolism;Lipid metabolism;Steroid_hormone_biosynthesis | 0.000174 | 6.14E-05 | 8.44E-05 | 7.21E-05 | 0.043251 | 0.103127 | 3.33E-06 | 0.000176 |
| Environmental Information Processing;Signaling molecules and interaction;CD_Molecules | 0.000238 | 5.41E-05 | 9.98E-05 | 0.000103 | 0.020403 | 0.078274 | 2.82E-05 | 0.000249 |
| Organismal Systems;Digestive system;Carbohydrate_digestion_and_absorption | 0.000114 | 3.23E-05 | 7.38E-05 | 1.73E-05 | 0.02846 | 0.090633 | 5.52E-06 | 7.5E-05 |
| Metabolism;Biosynthesis of other secondary metabolites;Flavone_and_flavonol_biosynthesis | 0.000118 | 8.7E-06 | 9.64E-05 | 1.63E-05 | 0.020433 | 0.078274 | 4.45E-06 | 3.95E-05 |
| Human Diseases;Cancers;Choline_metabolism_in_cancer | 9.26E-05 | 7.18E-06 | 0.000109 | 1.08E-05 | 0.013456 | 0.065517 | -2.8E-05 | -4.3E-06 |
| Environmental Information Processing;Signal transduction;Phospholipase_D_signaling_pathway | 7.63E-05 | 6.87E-06 | 9.25E-05 | 1.34E-05 | 0.031262 | 0.094411 | -3.1E-05 | -1.9E-06 |
| Genetic Information Processing;Transcription;Basal_transcription_factors | 7.48E-05 | 9.1E-06 | 6.1E-05 | 1.11E-05 | 0.040494 | 0.101038 | 7.35E-07 | 2.69E-05 |
| Organismal Systems;Endocrine system;Renin-angiotensin_system | 6.03E-05 | 1.93E-05 | 2.88E-05 | 2.59E-05 | 0.039527 | 0.101038 | 1.87E-06 | 6.12E-05 |
| Human Diseases;Infectious diseases;African_trypanosomiasis | 3.1E-05 | 1.36E-05 | 7.93E-06 | 3.38E-06 | 0.00787 | 0.057414 | 8.83E-06 | 3.73E-05 |
| Human Diseases;Infectious diseases;Chagas_disease_(American_trypanosomiasis) | 2.83E-05 | 1.31E-05 | 5.73E-06 | 3.61E-06 | 0.007169 | 0.057414 | 8.87E-06 | 3.63E-05 |
| Human Diseases;Neurodegenerative diseases;Prion_diseases | 2.71E-05 | 3.06E-06 | 2.21E-05 | 4.03E-06 | 0.036218 | 0.098192 | 4.01E-07 | 9.7E-06 |
| Organismal Systems;Digestive system;Mineral_absorption | 1.16E-05 | 1.93E-06 | 8.81E-06 | 1.93E-06 | 0.029988 | 0.09331 | 3.34E-07 | 5.3E-06 |
| Metabolism;Metabolism of terpenoids and polyketides;Type_I_polyketide_structures | 9.57E-06 | 5.92E-06 | 3.27E-06 | 1.48E-06 | 0.047558 | 0.108059 | 9.56E-08 | 1.25E-05 |
| Metabolism;Biosynthesis of other secondary metabolites;Stilbenoid,_diarylheptanoid_and_gingerol_biosynthesis | 3.46E-06 | 5.49E-07 | 1.76E-06 | 9.04E-07 | 0.004074 | 0.042225 | 7.08E-07 | 2.69E-06 |
| Metabolism;Biosynthesis of other secondary metabolites;Flavonoid_biosynthesis | 3.46E-06 | 5.49E-07 | 1.76E-06 | 9.04E-07 | 0.004074 | 0.042225 | 7.08E-07 | 2.69E-06 |
| Metabolism;Metabolism of terpenoids and polyketides;Biosynthesis_of_12-,_14-_and_16-membered_macrolides | 3.33E-06 | 2.17E-06 | 1.02E-06 | 4.86E-07 | 0.047456 | 0.108059 | 3.67E-08 | 4.57E-06 |
| Metabolism;Metabolism of terpenoids and polyketides;Biosynthesis_of_enediyne_antibiotics | 2.58E-06 | 1.55E-06 | 8.51E-07 | 4.94E-07 | 0.040537 | 0.101038 | 1.03E-07 | 3.35E-06 |
| Human Diseases;Immune diseases;Systemic_lupus_erythematosus | 5.61E-07 | 2.55E-07 | 2.46E-07 | 9.81E-08 | 0.028032 | 0.090633 | 4.64E-08 | 5.83E-07 |
| Human Diseases;Infectious diseases;Vibrio_cholerae_infection | 7.42E-07 | 4.06E-07 | 2.66E-07 | 2.15E-07 | 0.036006 | 0.098192 | 4.04E-08 | 9.12E-07 |
| Human Diseases;Infectious diseases;Pathogenic_Escherichia_coli_infection | 6E-07 | 3.16E-07 | 1.71E-07 | 8.14E-08 | 0.019688 | 0.078274 | 9.83E-08 | 7.61E-07 |
| Organismal Systems;Endocrine system;Ovarian_Steroidogenesis | 3.18E-07 | 2.43E-07 | 6.29E-08 | 5.25E-08 | 0.049664 | 0.108091 | 5.32E-10 | 5.09E-07 |
| Environmental Information Processing;Signal transduction;Calcium_signaling_pathway | 7.35E-08 | 4.47E-08 | 1.4E-08 | 1.79E-08 | 0.02067 | 0.078274 | 1.24E-08 | 1.07E-07 |
| Environmental Information Processing;Signaling molecules and interaction;G_protein-coupled_receptors | 2.34E-08 | 1.07E-08 | 2.05E-09 | 3.26E-09 | 0.003543 | 0.042225 | 1.02E-08 | 3.26E-08 |
| Environmental Information Processing;Signaling molecules and interaction;Neuroactive_ligand-receptor_interaction | 5.88E-09 | 4.51E-09 | 1.14E-09 | 1.32E-09 | 0.049187 | 0.108091 | 2.35E-11 | 9.47E-09 |
| Metabolism;Glycan biosynthesis and metabolism;Glycosaminoglycan_biosynthesis-chondroitin_sulfate/dermatan_sulfate | 2.39E-09 | 1.68E-09 | 5.21E-10 | 8.36E-10 | 0.043658 | 0.103127 | 6.93E-11 | 3.66E-09 |
| Metabolism;Biosynthesis of other secondary metabolites;Clavulanic_acid_biosynthesis | 7.9E-10 | 6.83E-10 | 1.4E-11 | 1.12E-11 | 0.038774 | 0.100551 | 5.91E-11 | 1.49E-09 |

| Table 2b Young+PBS VS Aged+PBS | | | | | | | | |
| --- | --- | --- | --- | --- | --- | --- | --- | --- |
| Taxa | avg(Young+PBS) | sd(Young+PBS) | avg(Aged+PBS) | sd(Aged+PBS) | p.value | q.values | interval lower | interval upper |
| Metabolism;Nucleotide metabolism;Purine_metabolism | 0.025653 | 0.000514 | 0.026306 | 0.00031 | 0.027754 | 0.018099 | -0.00122 | -9.1E-05 |
| Genetic Information Processing;Translation;Transfer_RNA_biogenesis | 0.025388 | 0.000319 | 0.025764 | 0.000192 | 0.037679 | 0.020034 | -0.00072 | -2.7E-05 |
| Metabolism;Nucleotide metabolism;Pyrimidine_metabolism | 0.020031 | 0.000437 | 0.020588 | 0.000135 | 0.024899 | 0.016912 | -0.00101 | -9.9E-05 |
| Genetic Information Processing;Translation;Ribosome | 0.015723 | 0.000269 | 0.016031 | 0.000179 | 0.045392 | 0.02079 | -0.00061 | -7.9E-06 |
| Genetic Information Processing;Translation;Aminoacyl-tRNA_biosynthesis | 0.015499 | 0.000262 | 0.015904 | 0.000183 | 0.012754 | 0.011118 | -0.0007 | -0.00011 |
| Cellular Processes;Transport and catabolism;Exosome | 0.015496 | 0.000151 | 0.01467 | 0.000299 | 0.000423 | 0.001644 | 0.000506 | 0.001146 |
| Genetic Information Processing;Replication and repair;DNA_replication_proteins | 0.014807 | 0.000271 | 0.015109 | 0.000133 | 0.042432 | 0.020702 | -0.00059 | -1.4E-05 |
| Genetic Information Processing;Replication and repair;Chromosome_and_associated_proteins | 0.0131 | 0.000238 | 0.013723 | 0.000145 | 0.000519 | 0.0017 | -0.00088 | -0.00036 |
| Metabolism;Amino acid metabolism;Alanine,_aspartate_and_glutamate_metabolism | 0.011744 | 0.000114 | 0.011119 | 0.000336 | 0.004746 | 0.006652 | 0.000273 | 0.000978 |
| Genetic Information Processing;Replication and repair;Mismatch_repair | 0.010868 | 0.000292 | 0.011191 | 0.000174 | 0.047672 | 0.020993 | -0.00064 | -4.2E-06 |
| Genetic Information Processing;Replication and repair;Homologous_recombination | 0.010494 | 0.000212 | 0.010894 | 0.000219 | 0.009155 | 0.009537 | -0.00068 | -0.00012 |
| Metabolism;Glycan biosynthesis and metabolism;Peptidoglycan_biosynthesis_and_degradation_proteins | 0.010172 | 0.000138 | 0.010573 | 0.000247 | 0.008688 | 0.009515 | -0.00067 | -0.00013 |
| Metabolism;Amino acid metabolism;Cysteine_and_methionine_metabolism | 0.009602 | 7.4E-05 | 0.009323 | 5.47E-05 | 3.49E-05 | 0.000528 | 0.000195 | 0.000364 |
| Metabolism;Energy metabolism;Carbon_fixation_pathways_in_prokaryotes | 0.00918 | 0.000366 | 0.008631 | 0.000453 | 0.044292 | 0.020702 | 1.72E-05 | 0.001082 |
| Metabolism;Glycan biosynthesis and metabolism;Peptidoglycan_biosynthesis | 0.008308 | 0.000207 | 0.008721 | 0.000328 | 0.029895 | 0.018642 | -0.00077 | -5.1E-05 |
| Genetic Information Processing;Replication and repair;DNA_replication | 0.007972 | 0.000183 | 0.00835 | 0.000115 | 0.002382 | 0.004186 | -0.00058 | -0.00018 |
| Genetic Information Processing;Translation;Messenger_RNA_Biogenesis | 0.007849 | 5.8E-05 | 0.007653 | 0.000128 | 0.011143 | 0.010236 | 6.06E-05 | 0.000332 |
| Metabolism;Amino acid metabolism;Glycine,_serine_and_threonine_metabolism | 0.007893 | 0.000123 | 0.0077 | 0.000166 | 0.047234 | 0.020993 | 2.91E-06 | 0.000383 |
| Metabolism;Carbohydrate metabolism;Pentose_phosphate_pathway | 0.007552 | 0.000179 | 0.008037 | 0.000253 | 0.004043 | 0.006168 | -0.00077 | -0.0002 |
| Genetic Information Processing;Folding, sorting and degradation;RNA_degradation | 0.007475 | 8.88E-05 | 0.007297 | 0.000143 | 0.030419 | 0.018733 | 2.15E-05 | 0.000336 |
| Metabolism;Carbohydrate metabolism;Butanoate_metabolism | 0.007496 | 0.000432 | 0.006828 | 0.000131 | 0.011301 | 0.01027 | 0.000216 | 0.00112 |
| Genetic Information Processing;Transcription;Transcription_machinery | 0.007439 | 9.11E-05 | 0.006994 | 0.00041 | 0.044514 | 0.020702 | 1.55E-05 | 0.000874 |
| Metabolism;Carbohydrate metabolism;Propanoate_metabolism | 0.006937 | 0.000132 | 0.007178 | 9.95E-05 | 0.005804 | 0.007512 | -0.00039 | -8.8E-05 |
| Metabolism;Carbohydrate metabolism;Glyoxylate_and_dicarboxylate_metabolism | 0.007261 | 0.000272 | 0.006487 | 0.000322 | 0.001233 | 0.003019 | 0.000389 | 0.001159 |
| Metabolism;Carbohydrate metabolism;Citrate_cycle_(TCA_cycle) | 0.006205 | 0.000286 | 0.005779 | 0.000294 | 0.02945 | 0.018636 | 5.2E-05 | 0.000798 |
| Unclassified;Metabolism;Energy_metabolism | 0.006315 | 0.000289 | 0.005571 | 0.00032 | 0.001779 | 0.003455 | 0.000352 | 0.001137 |
| Unclassified;Poorly characterized;General_function_prediction_only | 0.005257 | 0.000243 | 0.006104 | 0.00068 | 0.027117 | 0.017958 | -0.00156 | -0.00013 |
| Metabolism;Energy metabolism;Carbon_fixation_in_photosynthetic_organisms | 0.005094 | 4.39E-05 | 0.004982 | 4.4E-05 | 0.001278 | 0.003032 | 5.58E-05 | 0.000169 |
| Metabolism;Amino acid metabolism;Arginine_biosynthesis | 0.005337 | 0.000206 | 0.004855 | 0.000135 | 0.001101 | 0.003019 | 0.000253 | 0.000711 |
| Metabolism;Amino acid metabolism;Phenylalanine,_tyrosine_and_tryptophan_biosynthesis | 0.004979 | 0.00031 | 0.004465 | 0.000266 | 0.011991 | 0.01067 | 0.000141 | 0.000886 |
| Genetic Information Processing;Folding, sorting and degradation;Protein_export | 0.004501 | 8.55E-05 | 0.00462 | 8.29E-05 | 0.0345 | 0.020034 | -0.00023 | -1.1E-05 |
| Metabolism;Lipid metabolism;Lipid_biosynthesis_proteins | 0.004748 | 0.000253 | 0.004339 | 8.86E-05 | 0.009127 | 0.009537 | 0.000143 | 0.000675 |
| Metabolism;Metabolism of terpenoids and polyketides;Terpenoid_backbone_biosynthesis | 0.004268 | 0.000118 | 0.004426 | 4.29E-05 | 0.020507 | 0.015782 | -0.00028 | -3.4E-05 |
| Metabolism;Metabolism of cofactors and vitamins;Thiamine_metabolism | 0.004472 | 6.82E-05 | 0.004227 | 0.000161 | 0.011682 | 0.010505 | 7.47E-05 | 0.000415 |
| Metabolism;Lipid metabolism;Glycerophospholipid_metabolism | 0.004116 | 0.00012 | 0.004402 | 0.00011 | 0.001578 | 0.003306 | -0.00043 | -0.00014 |
| Metabolism;Energy metabolism;Nitrogen_metabolism | 0.004843 | 0.000271 | 0.004069 | 0.000223 | 0.000336 | 0.001553 | 0.000454 | 0.001095 |
| Metabolism;Metabolism of cofactors and vitamins;One_carbon_pool_by_folate | 0.004295 | 4.61E-05 | 0.003978 | 7.5E-05 | 1.69E-05 | 0.000528 | 0.000235 | 0.0004 |
| Genetic Information Processing;Replication and repair;Base_excision_repair | 0.003915 | 6.37E-05 | 0.004133 | 8.09E-05 | 0.000485 | 0.001658 | -0.00031 | -0.00012 |
| Metabolism;Metabolism of other amino acids;Selenocompound_metabolism | 0.004118 | 5.86E-05 | 0.004009 | 5.9E-05 | 0.009112 | 0.009537 | 3.38E-05 | 0.000185 |
| Metabolism;Metabolism of cofactors and vitamins;Pantothenate_and_CoA_biosynthesis | 0.004248 | 0.000113 | 0.003982 | 4.2E-05 | 0.001355 | 0.003046 | 0.000147 | 0.000384 |
| Metabolism;Glycan biosynthesis and metabolism;Glycosyltransferases | 0.003709 | 0.000154 | 0.003989 | 0.000158 | 0.011138 | 0.010236 | -0.00048 | -7.9E-05 |
| Metabolism;Amino acid metabolism;Valine,_leucine_and_isoleucine_biosynthesis | 0.003878 | 0.000305 | 0.003302 | 0.000124 | 0.004186 | 0.006251 | 0.000254 | 0.000898 |
| Genetic Information Processing;Transcription;RNA_polymerase | 0.003305 | 7E-05 | 0.003427 | 4.68E-05 | 0.0065 | 0.007932 | -0.0002 | -4.4E-05 |
| Metabolism;Carbohydrate metabolism;Pentose_and_glucuronate_interconversions | 0.003491 | 7.67E-05 | 0.003163 | 0.000104 | 0.00014 | 0.00098 | 0.000209 | 0.000446 |
| Metabolism;Lipid metabolism;Sphingolipid_metabolism | 0.003031 | 0.000248 | 0.00258 | 0.000251 | 0.01058 | 0.010043 | 0.000131 | 0.000773 |
| Metabolism;Metabolism of cofactors and vitamins;Folate_biosynthesis | 0.002563 | 5.22E-05 | 0.002807 | 0.000132 | 0.004674 | 0.006652 | -0.00038 | -0.00011 |
| Metabolism;Biosynthesis of other secondary metabolites;Streptomycin_biosynthesis | 0.002693 | 4.16E-05 | 0.002542 | 4.81E-05 | 0.000182 | 0.001049 | 9.31E-05 | 0.000209 |
| Cellular Processes;Cellular community - prokaryotes;Biofilm_formation-Escherichia_coli | 0.002944 | 0.00017 | 0.002448 | 6.37E-05 | 0.00042 | 0.001644 | 0.000317 | 0.000675 |
| Metabolism;Metabolism of other amino acids;Glutathione_metabolism | 0.002308 | 0.000124 | 0.002741 | 0.000188 | 0.001237 | 0.003019 | -0.00064 | -0.00022 |
| Metabolism;Energy metabolism;Sulfur_metabolism | 0.0026 | 9.62E-05 | 0.00237 | 6.26E-05 | 0.000966 | 0.002947 | 0.000123 | 0.000337 |
| Metabolism;Amino acid metabolism;Histidine_metabolism | 0.002726 | 0.000184 | 0.002265 | 6.44E-05 | 0.001014 | 0.002987 | 0.000269 | 0.000654 |
| Metabolism;Xenobiotics biodegradation and metabolism;Drug_metabolism-other_enzymes | 0.002595 | 4.74E-05 | 0.002421 | 5.19E-05 | 0.000123 | 0.00098 | 0.00011 | 0.000238 |
| Metabolism;Metabolism of other amino acids;Cyanoamino_acid_metabolism | 0.002675 | 7.72E-05 | 0.002357 | 0.000201 | 0.009879 | 0.009706 | 0.000106 | 0.00053 |
| Unclassified;Cellular processes and signaling;Cell_growth | 0.002913 | 0.000282 | 0.002427 | 0.000246 | 0.009998 | 0.009706 | 0.000145 | 0.000827 |
| Cellular Processes;Cell growth and death;Necroptosis | 0.002668 | 0.000129 | 0.002126 | 0.000197 | 0.000376 | 0.001607 | 0.000323 | 0.000761 |
| Genetic Information Processing;Folding, sorting and degradation;Sulfur_relay_system | 0.002033 | 6.24E-05 | 0.002168 | 1.8E-05 | 0.002388 | 0.004186 | -0.0002 | -7E-05 |
| Organismal Systems;Aging;Longevity_regulating_pathway-worm | 0.001962 | 1.51E-05 | 0.002049 | 4.11E-05 | 0.002488 | 0.004186 | -0.00013 | -4.3E-05 |
| Metabolism;Biosynthesis of other secondary metabolites;Monobactam_biosynthesis | 0.002121 | 3.47E-05 | 0.001968 | 8.39E-05 | 0.004892 | 0.006741 | 6.45E-05 | 0.000242 |
| Metabolism;Carbohydrate metabolism;C5-Branched_dibasic_acid_metabolism | 0.002032 | 0.000102 | 0.001784 | 8.8E-05 | 0.001146 | 0.003019 | 0.000126 | 0.000371 |
| Unclassified;Cellular processes and signaling;Membrane_and_intracellular_structural_molecules | 0.001747 | 7.54E-05 | 0.001925 | 0.000136 | 0.023341 | 0.016912 | -0.00032 | -3.1E-05 |
| Cellular Processes;Cellular community - prokaryotes;Biofilm_formation-Vibrio_cholerae | 0.001887 | 0.0001 | 0.001767 | 6.28E-05 | 0.035597 | 0.020034 | 1.03E-05 | 0.000231 |
| Human Diseases;Drug resistance;Antimicrobial_resistance_genes | 0.001562 | 0.00013 | 0.001938 | 0.000318 | 0.033363 | 0.019522 | -0.00071 | -4E-05 |
| Human Diseases;Drug resistance;Cationic_antimicrobial_peptide_(CAMP)_resistance | 0.001578 | 0.000133 | 0.001938 | 0.000162 | 0.001951 | 0.003704 | -0.00055 | -0.00017 |
| Metabolism;Amino acid metabolism;Phenylalanine_metabolism | 0.001845 | 0.000192 | 0.001538 | 7.37E-05 | 0.0093 | 0.009572 | 0.000105 | 0.000509 |
| Human Diseases;Endocrine and metabolic diseases;Insulin_resistance | 0.001786 | 0.000103 | 0.001469 | 4.21E-05 | 0.000286 | 0.001439 | 0.000208 | 0.000426 |
| Metabolism;Metabolism of terpenoids and polyketides;Prenyltransferases | 0.001408 | 5.91E-05 | 0.001515 | 2.26E-05 | 0.005042 | 0.006837 | -0.00017 | -4.6E-05 |
| Metabolism;Metabolism of other amino acids;D-Glutamine_and_D-glutamate_metabolism | 0.001416 | 4.8E-05 | 0.001472 | 3.06E-05 | 0.041377 | 0.020702 | -0.00011 | -2.8E-06 |
| Environmental Information Processing;Signal transduction;HIF-1_signaling_pathway | 0.001241 | 3.17E-05 | 0.001378 | 8.59E-05 | 0.009558 | 0.009606 | -0.00023 | -4.7E-05 |
| Metabolism;Glycan biosynthesis and metabolism;Glycosphingolipid_biosynthesis-globo_and_isoglobo_series | 0.001423 | 0.000143 | 0.001238 | 7.89E-05 | 0.024677 | 0.016912 | 3.06E-05 | 0.000339 |
| Metabolism;Metabolism of other amino acids;Taurine_and_hypotaurine_metabolism | 0.001248 | 2.41E-05 | 0.001392 | 6.39E-05 | 0.00172 | 0.003455 | -0.00021 | -7.7E-05 |
| Metabolism;Biosynthesis of other secondary metabolites;Phenylpropanoid_biosynthesis | 0.001572 | 6.81E-05 | 0.001167 | 0.000172 | 0.001339 | 0.003046 | 0.000223 | 0.000587 |
| Metabolism;Metabolism of other amino acids;D-Alanine_metabolism | 0.001168 | 4.12E-05 | 0.001297 | 8.82E-05 | 0.013815 | 0.01157 | -0.00022 | -3.5E-05 |
| Cellular Processes;Transport and catabolism;Peroxisome | 0.001301 | 6.36E-05 | 0.001189 | 8.51E-05 | 0.029136 | 0.018575 | 1.41E-05 | 0.00021 |
| Organismal Systems;Nervous system;GABAergic_synapse | 0.001346 | 3.61E-05 | 0.001162 | 0.000101 | 0.00527 | 0.007034 | 7.72E-05 | 0.00029 |
| Human Diseases;Infectious diseases;Staphylococcus_aureus_infection | 0.000788 | 0.000143 | 0.001567 | 0.000581 | 0.020721 | 0.015805 | -0.00139 | -0.00017 |
| Metabolism;Metabolism of terpenoids and polyketides;Polyketide_sugar_unit_biosynthesis | 0.001273 | 3.92E-05 | 0.001102 | 4.39E-05 | 3.58E-05 | 0.000528 | 0.000117 | 0.000224 |
| Metabolism;Metabolism of cofactors and vitamins;Vitamin_B6_metabolism | 0.00127 | 5.5E-05 | 0.001147 | 5.2E-05 | 0.002645 | 0.004345 | 5.39E-05 | 0.000192 |
| Organismal Systems;Nervous system;Glutamatergic_synapse | 0.001287 | 3.41E-05 | 0.001097 | 0.000116 | 0.008547 | 0.009483 | 6.98E-05 | 0.000312 |
| Metabolism;Enzyme families;Protein_phosphatase_and_associated_proteins | 0.001301 | 7.07E-05 | 0.001026 | 0.000148 | 0.004244 | 0.006251 | 0.000118 | 0.000433 |
| Unclassified;Metabolism;Carbohydrate_metabolism | 0.001315 | 0.000147 | 0.001103 | 3.74E-05 | 0.015668 | 0.012748 | 5.76E-05 | 0.000365 |
| Unclassified;Metabolism;Amino_acid_metabolism | 0.001195 | 5.78E-05 | 0.001107 | 4E-05 | 0.013495 | 0.011414 | 2.31E-05 | 0.000153 |
| Organismal Systems;Endocrine system;Insulin_signaling_pathway | 0.001273 | 8.54E-05 | 0.001 | 6.37E-05 | 0.000126 | 0.00098 | 0.000176 | 0.000372 |
| Metabolism;Metabolism of cofactors and vitamins;Ubiquinone_and_other_terpenoid-quinone_biosynthesis | 0.000957 | 9.74E-05 | 0.001156 | 6.87E-05 | 0.002744 | 0.004423 | -0.00031 | -8.9E-05 |
| Human Diseases;Cardiovascular diseases;Fluid_shear_stress_and_atherosclerosis | 0.001166 | 2.7E-05 | 0.001026 | 9.88E-05 | 0.016498 | 0.013297 | 3.66E-05 | 0.000243 |
| Organismal Systems;Endocrine system;PPAR_signaling_pathway | 0.001209 | 6.31E-05 | 0.000967 | 3.82E-05 | 3.71E-05 | 0.000528 | 0.000172 | 0.00031 |
| Metabolism;Metabolism of other amino acids;beta-Alanine_metabolism | 0.001119 | 0.000151 | 0.000955 | 4.08E-05 | 0.04471 | 0.020702 | 5.42E-06 | 0.000322 |
| Environmental Information Processing;Signaling molecules and interaction;Bacterial_toxins | 0.000828 | 8.51E-05 | 0.001118 | 0.000234 | 0.027702 | 0.018099 | -0.00054 | -4.4E-05 |
| Unclassified;Viral protein family;Viral_proteins | 0.00087 | 4.86E-05 | 0.001018 | 4.82E-05 | 0.000345 | 0.001553 | -0.00021 | -8.6E-05 |
| Metabolism;Biosynthesis of other secondary metabolites;Novobiocin_biosynthesis | 0.001016 | 5.43E-05 | 0.000857 | 5.38E-05 | 0.000468 | 0.001658 | 8.94E-05 | 0.000229 |
| Metabolism;Carbohydrate metabolism;Inositol_phosphate_metabolism | 0.000854 | 5.25E-05 | 0.000953 | 8.36E-05 | 0.038817 | 0.020034 | -0.00019 | -6.4E-06 |
| Metabolism;Biosynthesis of other secondary metabolites;Tropane,_piperidine_and_pyridine_alkaloid_biosynthesis | 0.001043 | 6.63E-05 | 0.000836 | 5.62E-05 | 0.000184 | 0.001049 | 0.000127 | 0.000286 |
| Cellular Processes;Cellular community - prokaryotes;Biofilm_formation-Pseudomonas_aeruginosa | 0.000931 | 9.62E-05 | 0.000813 | 5E-05 | 0.030701 | 0.018733 | 1.43E-05 | 0.000221 |
| Metabolism;Biosynthesis of other secondary metabolites;Acarbose_and_validamycin_biosynthesis | 0.000751 | 2.85E-05 | 0.000637 | 2.76E-05 | 3.4E-05 | 0.000528 | 7.86E-05 | 0.000151 |
| Metabolism;Lipid metabolism;Synthesis_and_degradation_of_ketone_bodies | 0.000626 | 3.57E-05 | 0.000714 | 6.61E-05 | 0.021124 | 0.01583 | -0.00016 | -1.7E-05 |
| Metabolism;Biosynthesis of other secondary metabolites;Carbapenem_biosynthesis | 0.000704 | 1.62E-05 | 0.000657 | 3.2E-05 | 0.014556 | 0.012073 | 1.22E-05 | 8.07E-05 |
| Environmental Information Processing;Signal transduction;AMPK_signaling_pathway | 0.000694 | 4.69E-05 | 0.000558 | 5.71E-05 | 0.001228 | 0.003019 | 6.87E-05 | 0.000204 |
| Metabolism;Xenobiotics biodegradation and metabolism;Drug_metabolism-cytochrome_P450 | 0.000515 | 3.98E-05 | 0.000629 | 0.000103 | 0.042406 | 0.020702 | -0.00022 | -5.3E-06 |
| Genetic Information Processing;Folding, sorting and degradation;Protein_processing_in_endoplasmic_reticulum | 0.000645 | 2.96E-05 | 0.000512 | 0.000115 | 0.036719 | 0.020034 | 1.16E-05 | 0.000253 |
| Metabolism;Xenobiotics biodegradation and metabolism;Metabolism_of_xenobiotics_by_cytochrome_P450 | 0.000478 | 4.11E-05 | 0.00059 | 0.000101 | 0.041636 | 0.020702 | -0.00022 | -5.7E-06 |
| Environmental Information Processing;Signal transduction;Phosphatidylinositol_signaling_system | 0.000471 | 3.16E-05 | 0.000551 | 3.83E-05 | 0.002862 | 0.004527 | -0.00013 | -3.5E-05 |
| Metabolism;Metabolism of cofactors and vitamins;Retinol_metabolism | 0.000448 | 4.19E-05 | 0.000565 | 9.17E-05 | 0.024724 | 0.016912 | -0.00021 | -2E-05 |
| Unclassified;Metabolism;Nucleotide_metabolism | 0.000424 | 6.16E-05 | 0.000559 | 7.62E-05 | 0.007571 | 0.00874 | -0.00022 | -4.5E-05 |
| Unclassified;Metabolism;Biosynthesis_and_biodegradation_of_secondary_metabolites | 0.000552 | 8.08E-05 | 0.000446 | 4.36E-05 | 0.022652 | 0.016682 | 1.95E-05 | 0.000194 |
| Cellular Processes;Cell growth and death;Ferroptosis | 0.000572 | 3.2E-05 | 0.000384 | 4.92E-05 | 3.43E-05 | 0.000528 | 0.000133 | 0.000242 |
| Environmental Information Processing;Signal transduction;PI3K-Akt_signaling_pathway | 0.000531 | 3.76E-05 | 0.000404 | 0.000105 | 0.029838 | 0.018642 | 1.7E-05 | 0.000237 |
| Metabolism;Metabolism of terpenoids and polyketides;Biosynthesis_of_vancomycin_group_antibiotics | 0.000483 | 1.79E-05 | 0.000414 | 2.67E-05 | 0.000537 | 0.0017 | 3.98E-05 | 9.93E-05 |
| Genetic Information Processing;Folding, sorting and degradation;Proteasome | 0.000566 | 5.42E-05 | 0.000391 | 0.000103 | 0.006859 | 0.008253 | 6.44E-05 | 0.000286 |
| Organismal Systems;Endocrine system;Adipocytokine_signaling_pathway | 0.000557 | 4.04E-05 | 0.00034 | 6.34E-05 | 7.79E-05 | 0.000832 | 0.000147 | 0.000287 |
| Metabolism;Biosynthesis of other secondary metabolites;Isoquinoline_alkaloid_biosynthesis | 0.000477 | 1.95E-05 | 0.000398 | 4.93E-05 | 0.008874 | 0.009537 | 2.78E-05 | 0.000132 |
| Human Diseases;Cancers;Prostate_cancer | 0.000502 | 2.81E-05 | 0.000392 | 0.000103 | 0.046481 | 0.02079 | 2.4E-06 | 0.000217 |
| Organismal Systems;Immune system;IL-17_signaling_pathway | 0.000497 | 2.85E-05 | 0.000387 | 0.000101 | 0.044528 | 0.020702 | 3.75E-06 | 0.000216 |
| Organismal Systems;Endocrine system;Estrogen_signaling_pathway | 0.000497 | 2.84E-05 | 0.000387 | 0.000101 | 0.044832 | 0.020702 | 3.53E-06 | 0.000216 |
| Organismal Systems;Endocrine system;Progesterone-mediated_oocyte_maturation | 0.000497 | 2.84E-05 | 0.000387 | 0.000101 | 0.044832 | 0.020702 | 3.53E-06 | 0.000216 |
| Organismal Systems;Immune system;Th17_cell_differentiation | 0.000497 | 2.84E-05 | 0.000387 | 0.000101 | 0.044832 | 0.020702 | 3.53E-06 | 0.000216 |
| Organismal Systems;Immune system;Antigen_processing_and_presentation | 0.000497 | 2.84E-05 | 0.000387 | 0.000101 | 0.044832 | 0.020702 | 3.53E-06 | 0.000216 |
| Metabolism;Metabolism of other amino acids;Phosphonate_and_phosphinate_metabolism | 0.000382 | 2.97E-05 | 0.000323 | 2.78E-05 | 0.005649 | 0.007424 | 2.13E-05 | 9.54E-05 |
| Metabolism;Biosynthesis of other secondary metabolites;Glucosinolate_biosynthesis | 0.000341 | 3.28E-05 | 0.000272 | 1.21E-05 | 0.002427 | 0.004186 | 3.48E-05 | 0.000104 |
| Unclassified;Genetic information processing;Translation_proteins | 0.000322 | 2.64E-05 | 0.000271 | 1.91E-05 | 0.003657 | 0.00568 | 2.15E-05 | 8.15E-05 |
| Genetic Information Processing;Translation;Ribosome_biogenesis_in_eukaryotes | 0.000305 | 7.95E-06 | 0.000294 | 8E-06 | 0.031883 | 0.01918 | 1.21E-06 | 2.17E-05 |
| Environmental Information Processing;Signal transduction;FoxO_signaling_pathway | 0.0002 | 3.42E-05 | 0.000294 | 5.37E-05 | 0.006194 | 0.007669 | -0.00015 | -3.5E-05 |
| Metabolism;Glycan biosynthesis and metabolism;N-Glycan_biosynthesis | 0.000331 | 9.81E-06 | 0.000253 | 2.52E-05 | 0.000283 | 0.001439 | 5.17E-05 | 0.000105 |
| Organismal Systems;Aging;Longevity_regulating_pathway-mammal | 0.00017 | 2.6E-05 | 0.000284 | 5.38E-05 | 0.002205 | 0.004094 | -0.00017 | -5.6E-05 |
| Human Diseases;Cancers;Renal_cell_carcinoma | 0.000173 | 2.1E-05 | 0.000251 | 5.73E-06 | 0.000149 | 0.00098 | -0.0001 | -5.6E-05 |
| Environmental Information Processing;Signal transduction;MAPK_signaling_pathway-plant | 0.000144 | 2.46E-05 | 0.000255 | 4.18E-05 | 0.000468 | 0.001658 | -0.00016 | -6.6E-05 |
| Human Diseases;Neurodegenerative diseases;Huntingtons_disease | 0.000151 | 1.32E-05 | 0.000223 | 5.85E-05 | 0.028429 | 0.01826 | -0.00013 | -1.1E-05 |
| Metabolism;Metabolism of terpenoids and polyketides;Nonribosomal_peptide_structures | 0.000151 | 7.28E-06 | 0.000191 | 2.38E-05 | 0.007776 | 0.008857 | -6.5E-05 | -1.5E-05 |
| Metabolism;Glycan biosynthesis and metabolism;Glycosaminoglycan_binding_proteins | 0.000171 | 1.89E-05 | 0.000129 | 2.28E-05 | 0.005956 | 0.007594 | 1.53E-05 | 6.95E-05 |
| Organismal Systems;Digestive system;Protein_digestion_and_absorption | 0.000241 | 5.39E-05 | 0.000134 | 5.61E-05 | 0.007495 | 0.00874 | 3.53E-05 | 0.000177 |
| Environmental Information Processing;Signaling molecules and interaction;CD_Molecules | 0.000238 | 5.41E-05 | 0.000129 | 5.88E-05 | 0.007326 | 0.008693 | 3.68E-05 | 0.000182 |
| Human Diseases;Neurodegenerative diseases;Amyotrophic_lateral_sclerosis_(ALS) | 9.5E-05 | 1.98E-05 | 0.000171 | 3.73E-05 | 0.002499 | 0.004186 | -0.00012 | -3.6E-05 |
| Genetic Information Processing;Folding, sorting and degradation;Ubiquitin_system | 0.000153 | 3.86E-06 | 0.00016 | 4.01E-06 | 0.009425 | 0.009585 | -1.2E-05 | -2.2E-06 |
| Environmental Information Processing;Signal transduction;MAPK_signaling_pathway-yeast | 6.68E-05 | 1.96E-05 | 0.000151 | 3.85E-05 | 0.001746 | 0.003455 | -0.00013 | -4.3E-05 |
| Environmental Information Processing;Signal transduction;MAPK_signaling_pathwayfly | 0.000104 | 8.44E-06 | 0.000133 | 1.86E-05 | 0.009917 | 0.009706 | -4.9E-05 | -9.5E-06 |
| Metabolism;Biosynthesis of other secondary metabolites;Flavone_and_flavonol_biosynthesis | 0.000118 | 8.7E-06 | 0.000105 | 5.92E-06 | 0.012997 | 0.011216 | 3.58E-06 | 2.31E-05 |
| Human Diseases;Cancers;Choline_metabolism_in_cancer | 9.26E-05 | 7.18E-06 | 0.000117 | 5.01E-06 | 9.04E-05 | 0.000858 | -3.2E-05 | -1.6E-05 |
| Genetic Information Processing;Replication and repair;Non-homologous_end-joining | 9.66E-05 | 1.61E-05 | 7.88E-05 | 7.5E-06 | 0.043816 | 0.020702 | 6.49E-07 | 3.49E-05 |
| Environmental Information Processing;Signal transduction;Phospholipase_D_signaling_pathway | 7.63E-05 | 6.87E-06 | 0.000102 | 6.17E-06 | 4.38E-05 | 0.000535 | -3.4E-05 | -1.8E-05 |
| Genetic Information Processing;Transcription;Basal_transcription_factors | 7.48E-05 | 9.1E-06 | 5.9E-05 | 4.71E-06 | 0.006124 | 0.007669 | 6.02E-06 | 2.55E-05 |
| Metabolism;Lipid metabolism;alpha-Linolenic_acid_metabolism | 3.57E-05 | 1.21E-05 | 5.48E-05 | 1.65E-05 | 0.04747 | 0.020993 | -3.8E-05 | -2.6E-07 |
| Organismal Systems;Endocrine system;Renin-angiotensin_system | 6.03E-05 | 1.93E-05 | 2.79E-05 | 1.29E-05 | 0.007966 | 0.008954 | 1.09E-05 | 5.39E-05 |
| Cellular Processes;Cell growth and death;Meiosis-yeast | 3.84E-05 | 1.22E-05 | 1.95E-05 | 1.4E-06 | 0.012394 | 0.010916 | 6.11E-06 | 3.16E-05 |
| Metabolism;Xenobiotics biodegradation and metabolism;Steroid_degradation | 4.6E-05 | 2.1E-05 | 2.09E-05 | 2.37E-06 | 0.0326 | 0.01934 | 3.06E-06 | 4.71E-05 |
| Human Diseases;Infectious diseases;African_trypanosomiasis | 3.1E-05 | 1.36E-05 | 1.27E-05 | 5.55E-06 | 0.019918 | 0.015469 | 3.94E-06 | 3.26E-05 |
| Human Diseases;Infectious diseases;Chagas_disease_(American_trypanosomiasis) | 2.83E-05 | 1.31E-05 | 9.67E-06 | 5.34E-06 | 0.015649 | 0.012748 | 4.83E-06 | 3.25E-05 |
| Human Diseases;Neurodegenerative diseases;Prion_diseases | 2.71E-05 | 3.06E-06 | 1.99E-05 | 2.06E-06 | 0.001095 | 0.003019 | 3.76E-06 | 1.06E-05 |
| Metabolism;Metabolism of terpenoids and polyketides;Type_I_polyketide_structures | 9.57E-06 | 5.92E-06 | 1.81E-06 | 4.14E-07 | 0.02367 | 0.016912 | 1.54E-06 | 1.4E-05 |
| Metabolism;Lipid metabolism;Steroid_biosynthesis | 4.22E-06 | 2.71E-06 | 1.11E-06 | 4.34E-07 | 0.037436 | 0.020034 | 2.62E-07 | 5.95E-06 |
| Genetic Information Processing;Replication and repair;Fanconi_anemia_pathway | 1.83E-06 | 3.84E-07 | 1.22E-06 | 2.53E-07 | 0.010324 | 0.00991 | 1.85E-07 | 1.04E-06 |
| Human Diseases;Cancers;Bladder_cancer | 6.04E-06 | 3.41E-06 | 8.4E-07 | 2.57E-07 | 0.013351 | 0.011405 | 1.63E-06 | 8.78E-06 |
| Metabolism;Biosynthesis of other secondary metabolites;Stilbenoid,_diarylheptanoid_and_gingerol_biosynthesis | 3.46E-06 | 5.49E-07 | 2.61E-06 | 4.99E-07 | 0.019051 | 0.015042 | 1.71E-07 | 1.52E-06 |
| Metabolism;Biosynthesis of other secondary metabolites;Flavonoid_biosynthesis | 3.46E-06 | 5.49E-07 | 2.61E-06 | 4.99E-07 | 0.019051 | 0.015042 | 1.71E-07 | 1.52E-06 |
| Organismal Systems;Digestive system;Bile_secretion | 2.19E-06 | 1.48E-06 | 5.04E-07 | 1.75E-07 | 0.038054 | 0.020034 | 1.36E-07 | 3.24E-06 |
| Organismal Systems;Endocrine system;Oxytocin_signaling_pathway | 1.42E-06 | 9.88E-07 | 2.69E-07 | 1.46E-07 | 0.035613 | 0.020034 | 1.13E-07 | 2.18E-06 |
| Metabolism;Metabolism of terpenoids and polyketides;Biosynthesis_of_12-,_14-_and_16-membered_macrolides | 3.33E-06 | 2.17E-06 | 5.4E-07 | 1.34E-07 | 0.025208 | 0.016956 | 5.16E-07 | 5.06E-06 |
| Genetic Information Processing;Translation;mRNA_surveillance_pathway | 6.58E-07 | 4.11E-07 | 2.08E-07 | 5.96E-08 | 0.043396 | 0.020702 | 1.94E-08 | 8.8E-07 |
| Metabolism;Metabolism of terpenoids and polyketides;Biosynthesis_of_enediyne_antibiotics | 2.58E-06 | 1.55E-06 | 4.91E-07 | 2.05E-07 | 0.021067 | 0.01583 | 4.64E-07 | 3.71E-06 |
| Environmental Information Processing;Signal transduction;cAMP_signaling_pathway | 3.15E-06 | 2.21E-06 | 3.89E-07 | 1.16E-07 | 0.027999 | 0.018121 | 4.44E-07 | 5.08E-06 |
| Metabolism;Xenobiotics biodegradation and metabolism;Bisphenol_degradation | 2.34E-06 | 1.44E-06 | 5.92E-07 | 3.24E-07 | 0.030626 | 0.018733 | 2.34E-07 | 3.25E-06 |
| Metabolism;Biosynthesis of other secondary metabolites;Isoflavonoid_biosynthesis | 4.08E-07 | 1.61E-07 | 1.97E-06 | 1.41E-06 | 0.041675 | 0.020702 | -3E-06 | -8.6E-08 |
| Organismal Systems;Nervous system;Retrograde_endocannabinoid_signaling | 1.11E-06 | 7.26E-07 | 2.69E-07 | 1.64E-07 | 0.035054 | 0.020034 | 8.47E-08 | 1.6E-06 |
| Organismal Systems;Endocrine system;Regulation_of_lipolysis_in_adipocyte | 1.11E-06 | 7.26E-07 | 2.69E-07 | 1.64E-07 | 0.035054 | 0.020034 | 8.47E-08 | 1.6E-06 |
| Human Diseases;Cardiovascular diseases;Hypertrophic_cardiomyopathy_(HCM) | 2.38E-07 | 9.33E-08 | 3.92E-09 | 8.5E-09 | 0.001587 | 0.003306 | 1.36E-07 | 3.32E-07 |
| Organismal Systems;Endocrine system;Renin_secretion | 2.38E-07 | 9.33E-08 | 3.92E-09 | 8.5E-09 | 0.001587 | 0.003306 | 1.36E-07 | 3.32E-07 |
| Environmental Information Processing;Signal transduction;Sphingolipid_signaling_pathway | 7.27E-07 | 4.34E-07 | 1.8E-07 | 8.06E-08 | 0.026428 | 0.017638 | 9.32E-08 | 1E-06 |
| Human Diseases;Infectious diseases;Vibrio_cholerae_infection | 7.42E-07 | 4.06E-07 | 2.59E-07 | 4.56E-08 | 0.032847 | 0.019352 | 5.79E-08 | 9.08E-07 |
| Organismal Systems;Immune system;Fc_gamma_R-mediated_phagocytosis | 7.14E-07 | 4.29E-07 | 1.63E-07 | 8.71E-08 | 0.02463 | 0.016912 | 1.02E-07 | 1E-06 |
| Cellular Processes;Transport and catabolism;Endocytosis | 7.14E-07 | 4.29E-07 | 1.63E-07 | 8.71E-08 | 0.02463 | 0.016912 | 1.02E-07 | 1E-06 |
| Organismal Systems;Endocrine system;GnRH_signaling_pathway | 7.14E-07 | 4.29E-07 | 1.63E-07 | 8.71E-08 | 0.02463 | 0.016912 | 1.02E-07 | 1E-06 |
| Environmental Information Processing;Signal transduction;Ras_signaling_pathway | 7.14E-07 | 4.29E-07 | 1.63E-07 | 8.71E-08 | 0.02463 | 0.016912 | 1.02E-07 | 1E-06 |
| Human Diseases;Infectious diseases;Pathogenic_Escherichia_coli_infection | 6E-07 | 3.16E-07 | 2.38E-07 | 1.56E-07 | 0.038692 | 0.020034 | 2.47E-08 | 7E-07 |
| Metabolism;Glycan biosynthesis and metabolism;Glycosylphosphatidylinositol(GPI)-anchor_biosynthesis | 8.38E-07 | 7.9E-07 | 8.43E-09 | 6.81E-09 | 0.049832 | 0.021831 | 8.95E-10 | 1.66E-06 |
| Organismal Systems;Endocrine system;Ovarian_Steroidogenesis | 3.18E-07 | 2.43E-07 | 2.55E-08 | 1.25E-08 | 0.031973 | 0.01918 | 3.73E-08 | 5.47E-07 |
| Organismal Systems;Digestive system;Pancreatic_secretion | 2.51E-07 | 1.78E-07 | 4.81E-08 | 3.38E-08 | 0.037784 | 0.020034 | 1.65E-08 | 3.89E-07 |
| Environmental Information Processing;Signal transduction;cGMP-PKG_signaling_pathway | 2.45E-07 | 1.74E-07 | 4.81E-08 | 3.38E-08 | 0.038398 | 0.020034 | 1.52E-08 | 3.79E-07 |
| Organismal Systems;Circulatory system;Adrenergic_signaling_in_cardiomyocytes | 2.45E-07 | 1.74E-07 | 4.81E-08 | 3.38E-08 | 0.038398 | 0.020034 | 1.52E-08 | 3.79E-07 |
| Organismal Systems;Endocrine system;Insulin_secretion | 2.45E-07 | 1.74E-07 | 4.81E-08 | 3.38E-08 | 0.038398 | 0.020034 | 1.52E-08 | 3.79E-07 |
| Organismal Systems;Digestive system;Salivary_secretion | 2.45E-07 | 1.74E-07 | 4.81E-08 | 3.38E-08 | 0.038398 | 0.020034 | 1.52E-08 | 3.79E-07 |
| Organismal Systems;Excretory system;Endocrine_and_other_factor-regulated_calcium_reabsorption | 2.45E-07 | 1.74E-07 | 4.81E-08 | 3.38E-08 | 0.038398 | 0.020034 | 1.52E-08 | 3.79E-07 |
| Organismal Systems;Digestive system;Gastric_acid_secretion | 2.45E-07 | 1.74E-07 | 4.81E-08 | 3.38E-08 | 0.038398 | 0.020034 | 1.52E-08 | 3.79E-07 |
| Organismal Systems;Excretory system;Aldosterone-regulated_sodium_reabsorption | 2.45E-07 | 1.74E-07 | 4.81E-08 | 3.38E-08 | 0.038398 | 0.020034 | 1.52E-08 | 3.79E-07 |
| Organismal Systems;Nervous system;Cholinergic_synapse | 4.28E-08 | 1.78E-08 | 2.62E-07 | 1.94E-07 | 0.038928 | 0.020034 | -4.2E-07 | -1.6E-08 |
| Environmental Information Processing;Signal transduction;Calcium_signaling_pathway | 7.35E-08 | 4.47E-08 | 1.74E-09 | 1.24E-09 | 0.011002 | 0.010236 | 2.49E-08 | 1.19E-07 |
| Cellular Processes;Transport and catabolism;Phagosome | 2.94E-08 | 2.02E-08 | 6.79E-09 | 5.38E-09 | 0.040009 | 0.020467 | 1.45E-09 | 4.37E-08 |
| Environmental Information Processing;Signal transduction;TNF_signaling_pathway | 2.23E-07 | 2.06E-07 | 1.38E-09 | 9.89E-10 | 0.046451 | 0.02079 | 5.12E-09 | 4.37E-07 |
| Human Diseases;Infectious diseases;Leishmaniasis | 2.23E-07 | 2.06E-07 | 1.38E-09 | 9.89E-10 | 0.046451 | 0.02079 | 5.12E-09 | 4.37E-07 |
| Environmental Information Processing;Signal transduction;VEGF_signaling_pathway | 2.23E-07 | 2.06E-07 | 1.38E-09 | 9.89E-10 | 0.046451 | 0.02079 | 5.12E-09 | 4.37E-07 |
| Environmental Information Processing;Signal transduction;NF-kappa_B_signaling_pathway | 2.23E-07 | 2.06E-07 | 1.38E-09 | 9.89E-10 | 0.046451 | 0.02079 | 5.12E-09 | 4.37E-07 |
| Environmental Information Processing;Signaling molecules and interaction;G_protein-coupled_receptors | 2.34E-08 | 1.07E-08 | 3.9E-09 | 5.34E-09 | 0.00475 | 0.006652 | 8.08E-09 | 3.1E-08 |
| Metabolism;Metabolism of terpenoids and polyketides;Monoterpenoid_biosynthesis | 1.94E-08 | 1.38E-08 | 1.86E-10 | 1.57E-10 | 0.019193 | 0.015042 | 4.7E-09 | 3.37E-08 |
| Organismal Systems;Excretory system;Vasopressin-regulated_water_reabsorption | 4.89E-08 | 3.88E-08 | 5.81E-09 | 5.23E-09 | 0.0417 | 0.020702 | 2.36E-09 | 8.37E-08 |
| Organismal Systems;Nervous system;Synaptic_vesicle_cycle | 4.89E-08 | 3.88E-08 | 5.81E-09 | 5.23E-09 | 0.0417 | 0.020702 | 2.36E-09 | 8.37E-08 |
| Organismal Systems;Immune system;Hematopoietic_cell_lineage | 3.63E-08 | 2.98E-08 | 4.63E-10 | 3.75E-10 | 0.032105 | 0.01918 | 4.55E-09 | 6.71E-08 |
| Environmental Information Processing;Signaling molecules and interaction;Neuroactive_ligand-receptor_interaction | 5.88E-09 | 4.51E-09 | 5.53E-11 | 5.24E-11 | 0.024944 | 0.016912 | 1.09E-09 | 1.06E-08 |
| Metabolism;Glycan biosynthesis and metabolism;Glycosaminoglycan_biosynthesis-chondroitin_sulfate/dermatan_sulfate | 2.39E-09 | 1.68E-09 | 1.75E-10 | 3.9E-10 | 0.022401 | 0.016641 | 4.52E-10 | 3.97E-09 |
| Metabolism;Biosynthesis of other secondary metabolites;Clavulanic_acid_biosynthesis | 7.9E-10 | 6.83E-10 | 3.61E-11 | 5.72E-11 | 0.042469 | 0.020702 | 3.76E-11 | 1.47E-09 |

| Table 2c Young+PBS VS Young+Old | | | | | | | | |
| --- | --- | --- | --- | --- | --- | --- | --- | --- |
| Taxa | avg(Young+PBS) | sd(Young+PBS) | avg(Young+Old) | sd(Young+Old) | p.value | q.values | interval lower | interval upper |
| Human Diseases;Endocrine and metabolic diseases;Insulin_resistance | 0.001786 | 0.000103 | 0.001621 | 0.000144 | 0.048676 | 1 | 1.19E-06 | 0.000329 |
| Organismal Systems;Nervous system;GABAergic_synapse | 0.001346 | 3.61E-05 | 0.001278 | 5.28E-05 | 0.030139 | 1 | 8.12E-06 | 0.000127 |
| Organismal Systems;Nervous system;Glutamatergic_synapse | 0.001287 | 3.41E-05 | 0.00122 | 4.1E-05 | 0.011934 | 1 | 1.85E-05 | 0.000116 |
| Human Diseases;Immune diseases;Primary_immunodeficiency | 0.00037 | 1.16E-05 | 0.000352 | 1.56E-05 | 0.048671 | 1 | 1.3E-07 | 3.59E-05 |

| Table 2d Young+PBS VS Young+Aged | | | | | | | | |
| --- | --- | --- | --- | --- | --- | --- | --- | --- |
| Taxa | avg(Young+PBS) | sd(Young+PBS) | avg(Young+Aged) | sd(Young+Aged) | p.value | q.values | interval lower | interval upper |
| Genetic Information Processing;Replication and repair;DNA_repair_and_recombination_proteins | 0.035677 | 0.000782 | 0.036768 | 0.000855 | 0.043973 | 0.134546 | -0.00215 | ###### |
| Genetic Information Processing;Translation;Transfer_RNA_biogenesis | 0.025388 | 0.000319 | 0.026138 | 0.000489 | 0.012424 | 0.090629 | -0.00129 | -0.00021 |
| Metabolism;Nucleotide metabolism;Pyrimidine_metabolism | 0.020031 | 0.000437 | 0.02071 | 0.000134 | 0.01107 | 0.090629 | -0.00114 | -0.00022 |
| Genetic Information Processing;Translation;Ribosome | 0.015723 | 0.000269 | 0.016378 | 0.000499 | 0.023175 | 0.119455 | -0.00119 | -0.00012 |
| Genetic Information Processing;Translation;Aminoacyl-tRNA_biosynthesis | 0.015499 | 0.000262 | 0.016244 | 0.000466 | 0.009445 | 0.087226 | -0.00125 | -0.00024 |
| Genetic Information Processing;Replication and repair;DNA_replication_proteins | 0.014807 | 0.000271 | 0.015335 | 0.00017 | 0.003349 | 0.069354 | -0.00083 | -0.00023 |
| Genetic Information Processing;Replication and repair;Chromosome_and_associated_proteins | 0.0131 | 0.000238 | 0.013777 | 0.000364 | 0.004466 | 0.070819 | -0.00108 | -0.00027 |
| Metabolism;Carbohydrate metabolism;Amino_sugar_and_nucleotide_sugar_metabolism | 0.012636 | 0.000357 | 0.01204 | 0.000343 | 0.014709 | 0.094612 | 0.000145 | 0.001046 |
| Metabolism;Amino acid metabolism;Alanine,_aspartate_and_glutamate_metabolism | 0.011744 | 0.000114 | 0.011257 | 0.000426 | 0.037153 | 0.134546 | 4.10E-05 | 0.000934 |
| Genetic Information Processing;Replication and repair;Mismatch_repair | 0.010868 | 0.000292 | 0.011363 | 0.00034 | 0.022536 | 0.119455 | -0.0009 | ###### |
| Genetic Information Processing;Replication and repair;Homologous_recombination | 0.010494 | 0.000212 | 0.010857 | 0.00027 | 0.027869 | 0.123562 | -0.00068 | ###### |
| Metabolism;Glycan biosynthesis and metabolism;Peptidoglycan_biosynthesis_and_degradation_proteins | 0.010172 | 0.000138 | 0.010445 | 0.000238 | 0.041075 | 0.134546 | -0.00053 | ###### |
| Metabolism;Amino acid metabolism;Cysteine_and_methionine_metabolism | 0.009602 | 7.40E-05 | 0.009104 | 0.000299 | 0.008535 | 0.087226 | 0.000185 | 0.000811 |
| Genetic Information Processing;Replication and repair;Nucleotide_excision_repair | 0.008149 | 0.000239 | 0.008565 | 0.00011 | 0.006039 | 0.07585 | -0.00067 | -0.00016 |
| Genetic Information Processing;Replication and repair;DNA_replication | 0.007972 | 0.000183 | 0.008364 | 0.000195 | 0.004922 | 0.07272 | -0.00064 | -0.00015 |
| Metabolism;Carbohydrate metabolism;Glyoxylate_and_dicarboxylate_metabolism | 0.007261 | 0.000272 | 0.006725 | 0.000464 | 0.040322 | 0.134546 | 3.01E-05 | 0.001041 |
| Metabolism;Amino acid metabolism;Arginine_biosynthesis | 0.005337 | 0.000206 | 0.004779 | 0.000157 | 0.000449 | 0.069354 | 0.00032 | 0.000796 |
| Metabolism;Metabolism of terpenoids and polyketides;Terpenoid_backbone_biosynthesis | 0.004268 | 0.000118 | 0.004527 | 0.000127 | 0.004455 | 0.070819 | -0.00042 | -0.0001 |
| Metabolism;Energy metabolism;Nitrogen_metabolism | 0.004843 | 0.000271 | 0.004204 | 0.000351 | 0.005994 | 0.07585 | 0.000232 | 0.001047 |
| Genetic Information Processing;Replication and repair;Base_excision_repair | 0.003915 | 6.37E-05 | 0.0041 | 8.30E-05 | 0.001761 | 0.069354 | -0.00028 | ###### |
| Metabolism;Lipid metabolism;Fatty_acid_biosynthesis | 0.004229 | 6.55E-05 | 0.004002 | 0.000185 | 0.028734 | 0.124876 | 3.25E-05 | 0.000422 |
| Metabolism;Metabolism of cofactors and vitamins;Pantothenate_and_CoA_biosynthesis | 0.004248 | 0.000113 | 0.00396 | 0.000119 | 0.001548 | 0.069354 | 0.000139 | 0.000437 |
| Metabolism;Glycan biosynthesis and metabolism;Glycosyltransferases | 0.003709 | 0.000154 | 0.003935 | 0.000121 | 0.018867 | 0.10722 | -0.00041 | ###### |
| Metabolism;Amino acid metabolism;Valine,_leucine_and_isoleucine_biosynthesis | 0.003878 | 0.000305 | 0.003116 | 0.000348 | 0.002468 | 0.069354 | 0.00034 | 0.001184 |
| Genetic Information Processing;Transcription;RNA_polymerase | 0.003305 | 7.00E-05 | 0.003506 | 0.000183 | 0.042956 | 0.134546 | -0.00039 | ###### |
| Metabolism;Carbohydrate metabolism;Pentose_and_glucuronate_interconversions | 0.003491 | 7.67E-05 | 0.003023 | 0.000435 | 0.045648 | 0.134546 | 1.30E-05 | 0.000923 |
| Metabolism;Glycan biosynthesis and metabolism;Other_glycan_degradation | 0.002999 | 0.000399 | 0.00215 | 0.000652 | 0.025313 | 0.121816 | 0.000134 | 0.001565 |
| Metabolism;Lipid metabolism;Sphingolipid_metabolism | 0.003031 | 0.000248 | 0.002382 | 0.000423 | 0.011633 | 0.090629 | 0.000189 | 0.001111 |
| Cellular Processes;Cell motility;Cytoskeleton_proteins | 0.002762 | 6.51E-05 | 0.002963 | 0.00012 | 0.00768 | 0.086047 | -0.00033 | ###### |
| Cellular Processes;Cellular community - prokaryotes;Biofilm_formation-Escherichia_coli | 0.002944 | 0.00017 | 0.002471 | 0.00033 | 0.01533 | 0.094612 | 0.00012 | 0.000827 |
| Metabolism;Metabolism of other amino acids;Glutathione_metabolism | 0.002308 | 0.000124 | 0.002636 | 0.000165 | 0.003442 | 0.069354 | -0.00052 | -0.00014 |
| Metabolism;Energy metabolism;Sulfur_metabolism | 0.0026 | 9.62E-05 | 0.002303 | 0.000253 | 0.033449 | 0.134546 | 3.18E-05 | 0.000563 |
| Metabolism;Amino acid metabolism;Histidine_metabolism | 0.002726 | 0.000184 | 0.002175 | 0.000457 | 0.030885 | 0.131643 | 6.87E-05 | 0.001033 |
| Metabolism;Metabolism of other amino acids;Cyanoamino_acid_metabolism | 0.002675 | 7.72E-05 | 0.002398 | 0.000263 | 0.048672 | 0.134546 | 2.25E-06 | 0.000552 |
| Cellular Processes;Cell growth and death;Necroptosis | 0.002668 | 0.000129 | 0.00226 | 0.000271 | 0.012258 | 0.090629 | 0.000119 | 0.000696 |
| Metabolism;Biosynthesis of other secondary metabolites;Monobactam_biosynthesis | 0.002121 | 3.47E-05 | 0.002002 | 8.44E-05 | 0.016158 | 0.094612 | 3.02E-05 | 0.000208 |
| Metabolism;Carbohydrate metabolism;C5-Branched_dibasic_acid_metabolism | 0.002032 | 0.000102 | 0.001725 | 0.000275 | 0.040128 | 0.134546 | 1.89E-05 | 0.000597 |
| Human Diseases;Endocrine and metabolic diseases;Insulin_resistance | 0.001786 | 0.000103 | 0.001511 | 0.000136 | 0.003091 | 0.069354 | 0.000119 | 0.000432 |
| Metabolism;Metabolism of terpenoids and polyketides;Prenyltransferases | 0.001408 | 5.91E-05 | 0.001536 | 0.000108 | 0.035041 | 0.134546 | -0.00024 | ###### |
| Metabolism;Metabolism of other amino acids;D-Glutamine_and_D-glutamate_metabolism | 0.001416 | 4.80E-05 | 0.001513 | 1.80E-05 | 0.003163 | 0.069354 | -0.00015 | ###### |
| Metabolism;Glycan biosynthesis and metabolism;Glycosphingolipid_biosynthesis-globo_and_isoglobo_series | 0.001423 | 0.000143 | 0.001089 | 0.000203 | 0.009381 | 0.087226 | 0.000104 | 0.000562 |
| Metabolism;Biosynthesis of other secondary metabolites;Phenylpropanoid_biosynthesis | 0.001572 | 6.81E-05 | 0.001168 | 0.000266 | 0.012665 | 0.090629 | 0.000125 | 0.000683 |
| Metabolism;Metabolism of other amino acids;D-Alanine_metabolism | 0.001168 | 4.12E-05 | 0.001231 | 5.04E-05 | 0.039106 | 0.134546 | -0.00012 | ###### |
| Metabolism;Enzyme families;Protein_phosphatase_and_associated_proteins | 0.001301 | 7.07E-05 | 0.001132 | 0.000133 | 0.026147 | 0.121816 | 2.62E-05 | 0.000312 |
| Unclassified;Metabolism;Carbohydrate_metabolism | 0.001315 | 0.000147 | 0.001074 | 0.000171 | 0.026382 | 0.121816 | 3.48E-05 | 0.000446 |
| Unclassified;Metabolism;Amino_acid_metabolism | 0.001195 | 5.78E-05 | 0.000991 | 0.000177 | 0.036034 | 0.134546 | 1.84E-05 | 0.00039 |
| Organismal Systems;Endocrine system;Insulin_signaling_pathway | 0.001273 | 8.54E-05 | 0.001024 | 0.000108 | 0.001465 | 0.069354 | 0.000123 | 0.000375 |
| Human Diseases;Cardiovascular diseases;Fluid_shear_stress_and_atherosclerosis | 0.001166 | 2.70E-05 | 0.001097 | 5.46E-05 | 0.025061 | 0.121816 | 1.15E-05 | 0.000128 |
| Unclassified;Viral protein family;Viral_proteins | 0.00087 | 4.86E-05 | 0.000977 | 7.30E-05 | 0.016166 | 0.094612 | -0.00019 | ###### |
| Metabolism;Glycan biosynthesis and metabolism;Glycosaminoglycan_degradation | 0.000925 | 0.000151 | 0.000613 | 0.000289 | 0.048334 | 0.134546 | 2.96E-06 | 0.000623 |
| Metabolism;Biosynthesis of other secondary metabolites;Novobiocin_biosynthesis | 0.001016 | 5.43E-05 | 0.000844 | 0.000111 | 0.010474 | 0.090629 | 5.42E-05 | 0.000291 |
| Metabolism;Biosynthesis of other secondary metabolites;Tropane,_piperidine_and_pyridine_alkaloid_biosynthesis | 0.001043 | 6.63E-05 | 0.000839 | 0.00011 | 0.004473 | 0.070819 | 8.29E-05 | 0.000324 |
| Cellular Processes;Transport and catabolism;Lysosome | 0.00084 | 0.0002 | 0.000481 | 0.000212 | 0.013085 | 0.090629 | 9.33E-05 | 0.000623 |
| Metabolism;Carbohydrate metabolism;Ascorbate_and_aldarate_metabolism | 0.000764 | 6.21E-05 | 0.000573 | 0.000134 | 0.015269 | 0.094612 | 4.95E-05 | 0.000334 |
| Metabolism;Metabolism of terpenoids and polyketides;Biosynthesis_of_ansamycins | 0.000844 | 2.70E-05 | 0.000795 | 3.53E-05 | 0.022916 | 0.119455 | 8.50E-06 | 9.01E-05 |
| Environmental Information Processing;Signal transduction;AMPK_signaling_pathway | 0.000694 | 4.69E-05 | 0.000612 | 4.03E-05 | 0.009321 | 0.087226 | 2.51E-05 | 0.000138 |
| Unclassified;Metabolism;Glycan_biosynthesis_and_metabolism | 0.000642 | 4.08E-05 | 0.000535 | 5.04E-05 | 0.002677 | 0.069354 | 4.70E-05 | 0.000166 |
| Metabolism;Biosynthesis of other secondary metabolites;Neomycin,_kanamycin_and_gentamicin_biosynthesis | 0.000529 | 2.51E-05 | 0.000498 | 1.48E-05 | 0.034166 | 0.134546 | 2.89E-06 | 5.76E-05 |
| Metabolism;Biosynthesis of other secondary metabolites;Glucosinolate_biosynthesis | 0.000341 | 3.28E-05 | 0.000253 | 3.57E-05 | 0.001223 | 0.069354 | 4.43E-05 | 0.000133 |
| Human Diseases;Cancers;Renal_cell_carcinoma | 0.000173 | 2.10E-05 | 0.000265 | 7.42E-05 | 0.027875 | 0.123562 | -0.00017 | ###### |
| Metabolism;Lipid metabolism;Steroid_hormone_biosynthesis | 0.000174 | 6.14E-05 | 6.77E-05 | 3.33E-05 | 0.00616 | 0.07585 | 4.02E-05 | 0.000173 |
| Organismal Systems;Digestive system;Carbohydrate_digestion_and_absorption | 0.000114 | 3.23E-05 | 6.80E-05 | 2.74E-05 | 0.024285 | 0.121816 | 7.38E-06 | 8.47E-05 |
| Metabolism;Biosynthesis of other secondary metabolites;Flavone_and_flavonol_biosynthesis | 0.000118 | 8.70E-06 | 9.90E-05 | 1.79E-05 | 0.047375 | 0.134546 | 2.94E-07 | 3.85E-05 |
| Metabolism;Enzyme families;Cytochrome_P450 | 7.32E-05 | 2.56E-05 | 4.52E-05 | 1.33E-05 | 0.047224 | 0.134546 | 4.43E-07 | 5.55E-05 |
| Metabolism;Xenobiotics biodegradation and metabolism;Steroid_degradation | 4.60E-05 | 2.10E-05 | 2.35E-05 | 6.47E-06 | 0.04649 | 0.134546 | 4.85E-07 | 4.46E-05 |
| Human Diseases;Infectious diseases;African_trypanosomiasis | 3.10E-05 | 1.36E-05 | 1.14E-05 | 8.24E-06 | 0.016221 | 0.094612 | 4.65E-06 | 3.44E-05 |
| Human Diseases;Infectious diseases;Chagas_disease_(American_trypanosomiasis) | 2.83E-05 | 1.31E-05 | 8.78E-06 | 7.11E-06 | 0.013033 | 0.090629 | 5.41E-06 | 3.37E-05 |
| Human Diseases;Neurodegenerative diseases;Prion_diseases | 2.71E-05 | 3.06E-06 | 2.13E-05 | 4.88E-06 | 0.037345 | 0.134546 | 4.31E-07 | 1.12E-05 |
| Genetic Information Processing;Translation;mRNA_surveillance_pathway | 6.58E-07 | 4.11E-07 | 1.73E-07 | 1.07E-07 | 0.033088 | 0.134546 | 5.52E-08 | 9.15E-07 |
| Human Diseases;Immune diseases;Systemic_lupus_erythematosus | 5.61E-07 | 2.55E-07 | 2.84E-07 | 1.41E-07 | 0.049627 | 0.134546 | 5.77E-10 | 5.52E-07 |
| Human Diseases;Infectious diseases;Vibrio_cholerae_infection | 7.42E-07 | 4.06E-07 | 1.88E-07 | 8.80E-08 | 0.019479 | 0.107933 | 1.29E-07 | 9.79E-07 |
| Organismal Systems;Endocrine system;Ovarian_Steroidogenesis | 3.18E-07 | 2.43E-07 | 5.52E-08 | 4.16E-08 | 0.045175 | 0.134546 | 8.14E-09 | 5.17E-07 |
| Environmental Information Processing;Signaling molecules and interaction;G_protein-coupled_receptors | 2.34E-08 | 1.07E-08 | 4.33E-09 | 9.00E-09 | 0.007765 | 0.086047 | 6.32E-09 | 3.19E-08 |
| Organismal Systems;Excretory system;Vasopressin-regulated_water_reabsorption | 4.89E-08 | 3.88E-08 | 6.63E-09 | 6.05E-09 | 0.044322 | 0.134546 | 1.55E-09 | 8.29E-08 |
| Organismal Systems;Nervous system;Synaptic_vesicle_cycle | 4.89E-08 | 3.88E-08 | 6.63E-09 | 6.05E-09 | 0.044322 | 0.134546 | 1.55E-09 | 8.29E-08 |
| Organismal Systems;Immune system;Hematopoietic_cell_lineage | 3.63E-08 | 2.98E-08 | 3.89E-09 | 6.43E-09 | 0.044217 | 0.134546 | 1.20E-09 | 6.36E-08 |
| Environmental Information Processing;Signaling molecules and interaction;Neuroactive_ligand-receptor_interaction | 5.88E-09 | 4.51E-09 | 8.81E-10 | 1.21E-09 | 0.041204 | 0.134546 | 2.81E-10 | 9.72E-09 |
| Metabolism;Glycan biosynthesis and metabolism;Glycosaminoglycan_biosynthesis-chondroitin_sulfate/dermatan_sulfate | 2.39E-09 | 1.68E-09 | 5.30E-10 | 5.60E-10 | 0.041994 | 0.134546 | 9.26E-11 | 3.62E-09 |
| Metabolism;Biosynthesis of other secondary metabolites;Clavulanic_acid_biosynthesis | 7.90E-10 | 6.83E-10 | 1.51E-11 | 1.23E-11 | 0.038954 | 0.134546 | 5.80E-11 | 1.49E-09 |

| Table 2e Old+PBS VS Old+Young | | | | | | | | |
| --- | --- | --- | --- | --- | --- | --- | --- | --- |
| Taxa | avg(Old+PBS) | sd(Old+PBS) | avg(Old+Young) | sd(Old+Young) | p.value | q.values | interval lower | interval upper |
| Genetic Information Processing;Replication and repair;DNA_replication_proteins | 0.015186 | 0.000287 | 0.014866 | 9.1E-05 | 0.040875 | 0.279366 | 1.84E-05 | 0.00062 |
| Metabolism;Carbohydrate metabolism;Pyruvate_metabolism | 0.012992 | 0.000385 | 0.013656 | 0.000423 | 0.01754 | 0.254192 | -0.00119 | -0.00014 |
| Genetic Information Processing;Replication and repair;Nucleotide_excision_repair | 0.008575 | 0.000252 | 0.008078 | 0.000267 | 0.007815 | 0.243757 | 0.000163 | 0.000832 |
| Metabolism;Amino acid metabolism;Glycine,_serine_and_threonine_metabolism | 0.007344 | 0.000264 | 0.00785 | 0.000189 | 0.004085 | 0.217068 | -0.00081 | -0.00021 |
| Metabolism;Carbohydrate metabolism;Butanoate_metabolism | 0.006856 | 0.000234 | 0.00723 | 0.000214 | 0.016388 | 0.248807 | -0.00066 | -8.5E-05 |
| Metabolism;Amino acid metabolism;Lysine_biosynthesis | 0.006577 | 0.000222 | 0.006169 | 0.000258 | 0.015102 | 0.248807 | 9.81E-05 | 0.00072 |
| Cellular Processes;Cell motility;Bacterial_chemotaxis | 0.006541 | 0.000982 | 0.005025 | 0.001103 | 0.030986 | 0.279366 | 0.00017 | 0.002862 |
| Metabolism;Carbohydrate metabolism;Fructose_and_mannose_metabolism | 0.005034 | 0.000448 | 0.005551 | 0.000339 | 0.049861 | 0.29823 | -0.00103 | -3.9E-07 |
| Cellular Processes;Cell growth and death;Cell_cycle-Caulobacter | 0.005301 | 0.000205 | 0.005009 | 0.000152 | 0.020387 | 0.26536 | 5.65E-05 | 0.000527 |
| Metabolism;Lipid metabolism;Lipid_biosynthesis_proteins | 0.004195 | 0.000254 | 0.004486 | 8.15E-05 | 0.036704 | 0.279366 | -0.00056 | -2.5E-05 |
| Metabolism;Metabolism of terpenoids and polyketides;Terpenoid_backbone_biosynthesis | 0.004511 | 0.000132 | 0.004343 | 3.68E-05 | 0.024962 | 0.274426 | 2.99E-05 | 0.000306 |
| Metabolism;Lipid metabolism;Glycerophospholipid_metabolism | 0.00449 | 0.00018 | 0.004242 | 0.000119 | 0.020808 | 0.26536 | 4.8E-05 | 0.000449 |
| Human Diseases;Drug resistance;beta-Lactam_resistance | 0.00351 | 0.000255 | 0.004224 | 0.000625 | 0.037756 | 0.279366 | -0.00137 | -5.4E-05 |
| Metabolism;Amino acid metabolism;Valine,_leucine_and_isoleucine_biosynthesis | 0.00317 | 0.000297 | 0.00354 | 8.59E-05 | 0.026938 | 0.279366 | -0.00068 | -6E-05 |
| Metabolism;Energy metabolism;Photosynthesis_proteins | 0.00297 | 8.72E-05 | 0.002711 | 0.000103 | 0.000901 | 0.153405 | 0.000136 | 0.000382 |
| Metabolism;Energy metabolism;Photosynthesis | 0.002958 | 8.61E-05 | 0.002693 | 0.000108 | 0.000962 | 0.153405 | 0.000138 | 0.000391 |
| Metabolism;Amino acid metabolism;Valine,_leucine_and_isoleucine_degradation | 0.002337 | 0.00011 | 0.002675 | 0.000237 | 0.015654 | 0.248807 | -0.00059 | -8.6E-05 |
| Cellular Processes;Cell motility;Cytoskeleton_proteins | 0.002926 | 6.67E-05 | 0.002601 | 0.000202 | 0.009175 | 0.243757 | 0.000114 | 0.000537 |
| Metabolism;Amino acid metabolism;Histidine_metabolism | 0.002115 | 0.000282 | 0.002464 | 5.24E-05 | 0.028246 | 0.279366 | -0.00064 | -5.4E-05 |
| Metabolism;Amino acid metabolism;Phenylalanine_metabolism | 0.00157 | 9.34E-05 | 0.001676 | 4.86E-05 | 0.04133 | 0.279366 | -0.00021 | -5.4E-06 |
| Metabolism;Metabolism of other amino acids;D-Glutamine_and_D-glutamate_metabolism | 0.001524 | 5.98E-05 | 0.001445 | 3.28E-05 | 0.022738 | 0.274426 | 1.43E-05 | 0.000144 |
| Metabolism;Metabolism of cofactors and vitamins;Vitamin_B6_metabolism | 0.001084 | 0.000119 | 0.001216 | 4.67E-05 | 0.041152 | 0.279366 | -0.00026 | -7.2E-06 |
| Unclassified;Metabolism;Carbohydrate_metabolism | 0.001019 | 0.000118 | 0.001209 | 6.85E-05 | 0.008989 | 0.243757 | -0.00032 | -6.2E-05 |
| Metabolism;Metabolism of other amino acids;beta-Alanine_metabolism | 0.000946 | 6.77E-05 | 0.001021 | 3.03E-05 | 0.042008 | 0.279366 | -0.00015 | -3.6E-06 |
| Environmental Information Processing;Signaling molecules and interaction;Bacterial_toxins | 0.000835 | 0.000179 | 0.001174 | 0.000293 | 0.041303 | 0.279366 | -0.00066 | -1.7E-05 |
| Metabolism;Glycan biosynthesis and metabolism;Glycosaminoglycan_degradation | 0.000591 | 0.000218 | 0.000871 | 4.73E-05 | 0.02479 | 0.274426 | -0.00051 | -5.1E-05 |
| Metabolism;Biosynthesis of other secondary metabolites;Tropane,_piperidine_and_pyridine_alkaloid_biosynthesis | 0.000805 | 7.65E-05 | 0.0009 | 6.07E-05 | 0.039433 | 0.279366 | -0.00018 | -5.7E-06 |
| Cellular Processes;Transport and catabolism;Lysosome | 0.000516 | 0.000241 | 0.000789 | 5.37E-05 | 0.03826 | 0.279366 | -0.00053 | -2.1E-05 |
| Metabolism;Carbohydrate metabolism;Ascorbate_and_aldarate_metabolism | 0.000622 | 8.97E-05 | 0.000961 | 0.000238 | 0.015635 | 0.248807 | -0.00059 | -8.9E-05 |
| Metabolism;Metabolism of terpenoids and polyketides;Biosynthesis_of_ansamycins | 0.00079 | 5.17E-05 | 0.000864 | 5.14E-05 | 0.031053 | 0.279366 | -0.00014 | -8.3E-06 |
| Unclassified;Metabolism;Glycan_biosynthesis_and_metabolism | 0.000512 | 6.35E-05 | 0.00064 | 4.16E-05 | 0.002866 | 0.217068 | -0.0002 | -5.7E-05 |
| Metabolism;Lipid metabolism;Secondary_bile_acid_biosynthesis | 0.000417 | 6.43E-05 | 0.000317 | 3.16E-05 | 0.010832 | 0.248807 | 3.07E-05 | 0.000168 |
| Metabolism;Biosynthesis of other secondary metabolites;Glucosinolate_biosynthesis | 0.000265 | 2.64E-05 | 0.000301 | 1.19E-05 | 0.019076 | 0.264435 | -6.4E-05 | -7.9E-06 |
| Metabolism;Glycan biosynthesis and metabolism;Glycosylphosphatidylinositol_(GPI)-anchored_proteins | 0.000188 | 5.55E-05 | 0.000272 | 2.39E-05 | 0.012006 | 0.248807 | -0.00014 | -2.5E-05 |
| Human Diseases;Cancers;Proteoglycans_in_cancer | 0.000297 | 3.5E-06 | 0.000286 | 9.84E-06 | 0.049327 | 0.29823 | 4.16E-08 | 2.07E-05 |
| Metabolism;Metabolism of terpenoids and polyketides;Biosynthesis_of_siderophore_group_nonribosomal_peptides | 0.000164 | 3.97E-05 | 0.000268 | 5.31E-05 | 0.003814 | 0.217068 | -0.00016 | -4.3E-05 |
| Human Diseases;Cancers;Renal_cell_carcinoma | 0.000284 | 5.25E-05 | 0.000224 | 9.02E-06 | 0.037456 | 0.279366 | 5.05E-06 | 0.000115 |
| Metabolism;Glycan biosynthesis and metabolism;Glycosaminoglycan_binding_proteins | 0.000106 | 2.46E-05 | 0.000166 | 2.76E-05 | 0.002884 | 0.217068 | -9.3E-05 | -2.6E-05 |
| Organismal Systems;Digestive system;Carbohydrate_digestion_and_absorption | 7.38E-05 | 1.73E-05 | 0.000145 | 4.15E-05 | 0.006776 | 0.243757 | -0.00011 | -2.7E-05 |
| Human Diseases;Infectious diseases;African_trypanosomiasis | 7.93E-06 | 3.38E-06 | 1.96E-05 | 6.83E-06 | 0.006524 | 0.243757 | -1.9E-05 | -4.4E-06 |
| Human Diseases;Infectious diseases;Chagas_disease_(American_trypanosomiasis) | 5.73E-06 | 3.61E-06 | 1.58E-05 | 7.11E-06 | 0.016372 | 0.248807 | -1.8E-05 | -2.4E-06 |
| Organismal Systems;Digestive system;Mineral_absorption | 8.81E-06 | 1.93E-06 | 1.57E-05 | 5.61E-06 | 0.028509 | 0.279366 | -1.3E-05 | -1E-06 |
| Environmental Information Processing;Signaling molecules and interaction;Cell_adhesion_molecules_and_their_ligands | 3.64E-06 | 2.05E-06 | 8.03E-06 | 4.03E-06 | 0.046657 | 0.291674 | -8.7E-06 | -8.4E-08 |
| Organismal Systems;Endocrine system;Relaxin_signaling_pathway | 3.44E-06 | 1.9E-06 | 7.31E-06 | 3.46E-06 | 0.043812 | 0.279366 | -7.6E-06 | -1.4E-07 |
| Environmental Information Processing;Signaling molecules and interaction;ECM-receptor_interaction | 3.44E-06 | 1.9E-06 | 7.31E-06 | 3.46E-06 | 0.043812 | 0.279366 | -7.6E-06 | -1.4E-07 |
| Cellular Processes;Cell growth and death;Focal_adhesion | 3.44E-06 | 1.9E-06 | 7.31E-06 | 3.46E-06 | 0.043812 | 0.279366 | -7.6E-06 | -1.4E-07 |
| Human Diseases;Endocrine and metabolic diseases;AGE-RAGE_signaling_pathway_in_diabetic_complications | 3.44E-06 | 1.9E-06 | 7.31E-06 | 3.46E-06 | 0.043812 | 0.279366 | -7.6E-06 | -1.4E-07 |
| Metabolism;Biosynthesis of other secondary metabolites;Stilbenoid,_diarylheptanoid_and_gingerol_biosynthesis | 1.76E-06 | 9.04E-07 | 3.18E-06 | 7.56E-07 | 0.015052 | 0.248807 | -2.5E-06 | -3.4E-07 |
| Metabolism;Biosynthesis of other secondary metabolites;Flavonoid_biosynthesis | 1.76E-06 | 9.04E-07 | 3.18E-06 | 7.56E-07 | 0.015052 | 0.248807 | -2.5E-06 | -3.4E-07 |
| Human Diseases;Immune diseases;Systemic_lupus_erythematosus | 2.46E-07 | 9.81E-08 | 8.68E-07 | 3.66E-07 | 0.007701 | 0.243757 | -1E-06 | -2.4E-07 |
| Human Diseases;Infectious diseases;Pathogenic_Escherichia_coli_infection | 1.71E-07 | 8.14E-08 | 3.99E-07 | 1.87E-07 | 0.029627 | 0.279366 | -4.3E-07 | -3E-08 |
| Organismal Systems;Endocrine system;Ovarian_Steroidogenesis | 6.29E-08 | 5.25E-08 | 1.3E-07 | 4.22E-08 | 0.036164 | 0.279366 | -1.3E-07 | -5.3E-09 |
| Environmental Information Processing;Signaling molecules and interaction;G_protein-coupled_receptors | 2.05E-09 | 3.26E-09 | 3.22E-08 | 2.31E-08 | 0.023419 | 0.274426 | -5.4E-08 | -6E-09 |

| Table 2f Aged+PBS VS Aged+Young | | | | | | | | |
| --- | --- | --- | --- | --- | --- | --- | --- | --- |
| Taxa | avg(Aged+PBS) | sd(Aged+PBS) | avg(Aged+Young) | sd(Aged+Young) | p.value | q.values | interval lower | interval upper |
| Genetic Information Processing;Replication and repair;DNA_replication_proteins | 0.015109 | 0.000133 | 0.014468 | 0.000277 | 0.001287 | 0.083073 | 0.000346 | 0.000937 |
| Metabolism;Carbohydrate metabolism;Pyruvate_metabolism | 0.013256 | 0.000309 | 0.013857 | 0.000478 | 0.030863 | 0.211941 | -0.00113 | ###### |
| Genetic Information Processing;Replication and repair;Mismatch_repair | 0.011191 | 0.000174 | 0.010727 | 0.000429 | 0.045615 | 0.211941 | 1.22E-05 | 0.000917 |
| Environmental Information Processing;Membrane transport;Secretion_system | 0.010266 | 0.000857 | 0.00902 | 0.000808 | 0.026975 | 0.211941 | 0.000174 | 0.002318 |
| Unclassified;Genetic information processing;Replication,_recombination_and_repair_proteins | 0.009811 | 0.000476 | 0.00863 | 0.001048 | 0.0403 | 0.211941 | 6.92E-05 | 0.002293 |
| Metabolism;Energy metabolism;Carbon_fixation_pathways_in_prokaryotes | 0.008631 | 0.000453 | 0.009592 | 0.000683 | 0.019043 | 0.185973 | -0.00172 | -0.0002 |
| Genetic Information Processing;Translation;Messenger_RNA_Biogenesis | 0.007653 | 0.000128 | 0.008007 | 0.000249 | 0.015963 | 0.185973 | -0.00062 | ###### |
| Genetic Information Processing;Folding, sorting and degradation;RNA_degradation | 0.007297 | 0.000143 | 0.007737 | 0.000271 | 0.008643 | 0.164618 | -0.00073 | -0.00015 |
| Metabolism;Energy metabolism;Methane_metabolism | 0.007085 | 0.000104 | 0.00744 | 0.000299 | 0.032733 | 0.211941 | -0.00067 | ###### |
| Metabolism;Carbohydrate metabolism;Butanoate_metabolism | 0.006828 | 0.000131 | 0.007411 | 0.000501 | 0.034917 | 0.211941 | -0.00111 | ###### |
| Metabolism;Carbohydrate metabolism;Citrate_cycle_(TCA_cycle) | 0.005779 | 0.000294 | 0.006533 | 0.000662 | 0.038681 | 0.211941 | -0.00146 | ###### |
| Unclassified;Metabolism;Energy_metabolism | 0.005571 | 0.00032 | 0.006071 | 0.000169 | 0.010204 | 0.164618 | -0.00084 | -0.00016 |
| Metabolism;Energy metabolism;Carbon_fixation_in_photosynthetic_organisms | 0.004982 | 4.40E-05 | 0.005252 | 0.000113 | 0.001201 | 0.083073 | -0.00039 | -0.00015 |
| Metabolism;Amino acid metabolism;Arginine_biosynthesis | 0.004855 | 0.000135 | 0.005175 | 0.000167 | 0.004703 | 0.13006 | -0.00052 | -0.00012 |
| Metabolism;Lipid metabolism;Glycerophospholipid_metabolism | 0.004402 | 0.00011 | 0.00409 | 0.000279 | 0.04047 | 0.211941 | 1.83E-05 | 0.000607 |
| Metabolism;Glycan biosynthesis and metabolism;Glycosyltransferases | 0.003989 | 0.000158 | 0.003766 | 0.000147 | 0.030047 | 0.211941 | 2.63E-05 | 0.000419 |
| Metabolism;Amino acid metabolism;Valine,_leucine_and_isoleucine_biosynthesis | 0.003302 | 0.000124 | 0.003687 | 0.000289 | 0.020583 | 0.185973 | -0.00069 | ###### |
| Metabolism;Energy metabolism;Sulfur_metabolism | 0.00237 | 6.26E-05 | 0.002678 | 0.000107 | 0.000281 | 0.054485 | -0.00042 | -0.00019 |
| Metabolism;Amino acid metabolism;Histidine_metabolism | 0.002265 | 6.44E-05 | 0.002736 | 0.000321 | 0.014798 | 0.185973 | -0.00081 | -0.00014 |
| Metabolism;Carbohydrate metabolism;C5-Branched_dibasic_acid_metabolism | 0.001784 | 8.80E-05 | 0.001976 | 0.000135 | 0.018159 | 0.185973 | -0.00034 | ###### |
| Metabolism;Amino acid metabolism;Phenylalanine_metabolism | 0.001538 | 7.37E-05 | 0.00177 | 0.000109 | 0.001977 | 0.095695 | -0.00035 | -0.00011 |
| Unclassified;Metabolism;Amino_acid_metabolism | 0.001107 | 4.00E-05 | 0.001305 | 0.000104 | 0.004041 | 0.13006 | -0.00031 | ###### |
| Unclassified;Viral protein family;Viral_proteins | 0.001018 | 4.82E-05 | 0.000951 | 5.44E-05 | 0.046274 | 0.211941 | 1.36E-06 | 0.000134 |
| Metabolism;Biosynthesis of other secondary metabolites;Novobiocin_biosynthesis | 0.000857 | 5.38E-05 | 0.000986 | 9.59E-05 | 0.021135 | 0.185973 | -0.00023 | ###### |
| Metabolism;Biosynthesis of other secondary metabolites;Tropane,_piperidine_and_pyridine_alkaloid_biosynthesis | 0.000836 | 5.62E-05 | 0.000952 | 9.70E-05 | 0.034092 | 0.211941 | -0.00022 | ###### |
| Human Diseases;Endocrine and metabolic diseases;Type_I_diabetes_mellitus | 0.000693 | 8.78E-06 | 0.000723 | 2.70E-05 | 0.038661 | 0.211941 | ###### | ###### |
| Metabolism;Biosynthesis of other secondary metabolites;Carbapenem_biosynthesis | 0.000657 | 3.20E-05 | 0.000715 | 3.61E-05 | 0.015544 | 0.185973 | -0.0001 | ###### |
| Human Diseases;Immune diseases;Primary_immunodeficiency | 0.000376 | 1.44E-05 | 0.000344 | 2.31E-05 | 0.01887 | 0.185973 | 6.82E-06 | 5.76E-05 |
| Metabolism;Lipid metabolism;Secondary_bile_acid_biosynthesis | 0.000359 | 2.94E-05 | 0.000324 | 2.35E-05 | 0.045604 | 0.211941 | 8.46E-07 | 6.97E-05 |
| Metabolism;Biosynthesis of other secondary metabolites;Glucosinolate_biosynthesis | 0.000272 | 1.21E-05 | 0.000331 | 3.73E-05 | 0.009543 | 0.164618 | ###### | ###### |
| Metabolism;Xenobiotics biodegradation and metabolism;Atrazine_degradation | 0.000234 | 5.77E-05 | 0.000163 | 5.24E-05 | 0.048173 | 0.211941 | 7.01E-07 | 0.000143 |
| Human Diseases;Neurodegenerative diseases;Huntingtons_disease | 0.000223 | 5.85E-05 | 0.000161 | 1.05E-05 | 0.047924 | 0.211941 | 8.15E-07 | 0.000123 |
| Metabolism;Xenobiotics biodegradation and metabolism;Xylene_degradation | 0.00019 | 1.18E-05 | 0.000164 | 1.55E-05 | 0.009188 | 0.164618 | 8.12E-06 | 4.38E-05 |
| Genetic Information Processing;Folding, sorting and degradation;Ubiquitin_system | 0.00016 | 4.01E-06 | 0.000146 | 8.65E-06 | 0.008999 | 0.164618 | 4.70E-06 | 2.31E-05 |
| Metabolism;Xenobiotics biodegradation and metabolism;Toluene_degradation | 0.00011 | 1.13E-05 | 8.93E-05 | 1.90E-05 | 0.04628 | 0.211941 | 4.43E-07 | 4.19E-05 |
| Metabolism;Xenobiotics biodegradation and metabolism;Caprolactam_degradation | 6.02E-05 | 1.83E-05 | 9.04E-05 | 2.37E-05 | 0.034398 | 0.211941 | ###### | ###### |
| Metabolism;Lipid metabolism;Ether_lipid_metabolism | 6.40E-05 | 2.06E-05 | 4.06E-05 | 7.48E-06 | 0.038507 | 0.211941 | 1.69E-06 | 4.50E-05 |
| Metabolism;Lipid metabolism;alpha-Linolenic_acid_metabolism | 5.48E-05 | 1.65E-05 | 3.19E-05 | 7.59E-06 | 0.017643 | 0.185973 | 5.35E-06 | 4.04E-05 |
| Human Diseases;Cancers;Small_cell_lung_cancer | 4.15E-06 | 1.57E-06 | 6.28E-06 | 9.43E-07 | 0.020795 | 0.185973 | ###### | ###### |
| Genetic Information Processing;Replication and repair;Fanconi_anemia_pathway | 1.22E-06 | 2.53E-07 | 9.36E-07 | 1.65E-07 | 0.047393 | 0.211941 | 4.11E-09 | 5.66E-07 |
| Human Diseases;Cancers;Bladder_cancer | 8.40E-07 | 2.57E-07 | 1.27E-06 | 3.32E-07 | 0.032744 | 0.211941 | ###### | ###### |
| Metabolism;Metabolism of terpenoids and polyketides;Biosynthesis_of_type_II_polyketide_backbone | 2.91E-07 | 4.49E-08 | 6.86E-07 | 1.91E-07 | 0.003227 | 0.124925 | ###### | ###### |
| Organismal Systems;Excretory system;Vasopressin-regulated_water_reabsorption | 5.81E-09 | 5.23E-09 | 2.11E-08 | 1.43E-08 | 0.048109 | 0.211941 | ###### | ###### |
| Organismal Systems;Nervous system;Synaptic_vesicle_cycle | 5.81E-09 | 5.23E-09 | 2.11E-08 | 1.43E-08 | 0.048109 | 0.211941 | ###### | ###### |
